# Surface‐State‐Driven Selectivity of Electrocatalytic Glucose Oxidation: Metallic Au, Au─OH, and CuO

**DOI:** 10.1002/advs.78627

**Published:** 2026-11-05

**Authors:** Myeonghwan Oh, Hoeun Seong, Ga Eun Jo, Jiyoung Hwang, Yurim Sohn, Kyoungsuk Jin, Jong Hyeok Park, Wooyul Kim, Seok Ki Kim, Dong Ki Lee

**Affiliations:** ^1^ Clean Energy Research Center Korea Institute of Science and Technology (KIST) Seoul Republic of Korea; ^2^ Department of Chemistry Korea University Seoul Republic of Korea; ^3^ Department of Chemical Engineering Ajou University Suwon Republic of Korea; ^4^ Department of Energy Engineering Korea Institute of Energy Technology Naju Republic of Korea; ^5^ Department of Chemical and Biomolecular Engineering Yonsei‐KIST Convergence Research Institute Yonsei University Seoul Republic of Korea; ^6^ Graduate School of Energy and Environment Korea University Seoul Republic of Korea

**Keywords:** bond cleavage, copper, electrocatalyst, electrochemistry, formic acid, gluconic acid, gold particles, l‐glucose

## Abstract

The electrochemical glucose oxidation reaction (GLUOR) offers a sustainable route for biomass valorization, yet its reaction mechanisms and the factors governing product selectivity remain poorly understood. Herein, we present a comprehensive mechanistic investigation of GLUOR on Au and CuO catalysts. Au catalysts preserve the C6 carbon backbone while exhibiting surface‐state‐dependent oxidation of terminal functional groups of glucose (GLU), whereas CuO catalysts preferentially promote C─C bond cleavage reactions. Theoretical investigation reveals that C─C bond cleavage is energetically unfavorable on Au surfaces, in either the metallic or the hydroxylated state, but it is thermodynamically preferred on CuO surfaces. Furthermore, we provide spectroscopic and computational evidence that pyranose glucose undergoes ring opening on metallic Au surfaces, while ring‐retained pathways dominate on Au─OH and CuO surfaces, leading to gluconolactone formation and C─C bond cleavage, respectively. These findings provide a fundamental framework for interpreting product selectivity in glucose oxidation and offer mechanistic insights applicable to a broad range of biomass valorization processes.

## Graphical abstract

In the electrochemical oxidation of glucose, whether the pyranose ring opens upon adsorption decides the reaction pathway: ring opening on metallic Au yields gluconic acid, gluconolactone formation on Au─OH leads to glucaric acid, and C─C cleavage on CuO gives formic acid. Surface state thus governs product selectivity.

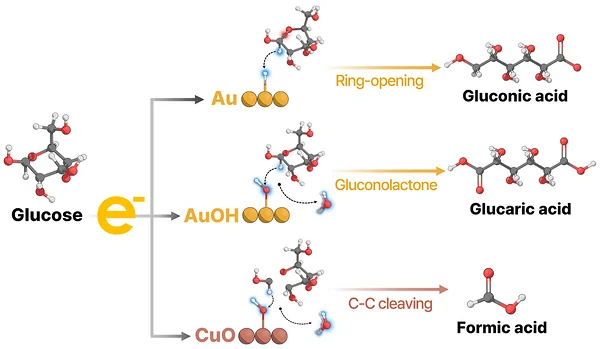

## 1 Introduction

Biomass‐derived carbohydrates have emerged as sustainable feedstocks for producing value‐added chemicals [1, 2]. In particular, glucose (GLU), obtained from the hydrolysis of cellulose, can be transformed into industrially important compounds such as gluconic acid (GNA), glucaric acid (GRA), and adipic acid [3, 4, 5]. Among these products, GRA has been recognized as one of the “Top 12 Value‐Added Chemicals from Biomass” owing to its applications as a polymer precursor, detergent builder, and chelating agent [6]. At present, the dominant industrial route for producing GRA relies on nitric‐acid‐based thermocatalytic oxidation of GLU, which suffers from significant NO_x_ emissions and typically affords yields below 50% [7]. Developing green and selective strategies for converting GLU into these platform chemicals is therefore an essential step toward practical biomass valorization.

Electrochemical oxidation provides a promising route for GLU conversion by enabling selective transformations without stoichiometric chemical oxidants under mild operating conditions [8]. When coupled with cathodic reactions such as hydrogen evolution or CO_2_ reduction, the GLU oxidation reaction (GLUOR) can further offer co‐production pathways that enhance overall energy efficiency [9, 10]. However, despite extensive research efforts, reported product selectivity in GLUOR varies widely across the literature (listed in Table S1). For example, Liu et al. and Tayebi et al. reported GRA yields above 70% using NiFe‐based electrocatalysts in 1.0 m KOH solution [10, 11], whereas Zhang et al. observed less than 10% GRA selectivity on NiO alone in 0.1 m K_2_CO_3_ solution, which increased to 83% with a TEMPO mediator [12]. Moreover, recent studies have shown that transition metal‐based catalysts, including Ni(OH)_2_ and Cu(OOH), predominantly favor C─C bond cleavage for GLUOR, producing formic acid as the major product [13, 14, 15, 16, 17, 18, 19, 20, 21]. Although these studies report widely varying selectivities, the origins of these divergent trends remain poorly understood.

The mechanistic understanding of electrocatalytic GLUOR has evolved significantly, yet a unified mechanistic framework is still lacking. For Ni‐ and Cu‐based catalysts, GLU oxidation has been reported to occur predominantly at elevated potentials where surface oxide or oxyhydroxide species are formed, usually leading to extensive C─C bond cleavage and formate production [13, 14, 15, 16, 17, 18, 19, 20, 21]. In contrast, noble‐metal catalysts such as Au exhibit pronounced potential‐ and surface‐state‐dependent behavior, producing oxidized C6 products such as GNA or GRA under specific reaction conditions [17, 19, 22, 23]. Various mechanisms, including ‘dissociative adsorption with ring opening’ [13] and ‘gluconolactone formation followed by OH^−^ attack’ [24], have been proposed to rationalize the electrocatalytic GLUOR on Au catalysts. Nevertheless, current mechanistic interpretations often remain case‐specific, resulting in fragmented or sometimes conflicting conclusions across the literature. A comprehensive understanding of GLUOR therefore requires simultaneous consideration of key parameters such as catalyst composition, surface state, electrolyte environment, and applied potential.

In this work, we report a comprehensive mechanistic study of GLUOR using Au and CuO catalysts. At low potentials (0.8 V vs reversible hydrogen electrode, RHE; hereafter all potentials are referenced to RHE unless stated otherwise), Au predominantly produces GNA, whereas higher potentials (1.1 V) generate Au─OH species that additionally activate alcohol oxidation pathways to form GRA with minor C─C cleavage (Scheme 1a). In sharp contrast, CuO promotes C─C bond cleavage above ≈1.2 V, with formic acid and ≤C5 species (e.g., arabinose and erythrose) as the major products (Scheme 1b). By combining model‐compound oxidation experiments with density functional theory (DFT) calculations, we demonstrate that reaction preferences for aldehyde oxidation, alcohol oxidation, and C─C bond cleavage are governed by the catalyst identity and its surface state. Aldehyde oxidation dominates on Au regardless of its surface state, and GRA forms on Au─OH, whereas CuO favors C─C bond cleavage. In situ infrared (IR) spectroscopy further reveals that whether the pyranose ring of GLU is opened or retained upon adsorption governs the subsequent pathway (aldehyde oxidation, lactone formation, or C─C cleavage). These findings provide mechanistic guidelines for designing electrocatalysts capable of selectively promoting aldehyde or alcohol oxidation reactions.

**SCHEME 1:**
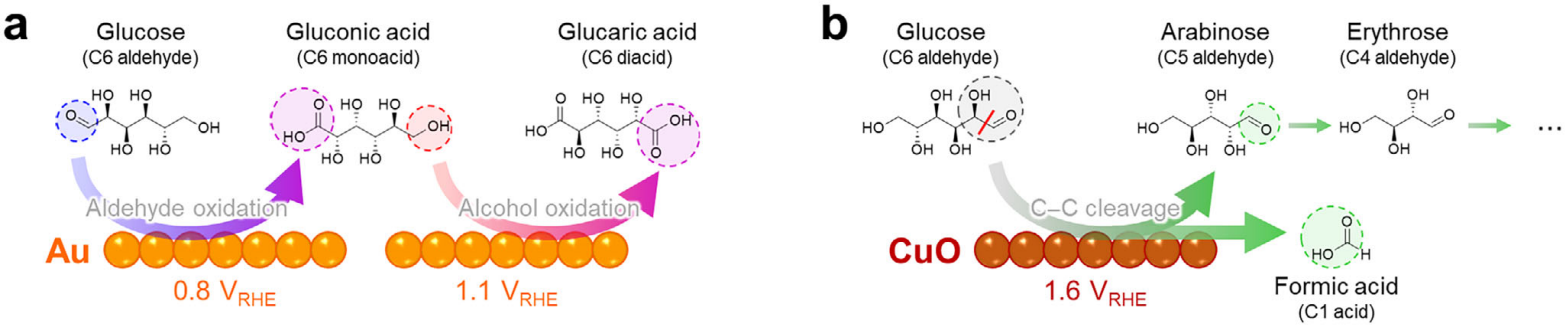
Comparison of glucose electrooxidation pathways on (a) Au and (b) CuO catalysts. On Au, aldehyde oxidation mainly occurs at lower potentials (0.8 V_RHE_), and alcohol oxidation proceeds at higher potentials (1.1 V_RHE_), yielding C6‐retaining carboxylates. On CuO, elevated potentials (1.6 V_RHE_) promote C─C bond cleavage of glucose, generating C1 fragments (formic acid) and ≤C5 aldehydes that undergo subsequent oxidation.

## 2 Results and Discussion

### 2.1 Electrochemical GLUOR on Au and CuO Catalysts

Initially, we evaluated the glucose oxidation activity of various transition and noble metal catalysts (Cu, Ni, Co, Pt, and Au) commonly used for GLUOR (Figure S1). Among the tested candidates, Au predominantly generated C6‐retaining products, such as GNA and GRA, indicating its inherent preference for oxidation pathways that maintain the carbon backbone. Therefore, Au was selected as the primary catalyst to investigate the mechanism of glucose electrooxidation. In contrast, Cu exhibited the highest selectivity toward formic acid, suggesting that it promotes the C─C bond cleavage pathway. Consequently, Cu was employed as a contrasting reference catalyst for a comprehensive mechanistic comparison.

Based on these observations, Au/C‐foam and Cu foam electrodes were employed to investigate GLUOR (see the Experimental Section for details). X‐ray diffraction (XRD) patterns (Figure S2a,b) showed metallic Au, and weak CuO peaks together with the Cu substrate peaks. The CuO surface layer was further confirmed by X‐ray photoelectron spectroscopy (XPS) and scanning electron microscopy (SEM). XPS (Figure S2c,d) of the activated electrodes revealed Au 4f_7/2_ and Cu 2p_3/2_ peaks at 83.7 and 933.4 eV, respectively, assignable to metallic Au (Au^0^) [25] and oxidized Cu species (Cu^2+^) [26]. SEM images (Figure S3) of the activated electrodes revealed distinct surface morphologies for the two catalysts. As shown in Figure S3a,c, Au was deposited on the carbon foam surface as sub‐micrometer particles (ca. 0.2–1 µm). In contrast, the activated Cu electrode exhibited a characteristic needle‐like structure (Figure S3d), indicating the formation of CuO on the copper foam surface.

The GLUOR activities of Au/C‐foam and CuO catalysts were evaluated in 0.1 m Na_2_CO_3_ (pH 11.6) and 0.1 m NaOH (pH 13). In both electrolyte conditions, linear sweep voltammetry (LSV) curves (Figure 1a,b) indicate that both Au and CuO catalysts exhibit lower catalytic onset potentials for GLUOR than for oxygen evolution reaction. Nevertheless, the LSV profiles of Au and CuO differ significantly, reflecting distinct electrocatalytic behaviors of the two catalysts. In a 0.1 m Na_2_CO_3_ solution, Au initiates GLUOR at approximately 0.5 V, followed by a second increase in current at around 0.9 V, and then a sharp decline beyond 1.2 V, which can be attributed to the formation of Au─OH and AuO_x_ surfaces, respectively [27, 28, 29]. In contrast, CuO exhibits a much more positive onset potential of around 1.2 V, after which the current increases sharply with increasing potential. With increasing pH, the catalytic onset potentials of both catalysts for GLUOR shift negatively on the RHE scale, accompanied by an overall increase in current density. Electrochemically active surface area (ECSA)‐normalized LSV curves exhibited a similar trend, with CuO requiring approximately 0.7–0.8 V more positive onset potential than Au, indicating that the activity difference cannot be solely attributed to differences in electrochemically active surface area (Figures S4 and S5). Increasing the GLU concentration from 10 to 50 mm in 0.1 m NaOH enhanced the oxidation current on Au, indicating that the reaction remained sensitive to GLU concentration within the investigated range. The potential of current suppression on Au also shifted positively, possibly due to delayed AuO_x_ formation at higher GLU concentrations. In contrast, the oxidation current on CuO remained nearly unchanged, suggesting a weaker dependence on GLU concentration (Figure S6).

**FIGURE 1:**
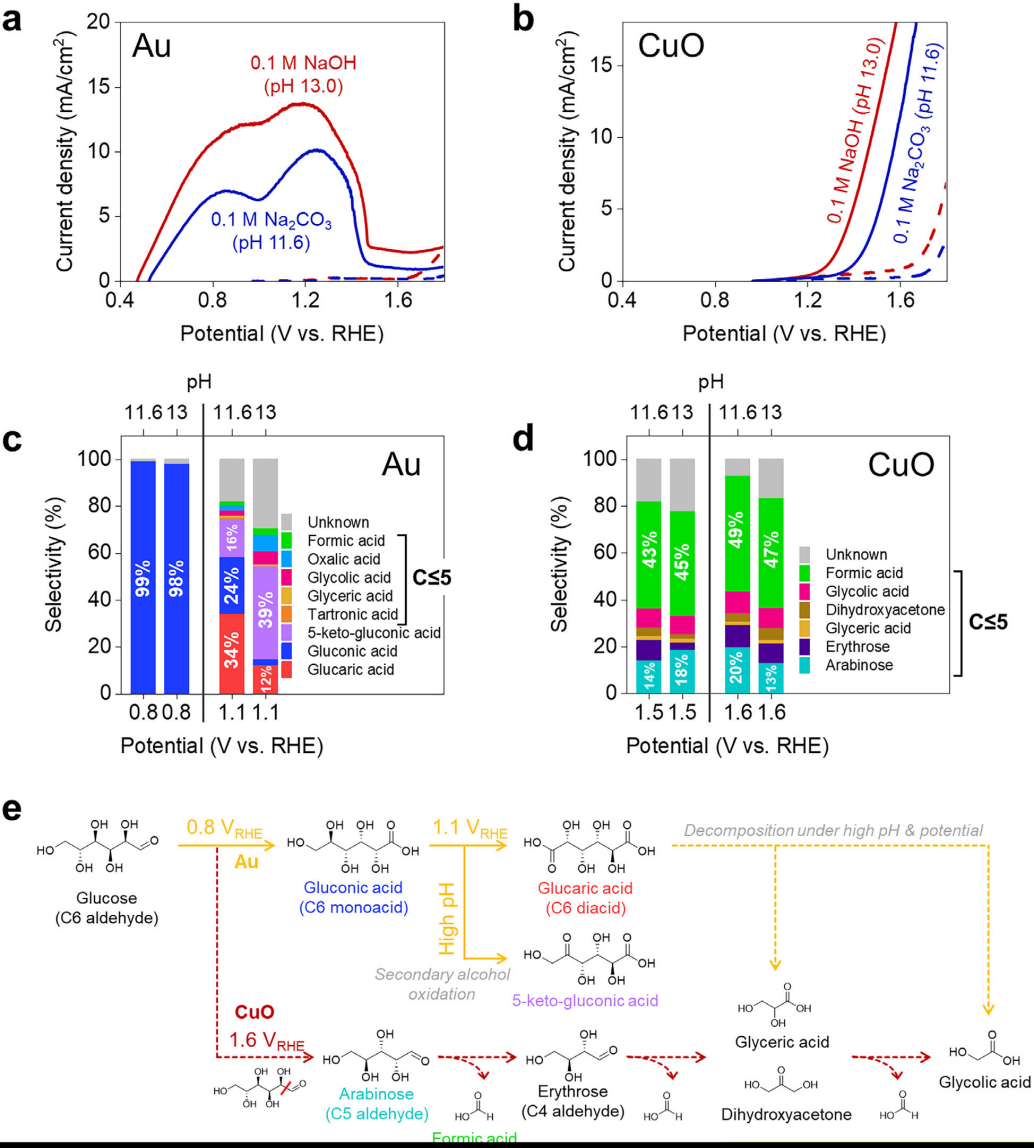
LSV curves recorded at 10 mV s^−1^ on (a) Au/C‐foam and (b) CuO with 10 mm GLU in 0.1 m Na_2_CO_3_ or 0.1 m NaOH solutions. The blank traces (without GLU) are shown as dashed lines. Product distributions at selected potentials after 80 C passed on (c) Au/C‐foam and (d) CuO. (e) Schematic summary of potential‐ and pH‐dependent product pathways on Au and CuO catalysts.

The GLUOR products on Au and CuO catalysts were quantified by high‐performance liquid chromatography (HPLC) after (pulsed) potentiostatic electrolysis (Figure S7). On the Au catalyst (Figure 1c), GNA was formed almost exclusively at 0.8 V (99% selectivity), indicating dominant aldehyde oxidation. At 1.1 V, additional oxidation products were observed, including GRA with a selectivity of 34%, corresponding to oxidation of both the aldehyde and alcohol groups, and 5‐keto‐gluconic acid with a selectivity of 16%, originating from secondary alcohol oxidation. Residual GNA (24%) and trace amounts of C─C bond cleavage products were also detected. This result can be attributed to the formation of surface Au─OH species at higher potentials, which are known to facilitate alcohol dehydrogenation via hydroxyl‐assisted oxidation pathways (vide infra) [30]. Notably, in 0.1 m NaOH solution, the selectivity of 5‐keto‐gluconic acid increased markedly from 16% to 39%, accompanied by an increase in C─C cleaved products, suggesting that secondary alcohol oxidation is favored under highly alkaline conditions. This observation is consistent with the well‐established understanding that secondary alcohol oxidation is promoted under alkaline conditions through alkoxide formation and hydroxyl‐assisted dehydrogenation pathways [31].

Charge‐dependent product analysis further reveals the evolution of product distributions (Figure S8). At 0.8 V, GNA remains the exclusive product over the investigated charge range. In contrast, at 1.1 V, the fraction of deeper oxidation products (GRA, 5‐keto‐gluconic acid, and ≤C5 products) progressively increases as the reaction proceeds. This trend was also observed during 24 h electrolysis, where GRA gradually increased in Na_2_CO_3_, while secondary alcohol oxidation and C─C cleavage became more pronounced in NaOH (Figure S9).

On the CuO catalyst (Figure 1d), however, C─C cleavage products, including arabinose (C5), erythrose (C4), and formic acid (C1) were dominant irrespective of the applied potential and electrolyte pH. As the reaction proceeded (Figure S10), the C─C bonds in the intermediate C5 and C4 species were further cleaved, leading to their gradual decomposition and the accumulation of formic acid as the final product. This trend was observed in both 0.1 m Na_2_CO_3_ and 0.1 m NaOH, as evidenced by decreasing fractions of C5 and C4 products accompanied by an increased fraction of formic acid. Extended electrolysis over 24 h further confirmed that C─C cleaved products remained dominant on CuO, although their distributions varied with the electrolyte conditions (Figure S11). It is worth noting that a previous study [32] reported selective gluconic acid formation (without C─C cleavage) over Cu‐based catalysts at relatively lower anodic potentials. The reference study utilized a Cu(OH)_2_–Cu_2_O redox system, whereas our work employs Cu(OH)_2_ species formed on a thermodynamically stable CuO surface. This fundamental difference in the underlying catalyst structure likely leads to the distinct operating potential windows and major products.

Electrolysis at higher GLU concentrations (30 and 50 mm) in 0.2 m electrolytes showed the same catalyst‐dependent selectivity, with C─C cleavage dominating on CuO and C6 retention on Au (Figures S12 and S13). Although glucose is known to undergo isomerization to fructose in alkaline media, this pathway is unlikely to be significant under our experimental conditions. Fructose was not detected in our analysis. A fructose oxidation experiment revealed a distinctly different product distribution compared to that of glucose oxidation (Figure S14). Therefore, if fructose had been formed in a substantial amount, the product distribution would have been different. These observations indicate that glucose is predominantly oxidized to GNA or arabinose, rather than being isomerized to fructose.

To examine catalyst stability, electrolysis was repeated for five cycles with fresh electrolyte. The initial current was largely recovered in each cycle, and similar product selectivities were maintained on both catalysts (Figures S15 and S16). After five cycles, SEM revealed morphological changes, including apparent coarsening of Au particles and partial loss of the CuO surface structures, whereas XPS confirmed the retention of metallic Au and Cu^2+^ species (Figures S17 and S18). These results suggest that the distinct product selectivities of Au and CuO are preserved despite morphological changes, highlighting the importance of their surface chemical properties in determining the reaction pathways.

The distinct product pathways of GLUOR on Au and CuO catalysts are summarized in Figure 1e. Au catalysts preferentially oxidize GLU into C6‐retaining products at relatively low potentials with only minor decomposition occurring under high‐potential and highly alkaline conditions. On the other hand, CuO catalysts require high anodic potentials to become active for GLUOR, under which extensive C─C bond cleavage dominates, ultimately yielding formic acid as the major product.

### 2.2 C─C Bond Cleavage during GLUOR

To gain mechanistic insights into the C─C bond cleavage occurring during GLUOR, the free energies of C─C‐cleaved intermediates spanning from C6 to C2 species were calculated (Figures S19 and S20 and Table S2). Au(111) and AuOH(111) surfaces were employed as models to represent metallic Au and hydroxylated Au surfaces, respectively, while the CuO(111) surface was used to describe the CuO catalyst, consistent with the surface oxidation state identified by XPS and XRD analyses in Figure S2. As shown in Figure 2a, we evaluated the competing elementary steps at the initial branching point of glucose oxidation: aldehyde oxidation (toward gluconic acid/gluconate) and C─C bond scission (toward arabinose).

**FIGURE 2:**
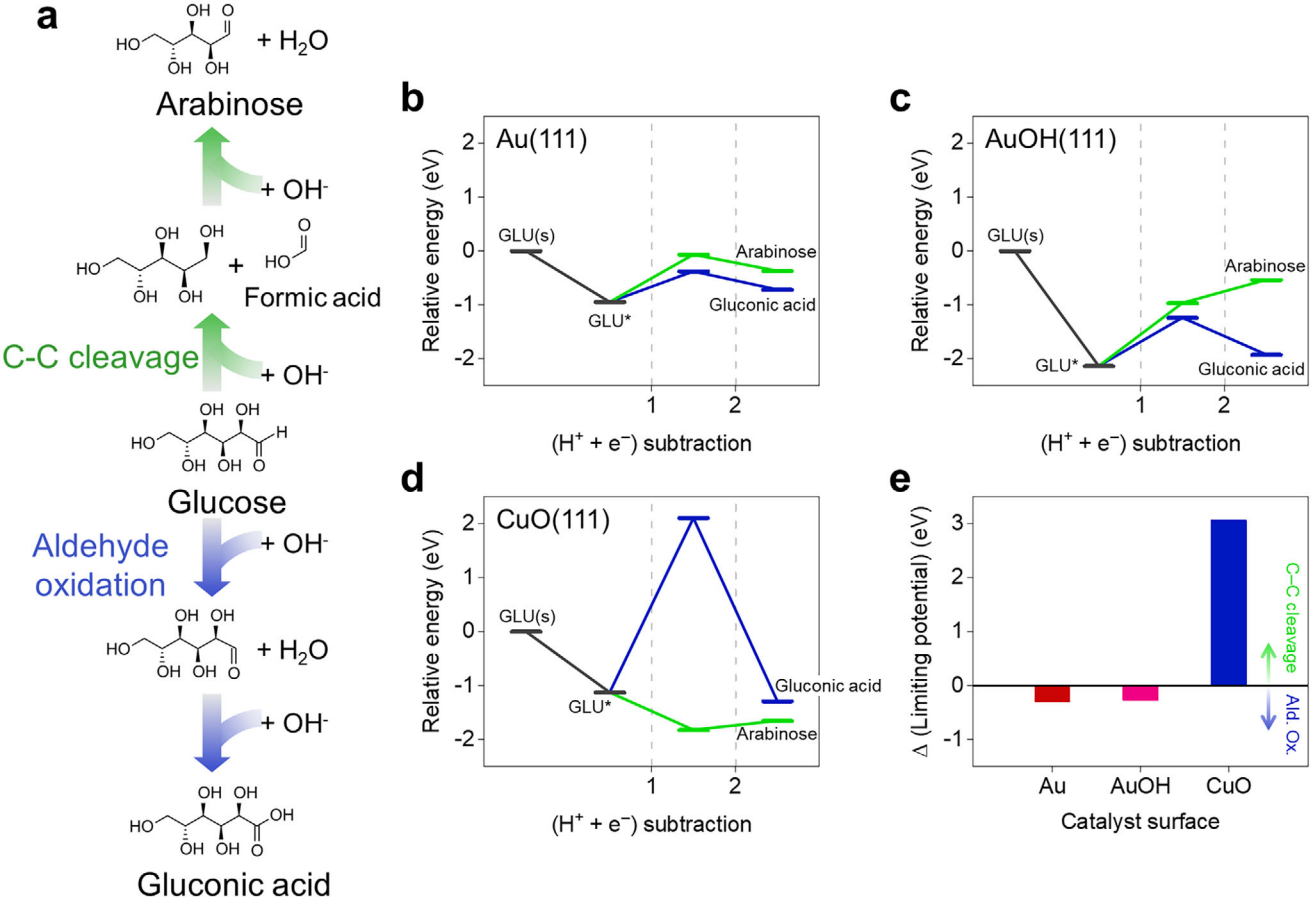
(a) Reaction branching of adsorbed glucose (GLU*) via aldehyde oxidation (blue) versus C─C bond cleavage (green) on Au, AuOH, and CuO surfaces. Free energy diagrams constructed for GLU decomposition pathways on (b) Au(111), (c) AuOH(111), and (d) CuO(111) surfaces calculated at zero applied potential. (e) Differences between the limiting potentials of aldehyde oxidation and C─C bond cleavage. Positive values indicate a preference for C─C bond cleavage, whereas negative values indicate a preference for aldehyde oxidation.

On metallic Au(111), aldehyde oxidation to GNA exhibits a limiting potential of 0.57 eV, whereas C─C bond cleavage requires a substantially higher energy input of 0.88 eV (Figure 2b). A similar trend is observed on the AuOH(111) surface (Figure 2c), where aldehyde oxidation proceeds with a limiting potential of 0.90 eV, while C─C bond cleavage is further disfavored, requiring 1.17 eV. These results indicate that both metallic and hydroxylated Au surfaces intrinsically favor C6‐retaining oxidation pathways over GLU fragmentation under moderate electrochemical conditions. It is noteworthy, however, that the limiting potential defines the thermodynamic accessibility of a given pathway rather than its absolute selectivity. As the applied potential or pH increases, the C─C bond cleavage pathway becomes thermodynamically competitive, consistent with the experimental observation that fragmentation products emerge on Au surfaces only under more oxidizing conditions (Figure 1).

In contrast, the CuO(111) surface exhibits a different energetic landscape. As shown in Figure 2d, aldehyde oxidation on CuO is associated with a very high energy barrier, corresponding to a limiting potential of 3.23 eV. However, C─C bond cleavage from GLU to arabinose proceeds downhill in free energy, with a very small limiting potential of 0.17 eV. These results indicate that CuO intrinsically favors C─C bond cleavage over terminal aldehyde oxidation. This pronounced preference for backbone scission rationalizes the experimentally observed dominance of C─C cleaved products on CuO catalysts, irrespective of the applied potential or electrolyte conditions (Figure 1d).

Taken together, the free energy analysis under zero applied potential reveals a clear catalyst‐dependent divergence in the preferred reaction pathways. Au surfaces, irrespective of their hydroxylation state, disfavor C─C bond cleavage and instead promote aldehyde oxidation while preserving the C6 framework. In contrast, CuO surfaces intrinsically favor C─C bond cleavage over aldehyde oxidation, resulting in a fragmentation of GLU into smaller carbon units. These theoretically predicted thermodynamics provide a clear explanation for the distinct product distributions observed experimentally (Figure 1), with the energetic origin of backbone retention on Au and backbone cleavage on CuO catalysts.

### 2.3 Electrochemical Oxidation of Alcohol and Aldehyde Groups

GLU possesses an aldehyde group at one terminus and a primary alcohol group at the other. Selective oxidation of each functional group yields GNA and glucuronic acid, respectively, while oxidation of both termini produces GRA (Figure 3a). Accordingly, these oxidized derivatives contain well‐defined aldehyde and/or alcohol functionalities and therefore investigating their individual oxidation behaviors provides valuable mechanistic insights into GLUOR. Based on this rationale, LSV curves obtained with GLU and its derivatives on Au and CuO catalysts were compared (Figure 3b,c). To minimize mass‐transport effects, rotating disk electrodes (RDEs) of Au and CuO were used.

**FIGURE 3:**
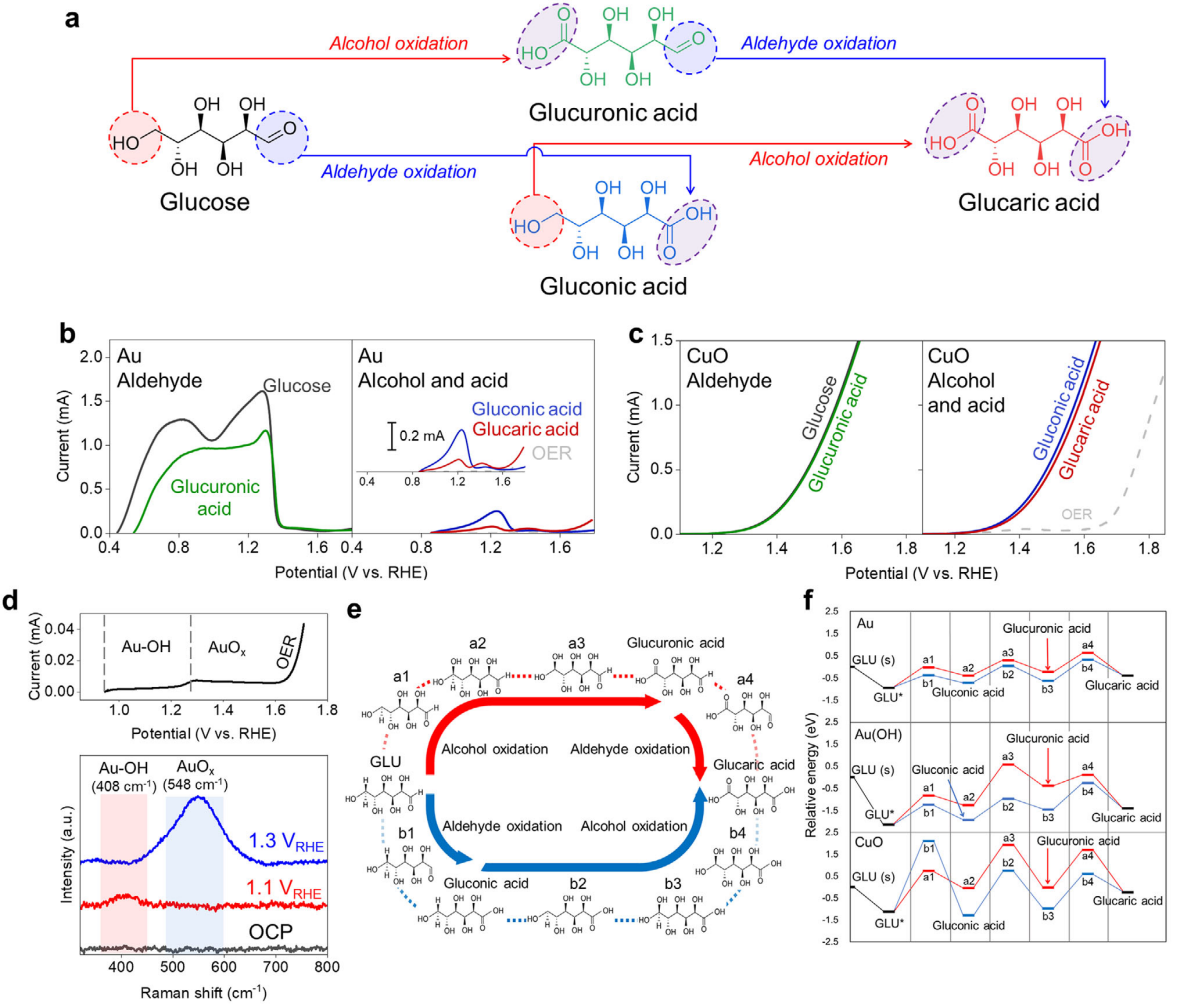
(a) Schematic illustration of the oxidation pathways of GLU and its derivatives. LSV traces recorded on (b) Au and (c) CuO catalysts at 1600 rpm in 0.1 m NaOH containing 10 mm GLU, glucuronic acid, GNA, or GRA. LSV curves measured in the absence of substrates are shown as grey dashed lines for comparison. (d) LSV of Au foil recorded at 10 mV s^−1^ in 0.1 m NaOH without GLU (top) and in situ Raman spectra recorded at open circuit potential (OCP), 1.1 and 1.3 V_RHE_ (bottom). (e) Schematic illustration of the conversion pathways to GNA (C1 oxidation), glucuronic acid (C6 oxidation), and GRA (oxidation at both termini), depending on the oxidation sequence of the terminal functional groups of glucose (C1 aldehyde and C6 primary alcohol). (f) Free energy diagram (referenced to consecutive H^+^/e^−^ removal steps) for the aldehyde‐first (C1 oxidation) and alcohol‐first (C6 oxidation) pathways on Au, Au(OH), and CuO surfaces.

As shown in Figure 3b, the Au catalyst exhibits distinct oxidation behaviors toward aldehyde‐containing and aldehyde‐free compounds. Glucuronic acid, which contains an aldehyde group, displays an oxidation profile similar to that of GLU, although with a slightly more positive onset potential and lower current. This lower current is likely associated with site blocking by the C6 carboxylate, as rhamnose, which bears a terminal CH_3_ group instead, showed a current comparable to that of GLU (Figure S21). In contrast, GNA and GRA, which do not contain aldehyde functionalities, exhibit much lower oxidation currents, primarily at potentials above 0.9 V. This observation is consistent with the product distribution shown in Figure 1c, where alcohol oxidation becomes accessible only at potentials exceeding 0.9 V. These results indicate that Au predominantly catalyzes aldehyde oxidation, while alcohol oxidation occurs to a limited extent at higher potentials.

To probe the potential‐dependent surface state of Au, in situ Raman spectroscopy was performed (Figure 3d). A band at 408 cm^−1^ assigned to Au─OH appeared at 1.1 V, whereas at 1.3 V this band disappeared and a new band at 548 cm^−1^ assigned to AuO_x_ emerged [29]. GNA oxidation becomes appreciable in the potential region associated with Au hydroxylation, while current suppression at higher potentials coincides with AuO_x_ formation. These observations support the involvement of surface Au─OH species in alcohol oxidation on Au.

In contrast, the CuO catalyst displays nearly identical LSV profiles for the oxidation of all investigated compounds. For each molecule, a catalytic onset is observed at approximately 1.2 V, followed by oxidation currents that increase with similar slopes. This behavior indicates that the observed oxidative current on CuO is largely independent of the presence of aldehyde or alcohol groups. It is noteworthy that even GRA, which contains neither an aldehyde nor a primary alcohol group, shows oxidation currents of a similar magnitude, suggesting a non‐selective oxidation process dominated by C─C bond cleavage rather than functional‐group‐specific oxidation. Interestingly, after 1 h of electrolysis of GRA, a substantial amount of formic acid was produced on the CuO catalyst, whereas only trace amounts of formic acid were detected on the Au catalyst at pH 13, and no detectable reaction occurred at pH 11.6 (Figure S22). Overall, these results indicate that CuO catalyzes a non‐selective oxidation process dominated by C─C bond cleavage irrespective of functional groups, whereas Au suppresses backbone scission and enables functional‐group‐dependent oxidation pathways.

To interpret the characteristic LSV features observed on Au (Figure 3b) from the perspective of terminal‐functional‐group oxidation selectivity, we computed the reaction energies of competing pathways illustrated in Figure 3e, with results summarized in Figure 3f (optimized structures in Table S3). On Au‐based surfaces, the aldehyde‐first oxidation pathway (leading to gluconic acid/gluconate) is energetically more favorable than the alcohol‐first oxidation pathway (leading to glucuronic acid). This suggests that initial oxidation is preferentially directed toward the aldehyde group. Notably, AuOH surface stabilizes key oxidation steps through interactions with surface hydroxyl groups, facilitating the subsequent conversion of gluconate to glucaric acid (Figure 3f). This theoretical trend aligns with our experimental finding that C6‐retaining products, such as glucaric acid, are predominantly detected at higher potentials on Au, where an OH*‐covered surface is expected (Figure 1c). Conversely, on CuO surfaces, both terminal oxidation routes are predicted to be energetically unfavorable, suggesting that other competing pathways, specifically, C─C bond cleavage, dominate the reaction landscape over terminal oxidation. A combination of model compound studies and DFT calculations confirmed that the Au and Au─OH surfaces favor aldehyde oxidation and CuO surfaces prefer C─C cleavage reaction during GLUOR.

### 2.4 GLU Ring Opening Behavior on Au and CuO Catalysts

To gain a deeper mechanistic understanding of GLUOR, it is essential to identify the molecular form (pyranose vs. open‐chain) of GLU under relevant reaction conditions. ^1^H‐ and ^13^C‐NMR analyses (Figure S23) reveal that in 0.1 M Na_2_CO_3_ solution, both GLU and arabinose predominantly exist in the pyranose ring form, whereas GNA is present in an open‐chain configuration, in good agreement with previous reports [33, 34, 35]. To further elucidate the structural characteristics of GLU adsorbed on metal catalysts, attenuated total reflection‐Fourier transform infrared (ATR‐FTIR) spectroscopy was employed (experimental details are provided in the Experimental Section). To avoid interference from the vibrational bands of carbonate species and water (Figure S24), 0.1 m NaOD/D_2_O electrolyte solution was used for IR analysis. Note that the electrochemical GLUOR behaviors of Au and CuO films on Si prism used for ATR‐FTIR experiments were similar to those of Au/C‐foam and CuO (Figure S25).

On the SiO_2_ reference surface (Figure 4a, top), GLU exhibits IR absorption at 1466 and 1390 cm^−1^, which can be assigned to OCH and CH_2_ bending vibrations [36, 37]. Moreover, the absence of a carbonyl band (typically near 1700 cm^−1^) indicates that GLU is present in a pyranose ring structure in 0.1 m NaOH solution, consistent with the solution‐phase NMR results. On the CuO surface, the IR spectrum (Figure 4a, bottom) shows absorption bands at 1447 and 1391 cm^−1^, which are similar to those observed on the SiO_2_ reference surface. This result suggests that GLU largely retains its pyranose ring structure upon adsorption on the CuO surface.

**FIGURE 4:**
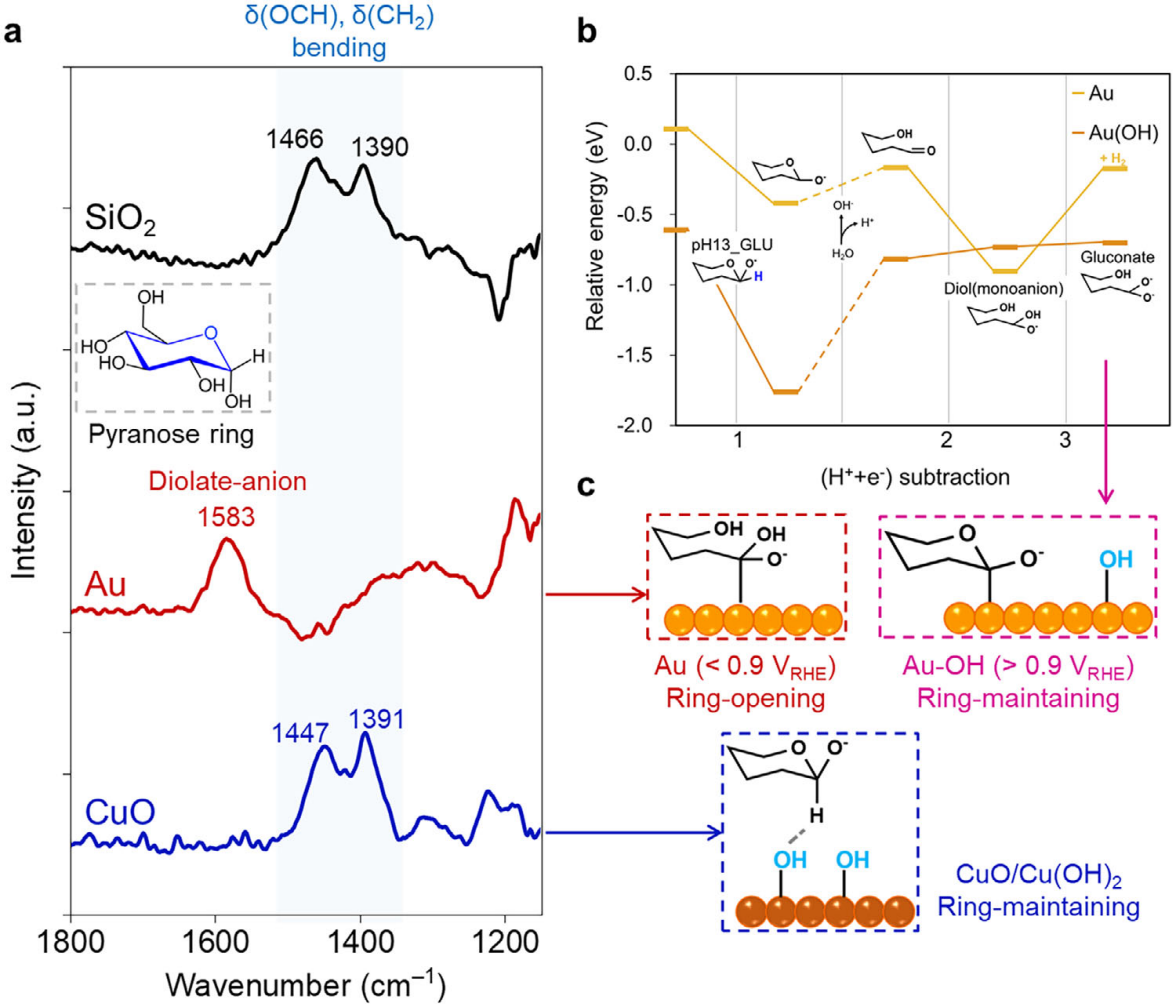
(a) ATR‐FTIR spectra measured for 100 mm GLU on different surfaces in 0.1 m NaOD/D_2_O solution. (b) Free energy diagrams constructed for GLU ring opening calculated on Au(111) and AuOH(111) surfaces. (c) Proposed adsorption configurations of GLU on Au, Au─OH, and CuO surfaces.

In contrast, on the Au surface, the characteristic bands of pyranose GLU disappear, and a new IR peak emerges at 1583 cm^−1^ (Figure 4a, middle). This band cannot be attributed to GNA formed via spontaneous oxidation of GLU on the Au surface coupled with oxygen reduction (Figure S26) [38, 39], given the discrepancy in peak position and the use of N_2_‐saturated conditions for IR measurements. Instead, the appearance of this band suggests a distinct adsorption mode of GLU on the Au surface. To further characterize the adsorbed structure of GLU intermediate on Au, IR spectra of GNA and 2‐deoxy‐glucose were also measured (Figure S27). These compounds exhibit a gradual redshift in their IR absorption bands as the electron density at the C1 position increases. Based on this trend, the bands observed in the range of 1580–1540 cm^−1^ for GLU derivatives are tentatively assigned to a diolate‐like anionic species formed at C1 of the lactol moiety, which possesses an electronic structure similar to that of a carboxylate. Interestingly, in neutral D_2_O, GLU remains in its pyranose form, indicating that deprotonation to the alkoxide form [40] is required for ring opening on the Au surface (Figure S28).

To support the structural assignment of the 1583 cm^−1^ band, the ring‐opening behavior of GLU on Au and Au─OH surfaces was compared by DFT calculations (Tables S4 and S5), because the Au─OH surface, which forms above 0.9 V, cannot be isolated experimentally under open‐circuit conditions. A reaction pathway involving initial pyranose ring opening followed by oxidation of GLU to gluconate was considered. On the Au(111) surface, the oxidative reaction following ring opening is predicted to be thermodynamically favorable, leading to the formation of gluconate through a sequence of proton‐coupled electron transfer steps along the ring‐opened pathway (Figure 4b). The most stable intermediate along this reaction coordinate is identified as a monoanionic diolate structure, which is consistent with the experimentally observed IR absorption band in Figure 4a. In contrast, on the AuOH(111) surface, deprotonated GLU is substantially stabilized, resulting in retention of the pyranose ring structure. Strong hydrogen bonding between the hydrogen atoms of GLU and surface hydroxyl groups stabilizes the cyclic configuration and suppresses pyranose ring opening. This interpretation agrees with the experimental trend that as the potential increases and the Au surface becomes more hydroxylated, the adsorption structure of glucose evolves from a ring‐opened diolate to a ring‐closed form, leading to the subsequent change in reaction pathways. Consequently, GLU preferentially undergoes oxidation to gluconolactone while maintaining its ring structure on the Au─OH surface. Collectively, these results indicate that pyranose GLU in solution undergoes ring opening on metallic Au surfaces, whereas the ring structure is preserved on hydroxylated Au and CuO surfaces.

### 2.5 In Situ Spectroscopic Identification

In situ ATR‐FTIR spectroscopy was carried out to monitor reaction intermediates formed on the catalyst surfaces during GLUOR. As shown in Figure 5a, the Au catalyst at 0.8 V_RHE_, corresponding to a predominantly metallic Au surface, exhibits absorption bands at 1599 and 1415 cm^−1^, which are assigned to the asymmetric and symmetric stretching vibrations of the carboxylate group of gluconate, respectively. Given the proposed diolate‐like adsorption of GLU on metallic Au (Figure 4a), the direct oxidation of this adsorbed species to gluconate at low potentials is plausible and consistent with the observed IR features.

**FIGURE 5:**
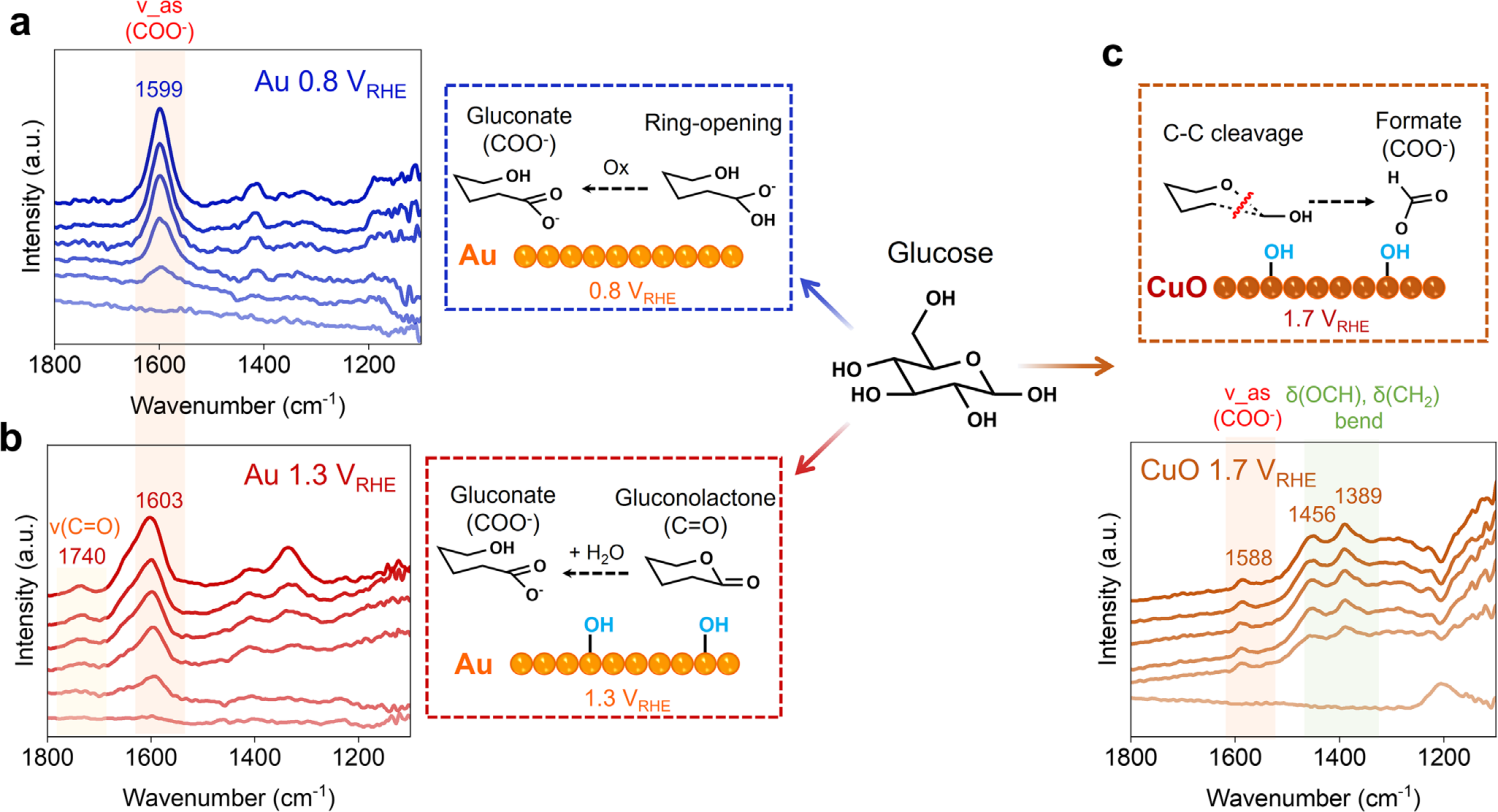
In situ IR spectra measured under constant applied potentials in 0.1 m NaOD solution containing 100 mm of GLU, and the proposed reaction mechanisms on (a) Au, (b) Au─OH, and (c) CuO surfaces.

When the applied potential is increased to 1.3 V_RHE_ (Figure 5b), where the formation of surface Au─OH species is expected, a new absorption band appears at 1740 cm^−1^. This band can be assigned to the carbonyl stretching vibration of gluconolactone (Figure S29). As predicted by the DFT calculations, the hydroxylated Au surface suppresses pyranose ring opening and instead promotes oxidation via a ring‐retained pathway, in which GLU is oxidized to gluconolactone through OH*‐assisted dehydrogenation, followed by OH^−^ (or H_2_O) attack to yield gluconate.

In contrast, the CuO catalyst at 1.7 V (Figure 5c) exhibits IR features similar to those observed under open‐circuit conditions, with absorption of GLU, indicating retention of the pyranose structure. In addition, a distinct absorption band at 1588 cm^−1^ is observed, which is assigned to the asymmetric stretching vibration of the carboxylate group of formate. Because intermediates associated with C─C bond cleavage are expected to be highly unstable, their conversion to formate likely occurs faster than the time resolution of our IR measurements (7 s). Notably, the intensity of the formate band remained nearly constant throughout the measurement rather than increasing with electrolysis time, although formate is continuously produced. This suggests that formate does not accumulate at the electrode–electrolyte interface but is rapidly desorbed and diffuses into the bulk electrolyte, establishing a steady‐state surface concentration. Such rapid product release, together with the partial dissolution of the CuO surface, may account for the difficulty in directly detecting short‐lived C─C cleavage intermediates by in situ IR analysis.

The combined experimental and theoretical results indicate that the GLUOR mechanism is governed by the catalyst identity and surface state, which dictate the adsorption configuration (ring vs open‐chain) of GLU and the preferred oxidation pathway (Figure 6). On metallic Au surfaces at relatively low potentials, deprotonated GLU in an alkoxide form undergoes dissociative C1─H adsorption [22, 23], leading to pyranose ring opening and formation of a diolate‐like open‐chain intermediate. This ring‐opened configuration enables facile aldehyde oxidation through successive proton‐coupled electron transfer steps, resulting in the selective formation of gluconate while preserving the C6 carbon backbone. C─C bond cleavage is thermodynamically disfavored on metallic Au, accounting for the high selectivity toward C6‐retaining products, especially GNA.

**FIGURE 6:**
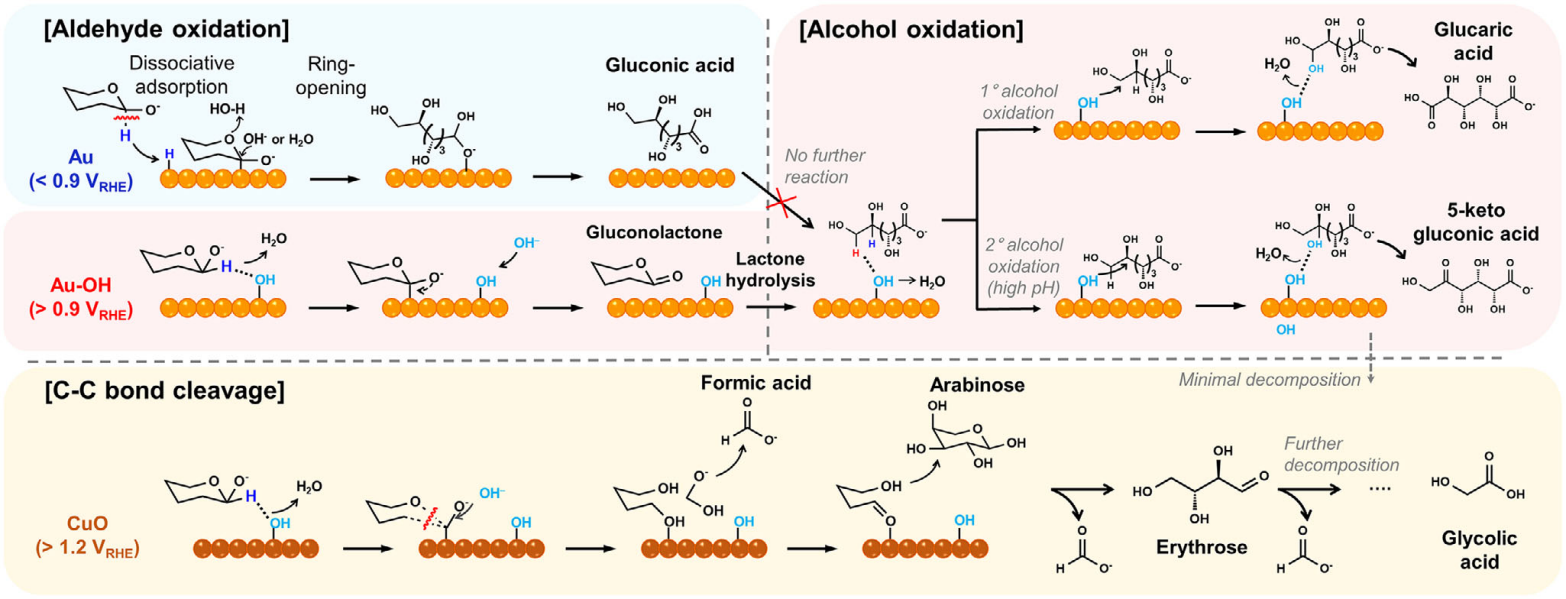
Schematic of proposed GLUOR mechanism with different applied potentials and catalysts. Proposed oxidation mechanisms on Au (top) and CuO (bottom). Secondary alcohol groups are abbreviated for clarity.

At higher anodic potentials, the Au surface becomes hydroxylated, which substantially alters the reaction pathway. On Au─OH surfaces, strong hydrogen bonding between surface OH groups and GLU stabilizes the pyranose ring structure and suppresses ring opening. Under these conditions, GLU undergoes oxidation via a ring‐retained pathway, forming gluconolactone as a key intermediate, which is subsequently converted to gluconate through OH^−^ (or H_2_O) attack. The presence of surface hydroxyl species is also proposed to facilitate alcohol oxidation, enabling further oxidation to GRA and other alcohol‐oxidized products at elevated potentials, while C─C bond cleavage remains a minor pathway.

In contrast, CuO surfaces exhibit a fundamentally different reaction landscape. GLU adsorbs on CuO predominantly in its pyranose form, with functional‐group oxidation pathways being energetically unfavorable. Instead, CuO intrinsically favors C─C bond cleavage, which proceeds downhill in free energy, leading to rapid fragmentation of the carbon backbone. As a result, GLU is decomposed into smaller C1–C5 species, with formate emerging as the dominant final product.

## 3 Conclusion

In conclusion, this study comprehensively elucidates how catalyst surface states and the adsorption configuration of GLU dictate electrocatalytic GLUOR pathways and product selectivity. Au and CuO catalysts show clearly different product distributions. On Au catalysts, GNA is selectively produced at low potentials via aldehyde oxidation, whereas alcohol oxidation also becomes accessible at higher potentials, leading to the formation of GRA and 5‐keto‐gluconic acid with only minor amounts of C─C cleavage products. In contrast, CuO catalysts predominantly promote C─C bond cleavage regardless of applied potential and electrolyte pH, with ≤C5 species as the major products. By combining electrochemical measurements with theoretical calculations and in situ spectroscopic analyses, the underlying reaction mechanisms were further analyzed. Metallic Au surfaces promote dissociative adsorption and pyranose ring opening of GLU under alkaline conditions, enabling selective aldehyde oxidation to gluconate while preserving the C6 carbon backbone. Upon surface hydroxylation at higher potentials, Au─OH suppresses ring opening and leads to oxidation to gluconolactone, accompanied by activation of alcohol oxidation pathways. In contrast, CuO surfaces intrinsically favor C─C bond cleavage, resulting in non‐selective fragmentation into smaller carbon products. This work establishes a unified mechanistic framework linking surface‐dependent adsorption structures to GLUOR selectivity and provides fundamental guidelines for the rational design of electrocatalysts for selective carbohydrate valorization.

## 4 Experimental Section

### 4.1 Chemicals and Materials

D‐glucose (≥99.5%), D‐gluconic acid sodium salt (≥99%), D‐glucuronic acid (≥98%), 5‐keto‐D‐gluconic acid (≥98%), D‐saccharic acid potassium salt (≥98%), 2‐deoxy‐D‐glucose (≥98%), L‐arabinose (≥99%), D‐erythrose (≥75%), D‐glyceric acid sodium salt (≥95%), dihydroxyacetone (pharmaceutical secondary standard), glycolic acid (≥99%), formic acid (≥95%), gold(III) chloride trihydrate (≥99.9%), Cu(II) oxide (nanopowder, <50 nm particle size), sodium deuteroxide solution (40 wt% in D_2_O), Nafion, deuterium oxide (99.9 atom% D), deuterium oxide (99.9 atom% D, contains 0.05 wt. % 3‐(trimethylsilyl)propionic‐2,2,3,3‐d_4_ acid, sodium salt), sulfuric acid (99.9%), acetonitrile (99.9%) were purchased from Merck. Sodium hydroxide (≥97%), sodium carbonate (≥99%), and hydrochloric acid (35%) were purchased from DUKSAN. Glassy carbon foam (Goodfellow, 3.2 mm thick, porosity 96.5%), copper foam (MTI, 99.9%), deionized water (DI water, 18.2 MΩ cm, Smart2Pure 6 UV/UF, ThermoFisher) were used. Rotating disk electrode tips (glassy carbon disk, AFE5T050GC and gold disk, AFE5T050AU) were purchased from Pine Research.

### 4.2 Electrode Preparation and Characterization

Au/carbon foam (Au/C‐foam) electrode was prepared by electrodeposition [41]. The electrodeposition bath was prepared with 5 mm HAuCl_4_ and 0.1 m NaNO_3_ and the pH was adjusted to 6.3 with 3 m NaOH. Carbon foam (2 cm x 1 cm) was cleaned with ethanol and water several times and dried at room temperature. Carbon foam was immersed in the deposition bath, and a constant potential of 0.8 V was applied until a charge of 10 C was passed. Before electrochemical measurements, all Au/C‐foam and Cu foam electrodes were immersed in 1 m H_2_SO_4_ and washed with water and ethanol several times. All electrodes were activated by cyclic voltammetry (CV) between 0 and 1.7 V vs RHE in the same electrolyte used for electrolysis for five cycles at a scan rate of 10 mV s^−1^. The electrolyte was then replaced with a fresh solution before the oxidation of glucose.

Surface morphology was examined by field emission SEM (Hitachi, Regulus8230, Japan). The XRD patterns of electrodes were obtained using an X‐ray diffractometer (D/max2500/PC, Rigaku, Japan) with a Cu‐Kα radiation source at 18 kW (60 kV, 300 mA). Oxidation states of the electrode surface were investigated with an X‐ray photoelectron spectrometer (PHI 5000 VersaProbe, Ulvac‐PHI, Japan) using Al Kα (1486.6 eV). The binding energy was calibrated against the C 1s peak at 284.6 eV. The amounts of Cu and Au leached into the electrolyte after 24 h glucose electrolysis were determined using ICP‐OES (iCAP 7000, Thermo Fisher Scientific), as summarized in Table S6.

### 4.3 Electrochemical Measurements

All electrochemical measurements were conducted using a potentiostat (SP‐240, BioLogic) in an H‐type cell, separated by a Nafion 117 membrane with vigorous stirring (700 rpm). 0.1 m Na_2_CO_3_ or 0.1 m NaOH solution (15 mL for each chamber of H‐type cell) was used as the electrolyte. 10 mm of GLU was added into the anolyte unless noted otherwise. Au/C‐foam and Cu foam were used as the working electrode, and a Pt mesh was used as the counter electrode. Hg/HgO (for alkaline media) or Ag/AgCl (for neutral media) was employed as the reference electrode.

All measured potentials were converted to the RHE scale as follows:

$$\;E_{\mathrm{RHE}}=E_{\mathrm{measured}}+0.05916\;\times \mathrm{pH}+\left(E_{\mathrm{Hg}/\mathrm{HgO}}\;or\;E_{\mathrm{Ag}/\mathrm{AgCl}}\right)$$

The pH was measured with a pH meter (Mettler‐Toledo S220), and *E*_Hg/HgO_ and *E*_Ag/AgCl_ are 0.082 and 0.241 V.

LSV and CV measurements were performed at a scan rate of 10 mV s^−1^. Electrochemical conversion experiments for product analysis were conducted under constant‐potential electrolysis conditions.

The GLUOR products were evaluated after conducting pulsed chronoamperometry (CA) measurements for Au/C‐foam [42].

For rotating disk electrode (RDE) measurements, an Au disk electrode and a CuO‐coated disk electrode were used. The CuO‐coated disk electrode was prepared by drop‐casting a catalyst ink onto a glassy carbon disk electrode. Prior to catalyst deposition, the glassy carbon disk electrode was polished and subsequently rinsed with ethanol and deionized water. The catalyst ink was prepared by sonicating a suspension containing CuO nanopowder (10 mg) and Nafion solution (10 µL) in 1 mL of ethanol. The resulting ink was drop‐cast onto the disk electrode, corresponding to a total CuO loading of 1 mg. LSV measurements were conducted at a rotation speed of 1600 rpm.

The ECSA of CuO foam was estimated from C_dl_ obtained by CV in the non‐Faradaic region (scan rates of 25–200 mV s^−1^) using a specific capacitance of 130 µF cm^−2^ [43], and that of Au/C‐foam was determined from the Au oxide reduction charge assuming 390 µC cm^−2^ [44] (Figure S4).

### 4.4 Product Analysis

The ≤C5 products were analyzed by HPLC equipped with a refractive index detector (YL 9100) using a Bio‐Rad Aminex HPX‐87H column (Figure S30a). A 5 mm H_2_SO_4_ aqueous solution was used as the mobile phase at a flow rate of 0.5 mL min^−1^. The electrolyte sample (100 µL) was diluted with 5 mm H_2_SO_4_ (1.5 mL), and an aliquot of 20 µL was injected into the column. GLU, C6 products, and tartronic acid were analyzed by HPLC equipped with an evaporative light scattering detector (Agilent 1260 infinity II) and a Poroshell 120 HILIC‐Z column (Figure S30b). A mixed mobile phase consisting of 100 mm ammonium formate (pH adjusted to 9) and acetonitrile was employed with two gradient compositions (50:950 and 400:600, v:v) at a flow rate of 0.4 mL min^−1^. The electrolyte sample (90 µL) was acidified by mixing with 1 m HCl (10 µL). A 2 µL aliquot of the resulting solution was injected into the column. Calibration curves for ≤C5 products and GLU, C6 products and tartronic acid were obtained from a series of standard solutions (Figures S31 and S32).

The selectivity and Faradaic efficiency (FE) of each product were calculated according to the following equations:

$$\mathrm{FE}\;\left(\%\right)=\frac{n_{\mathrm{product}}\times N_{e}\times F}{Q}\;\times 100$$

$$\mathrm{Selectivity}\;\left(\%\right)=\frac{n_{\mathrm{product}}\times N_{\mathrm{carbon}}}{n_{\mathrm{GLU},\mathrm{converted}}\times 6}\;\times 100$$

where *n*_product_ is the number of moles of a given product, *n*_GLU_ is the initial amount of GLU (mol), *n*_GLU, converted_ is the number of moles of converted GLU, *N*_carbon_ is the number of carbon atoms in the given product, *N*_e_ is the number of electrons transferred during the reaction forming the product, *F* is the Faraday constant (96485 C mol^−1^), and *Q* is the total charge passed (C).

GLU structural analysis was performed using a 400 MHz ^1^H and ^13^C nuclear magnetic resonance (NMR) spectrometer (Agilent, USA). Samples with a concentration of 0.1 m were prepared by dissolving the corresponding compounds in 0.1 m Na_2_CO_3_ solution. For NMR measurements, 400 µL of the sample solution was transferred to an NMR tube and mixed with 100 µL of D_2_O containing 0.75% (v/v) 3‐(trimethylsilyl)propionic‐2,2,3,3‐d4 acid, sodium salt as an internal standard.

### 4.5 Spontaneous Reaction

Spontaneous reactions were conducted in a beaker cell containing an aqueous solution of 0.1 m NaOH or 0.1 m Na_2_CO_3_ with 10 mm GLU. Prior to the reaction, the electrode was electrochemically activated through CV in the corresponding electrolyte solution. GLU was added into the solution after activation, and the reaction was allowed to proceed for 24 h under open‐circuit conditions. Blank experiments were performed under identical conditions in the absence of electrodes.

### 4.6 In Situ IR Analysis

In situ attenuated total reflection‐Fourier transform infrared (ATR‐FTIR) spectroscopy was performed using a Bruker VERTEX 80v Fourier transform infrared spectrometer equipped with a liquid‐nitrogen‐cooled mercury cadmium telluride (MCT, HgCdTe) detector and a variable‐angle specular reflectance accessory (VeeMAX III, Pike Technologies). A silicon prism beveled at 60° was used as the ATR window.

Catalysts were deposited onto the Si prism to serve as the working electrode, while a platinum wire and an Ag/AgCl electrode were used as the counter and reference electrodes, respectively. Infrared spectra were obtained by averaging 10 interferograms collected at a spectral resolution of 4 cm^−1^. The interferograms were recorded under constant applied potential with a time window of 7 s.

Baseline spectra were collected using the base electrolyte prior to each ATR‐FTIR experiment, and all spectra were referenced accordingly.

Reference spectra of glucose, gluconic acid, gluconolactone and sodium formate were measured on the SiO_2_ prism in D_2_O (Figure S29).

### 4.7 In Situ Raman Analysis

In situ Raman spectra were acquired using a Raman microscope (inVia, Renishaw), equipped with a 785 nm diode laser and a 1200 lines mm^−1^ grating. The laser was focused onto the electrode surface through a ×50 objective. The spectrometer was calibrated using the 520.7 cm^−1^ band of a Si wafer. An Au foil was used as the working electrode in 0.1 m NaOH. Spectra were collected at open‐circuit potential (OCP), 1.1 and 1.3 V_RHE_.

### 4.8 DFT Calculations

Density functional theory (DFT) calculations were performed using the Vienna Ab initio Simulation Package (VASP) [45, 46, 47, 48]. The ion‐electron interactions were described using the projector augmented‐wave (PAW) method [49, 50] and the exchange‐correlation functional was treated with the vdW‐DF2 functional to account for nonlocal van der Waals interactions for all reaction intermediates [51]. A plane‐wave cutoff energy of 450 eV was used. All structures were optimized until the total energy and residual forces on each atom were converged to within 1×10^−4^ eV and 1×10^−4^ eV Å^−1^, respectively. Structural optimizations were carried out using the conjugate‐gradient algorithm.

To represent the alkaline electrochemical environment, the reaction energetics were modeled using the computational hydrogen electrode method [52]. In this study, OH^−^ ions were applied as the reactive species to reflect the basic experimental conditions. The chemical potential of the OH^−^ was related to the chemical potentials of H_2_O and H_2_ through the equilibrium H_2_O + e^−^ ↔ 1/2H_2_ + OH^−^, where the potential is referenced to the reversible hydrogen electrode (RHE).

For bulk Au and CuO, both lattice parameters and atomic positions were optimized, whereas for surface slab calculations the cell shape and volume were fixed and only atomic positions were relaxed. The optimized lattice parameters were *a* = *b* = *c* = 4.18 Å for cubic Au and *a* = 4.27 Å, *b* = 3.86 Å, and *c* = 5.13 Å for monoclinic CuO. The Au(111) and CuO(111) surfaces constructed from these optimized bulk structures were used as surface models. Brillouin‐zone sampling was performed using gamma‐centered grids, with 16 × 16 × 16 k‐points for bulk calculations and 4 × 4 × 1 for surface slabs. For CuO(111), DFT+U was applied with *U*_eff_ = 7 eV based on previous reports [53]. To determine the thermodynamically stable surface states under experimental conditions, surface Pourbaix diagrams were constructed for each surface (Figure S33). Based on these diagrams, the Au(OH)(111) and CuO(111) models were constructed with adsorbed OH coverages of 0.25 and 0.5 ML, respectively, reflecting the stable configurations at the investigated potential range.

## Author Contributions

**Myeonghwan Oh**: conceptualization, investigation, writing – original draft, writing – review and editing, validation, visualization, methodology, formal analysis, data curation, software. **Hoeun Seong**: data curation, conceptualization, investigation, writing – original draft, writing – review and editing, visualization, validation, methodology, formal analysis. **Gaeun Jo**: conceptualization, investigation, writing – original draft, software, formal analysis, visualization, validation, methodology, writing – review and editing, data curation. **Jiyoung Hwang**: investigation, writing – original draft, writing – review and editing, visualization, validation, formal analysis, methodology. **Yurim Sohn**: investigation, writing – original draft, writing – review and editing, visualization, formal analysis, data curation, methodology. **Kyoungsuk Jin**: investigation, writing – original draft, funding acquisition, project administration, supervision, data curation, writing – review and editing, validation. **Jong Hyeok Park**: investigation, writing – original draft, writing – review and editing, validation, supervision. **Wooyul Kim**: investigation, writing – original draft, writing – review and editing, validation, formal analysis, supervision, data curation, methodology. **Seok Ki Kim**: conceptualization, investigation, funding acquisition, writing – original draft, writing – review and editing, visualization, validation, methodology, software, formal analysis, project administration, resources, supervision, data curation. **Dong Ki Lee**: conceptualization, investigation, funding acquisition, writing – original draft, writing – review and editing, visualization, validation, methodology, software, formal analysis, project administration, resources, supervision, data curation.

## Conflicts of Interest

The authors declare no conflicts of interest.

## Supporting information




**Supporting File**: advs78627‐sup‐0001‐SuppMat.pdf.

## Data Availability

The data that supports the findings of this study are available in the Supporting Information of this article.
