# Supplementary material for: Surface‐State‐Driven Selectivity of Electrocatalytic Glucose Oxidation: Metallic Au, Au─OH, and CuO
Source: Adv Sci (Weinh). 2026 Nov 5:e78627. Online ahead of print. doi: 10.1002/advs.78627 (PMC13735442; doi:10.1002/advs.78627)
Supplement: Supplementary file 1 [file ADVS-9999-e78627-s001.pdf]

Supporting Information for

Surface-State-Driven Selectivity of Electrocatalytic Glucose Oxidation: Metallic Au, Au-OH and CuO

Myeonghwan Oh,[a,b,1] Hoeun Seong,[a,1] Gaeun Jo,[c,1] Jiyoung Hwang,[c] Yurim Sohn,[d] Kyoungsuk Jin,[b] Jong Hyeok Park,[e] Wooyul Kim,[d] Seok Ki Kim,[c] * Dong Ki Lee[a,e,f] *

[a]Clean Energy Research Center, Korea Institute of Science and Technology (KIST), Seoul 02792, Republic of Korea
[b]Department of Chemistry, Korea University, Seoul 02841, Republic of Korea
[c]Department of Chemical Engineering, Ajou University, Suwon 16499, Republic of Korea
[d]Department of Energy Engineering, Korea Institute of Energy Technology, Naju 58330, Republic of Korea
[e]Department of Chemical and Biomolecular Engineering, Yonsei-KIST Convergence Research Institute, Yonsei University, Seoul 03722, Republic of Korea
[f]Graduate School of Energy and Environment, Korea University, Seoul 02841, Republic of Korea

[1]These authors contributed equally: Myeonghwan Oh, Hoeun Seong, Gaeun Jo

*Corresponding: S.K.K. seokki@ajou.ac.kr, D.K.L. dnklee@kist.re.kr

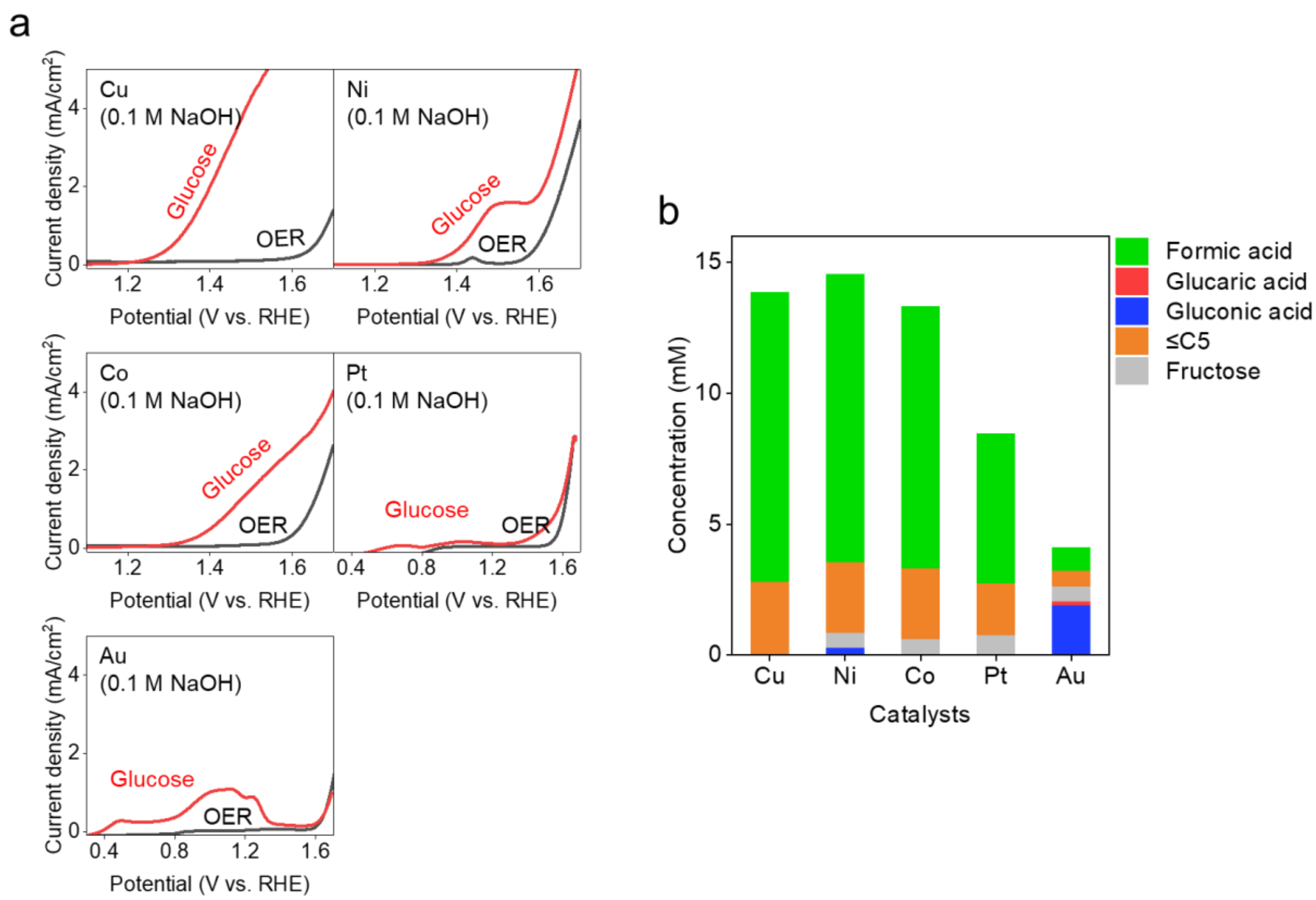

Figure S1. Screening of catalysts for GLUOR in 0.1 M NaOH. (a) LSV recorded at 10 mV s^{-1} on different metal foils (Cu, Ni, Co, Pt and Au). (b) Product distribution at selected potentials after 40 C passed on metal foils. The initial glucose concentration was 5 mM.

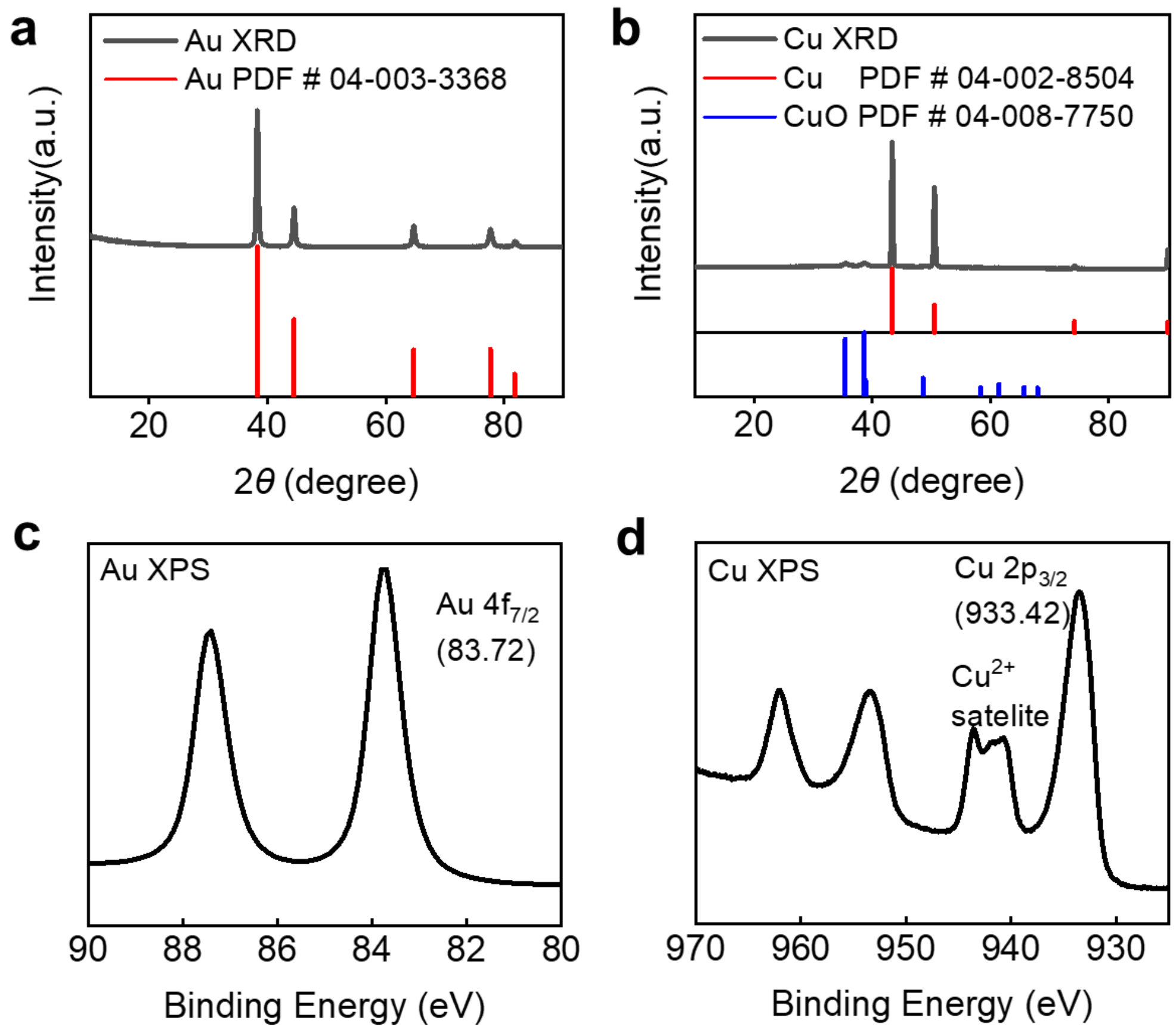

Figure S2. X-ray diffraction patterns and XPS spectra. (a,b) XRD patterns for Au and Cu foil. (c,d) XPS spectra of Au 4f and Cu 2p. All measurements were conducted after electrochemical activation in 1 M NaOH solution.

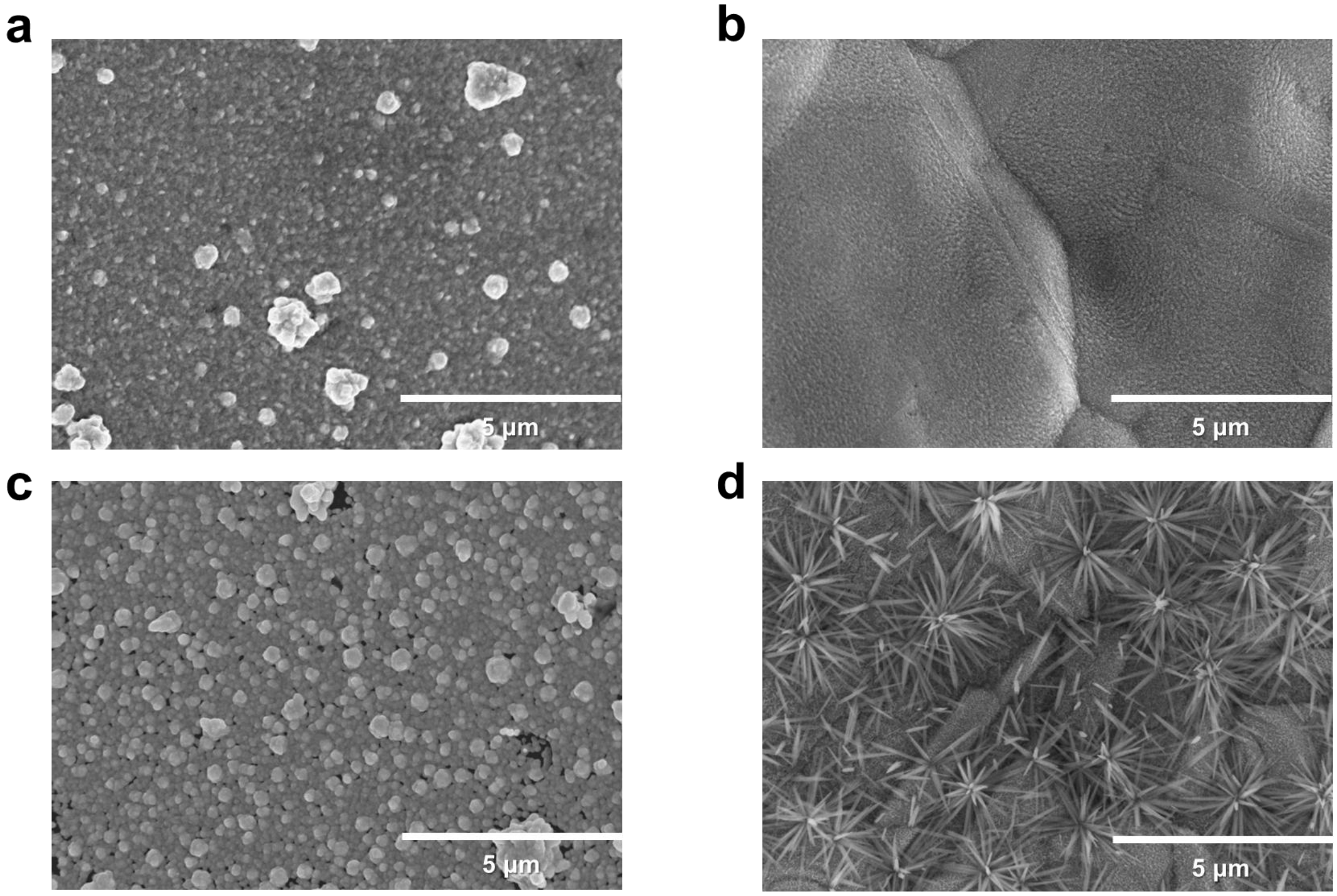

Figure S3. SEM images of Au/C-foam and Cu foam. SEM images of Au/C-foam (a,c) and Cu foam (b,d) after activation in 0.1 M Na_2CO_3 (a,b) and 0.1 M NaOH (c,d).

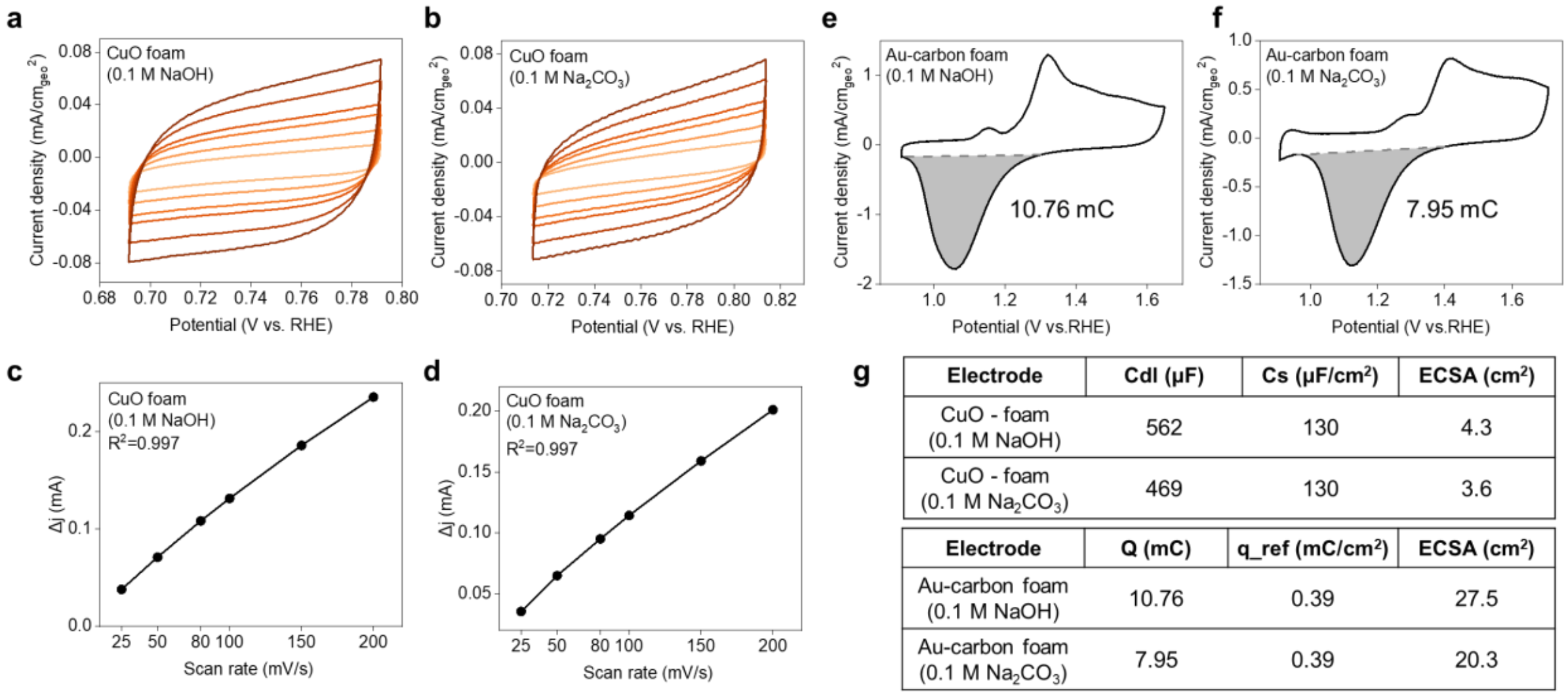

Electrode	Cdl (μF)	Cs (μF/cm²)	ECSA (cm²)
CuO - foam (0.1 M NaOH)	562	130	4.3
CuO - foam (0.1 M Na_2CO_3)	469	130	3.6

Electrode	Q (mC)	q_ref (mC/cm²)	ECSA (cm²)
Au-carbon foam (0.1 M NaOH)	10.76	0.39	27.5
Au-carbon foam (0.1 M Na_2CO_3)	7.95	0.39	20.3

Figure S4. Determination of the electrochemically active surface area (ECSA). (a,b) CV curves of CuO foam recorded at scan rates of 25-200 mV s^{-1} in (a) 0.1 M NaOH and (b) 0.1 M Na_2CO_3. (c,d) Capacitive current (Δj) plotted against the scan rate in (c) 0.1 M NaOH and (d) 0.1 M Na_2CO_3; the slope gives the double-layer capacitance (C_{dl}). (e,f) CV curves of Au/C-foam at a scan rate of 100 mV s^{-1} in (e) 0.1 M NaOH and (f) 0.1 M Na_2CO_3; the shaded area represents the charge of the Au oxide reduction peak. (g) Summary of C_{dl}, the specific capacitance (C_s = 130 μF cm^{-2}), the Au oxide reduction charge (Q, q_{ref} = 390 μC cm^{-2}) and the resulting ECSA values.

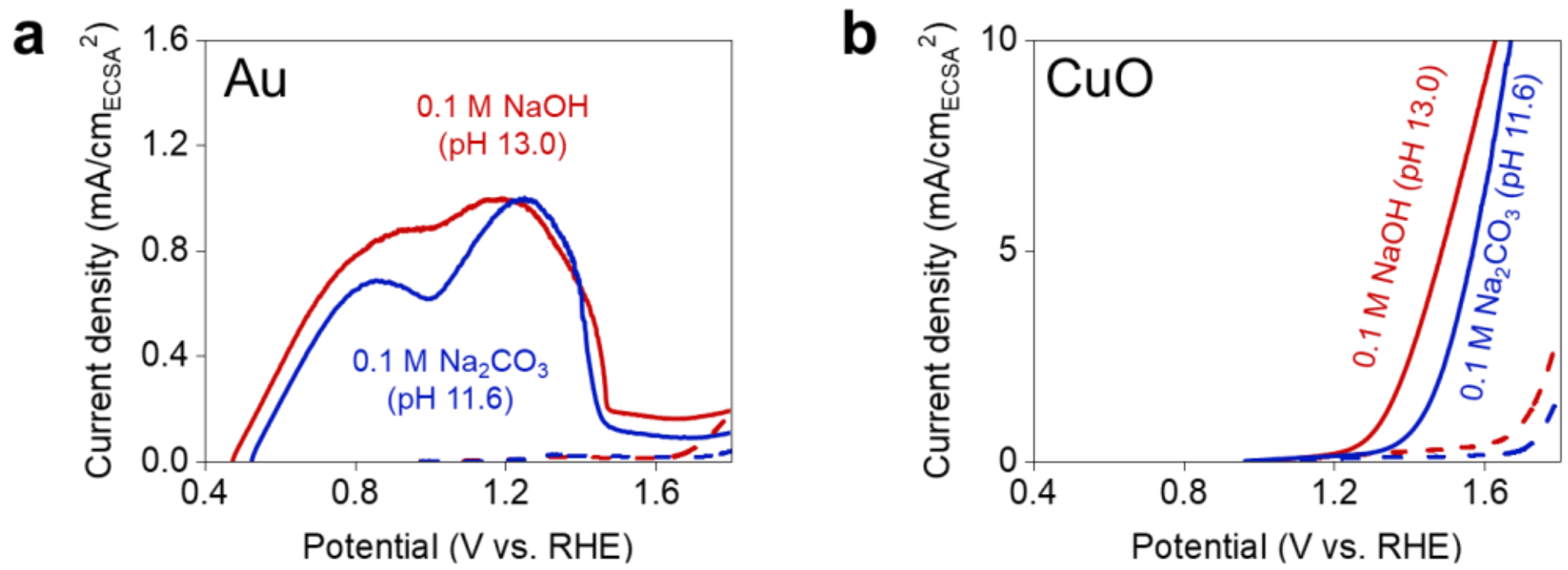

Figure S5. ECSA-normalized LSV curves. LSV curves of (a) Au/C-foam and (b) CuO recorded at 10 mV s^{-1} with 10 mM GLU in 0.1 M NaOH (pH 13.0) and 0.1 M Na_2CO_3 (pH 11.6), normalized by the ECSA values in Figure S4. The traces without GLU are shown as dashed lines.

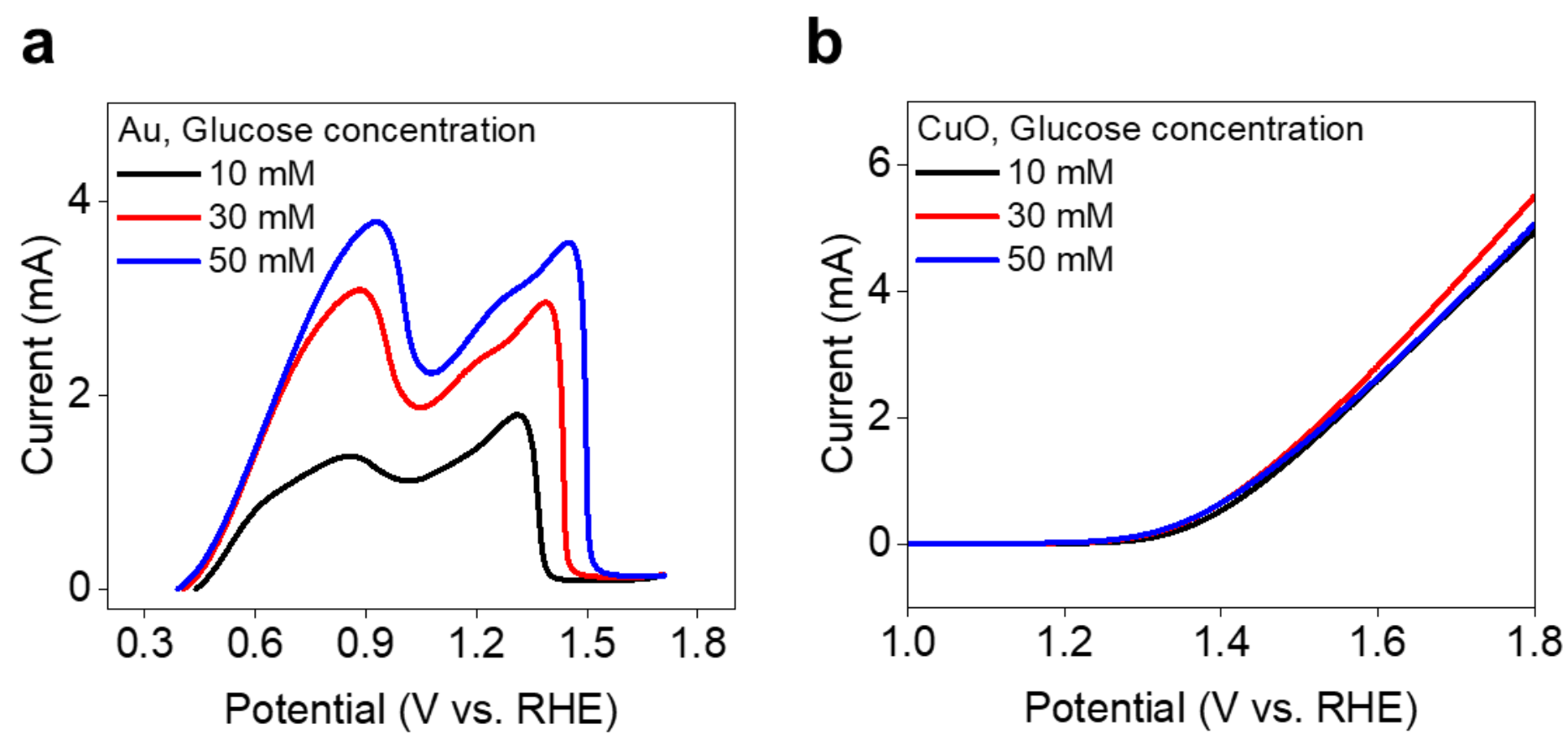

Figure S6. Effect of GLU concentration. LSV curves recorded at 10 mV s^{-1} on (a) the Au RDE and (b) the CuO-coated RDE in 0.1 M NaOH containing 10, 30 and 50 mM GLU at 1600 rpm. On Au, the current increases and the current drop shifts to more positive potentials with increasing GLU concentration, whereas the curves on CuO nearly overlap.

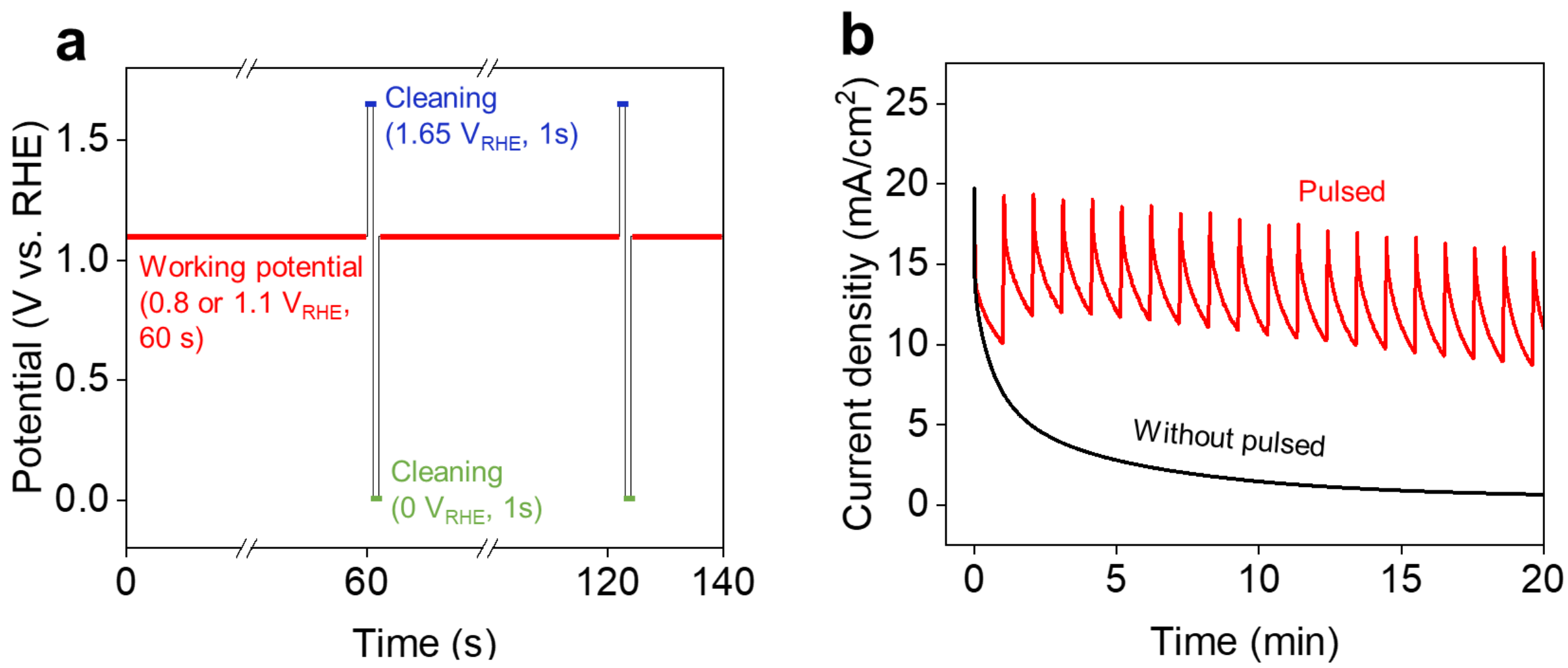

Figure S7. Pulsed chronoamperometric procedure. (a) Pulsed chronoamperometry protocol. (b) Comparison of current density measured with and without pulsed chronoamperometry at 1.1 V_{RHE}. We selected 0 and 1.65 V_{RHE}, where GLUOR does not occur. This protocol was applied only to the Au/C-foam electrode; the oxidation on Cu foam was conducted without pulsed chronoamperometry.

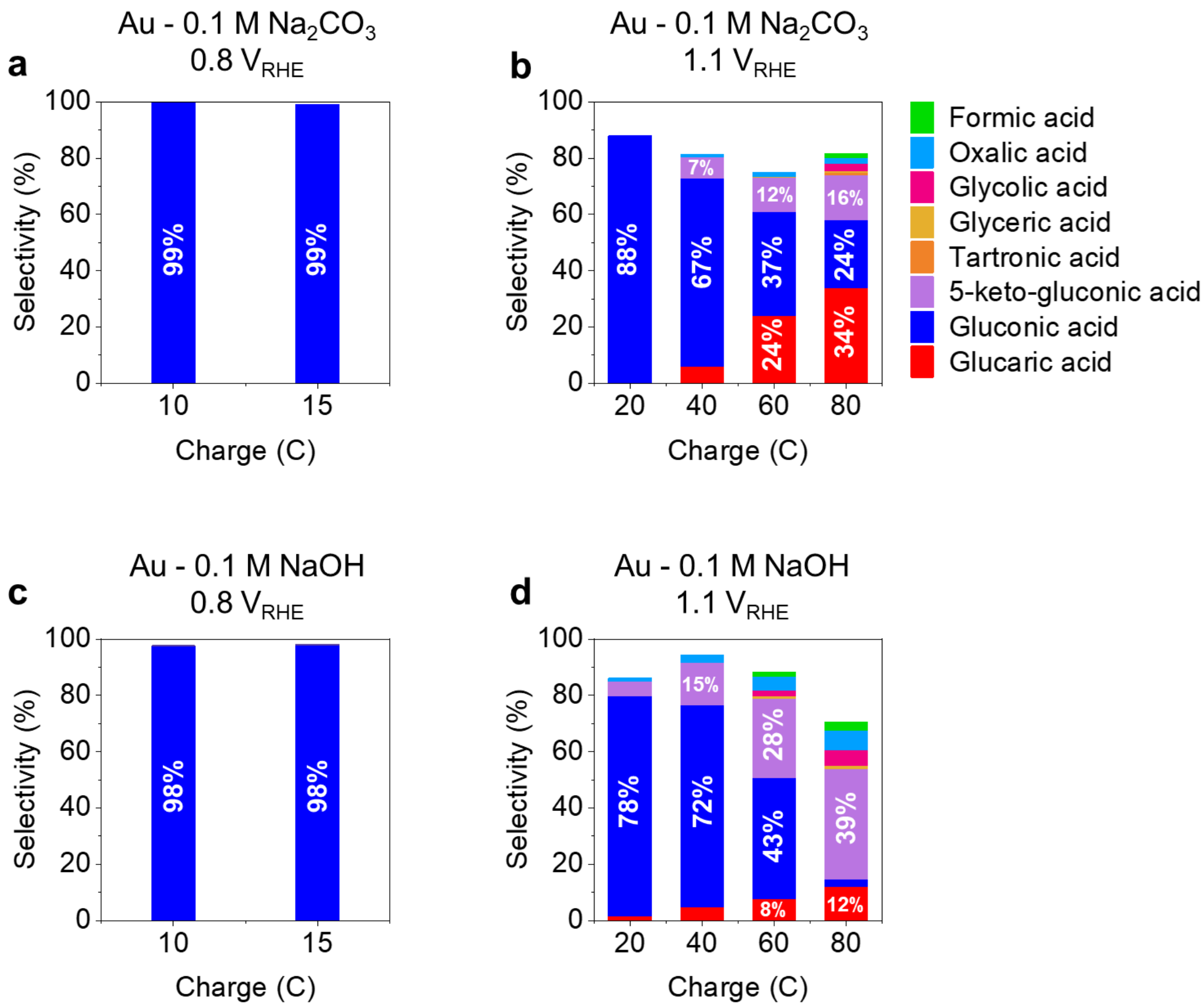

Figure S8. Product selectivity after GLUOR on Au/C-foam. Electrolysis was conducted using pulsed chronoamperometry at 0.8 V_{RHE} (a,c) and 1.1 V_{RHE} (b,d) in 0.1 M Na_2CO_3 (a,b) and 0.1 M NaOH (c,d).

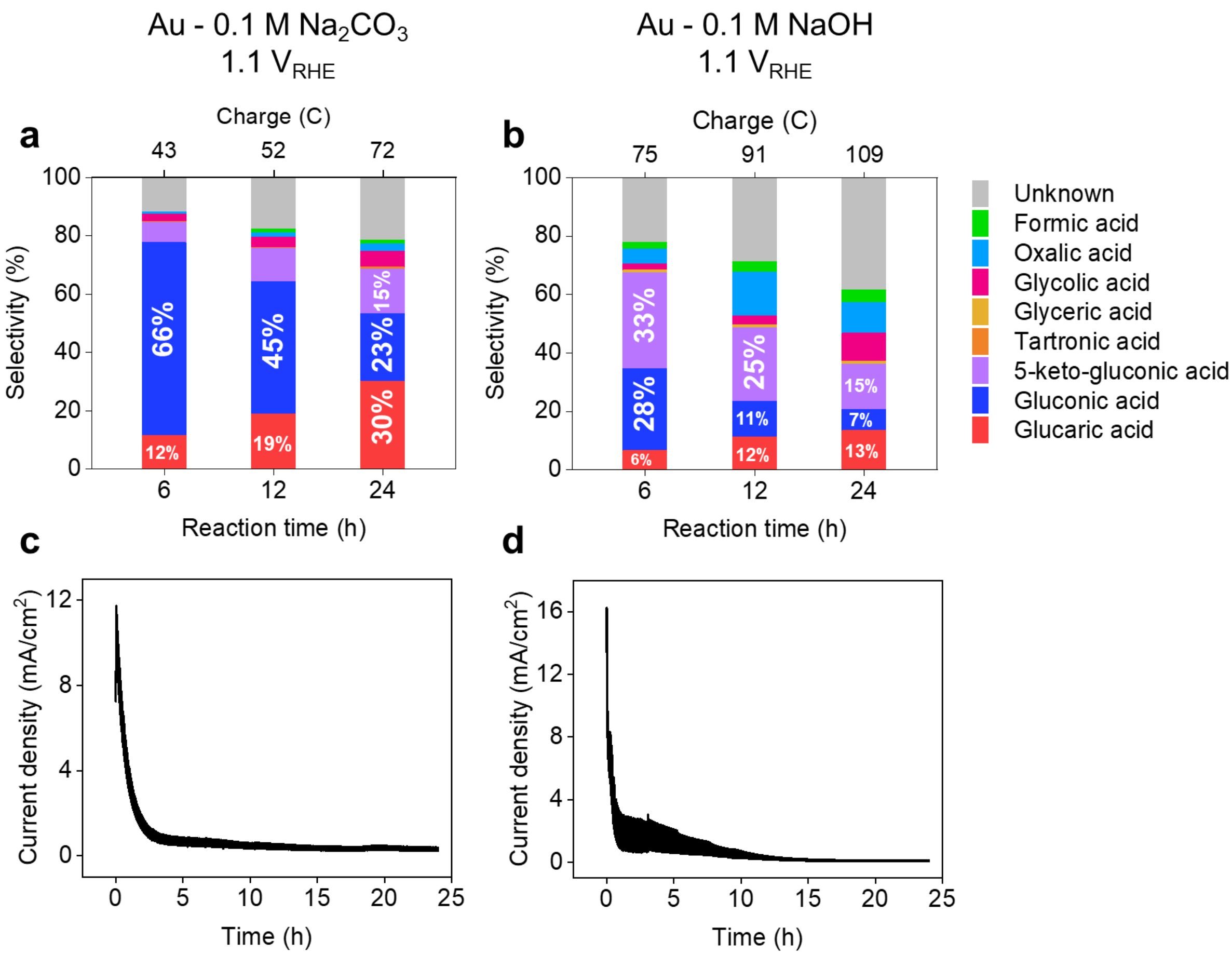

Figure S9. Time-dependent product selectivity of GLUOR on Au/C-foam at 1.1 V_{RHE} during 24 h electrolysis. Product selectivity in (a) 0.1 M Na_2CO_3 and (b) 0.1 M NaOH, and the corresponding *j-t* curves in (c) 0.1 M Na_2CO_3 and (d) 0.1 M NaOH.

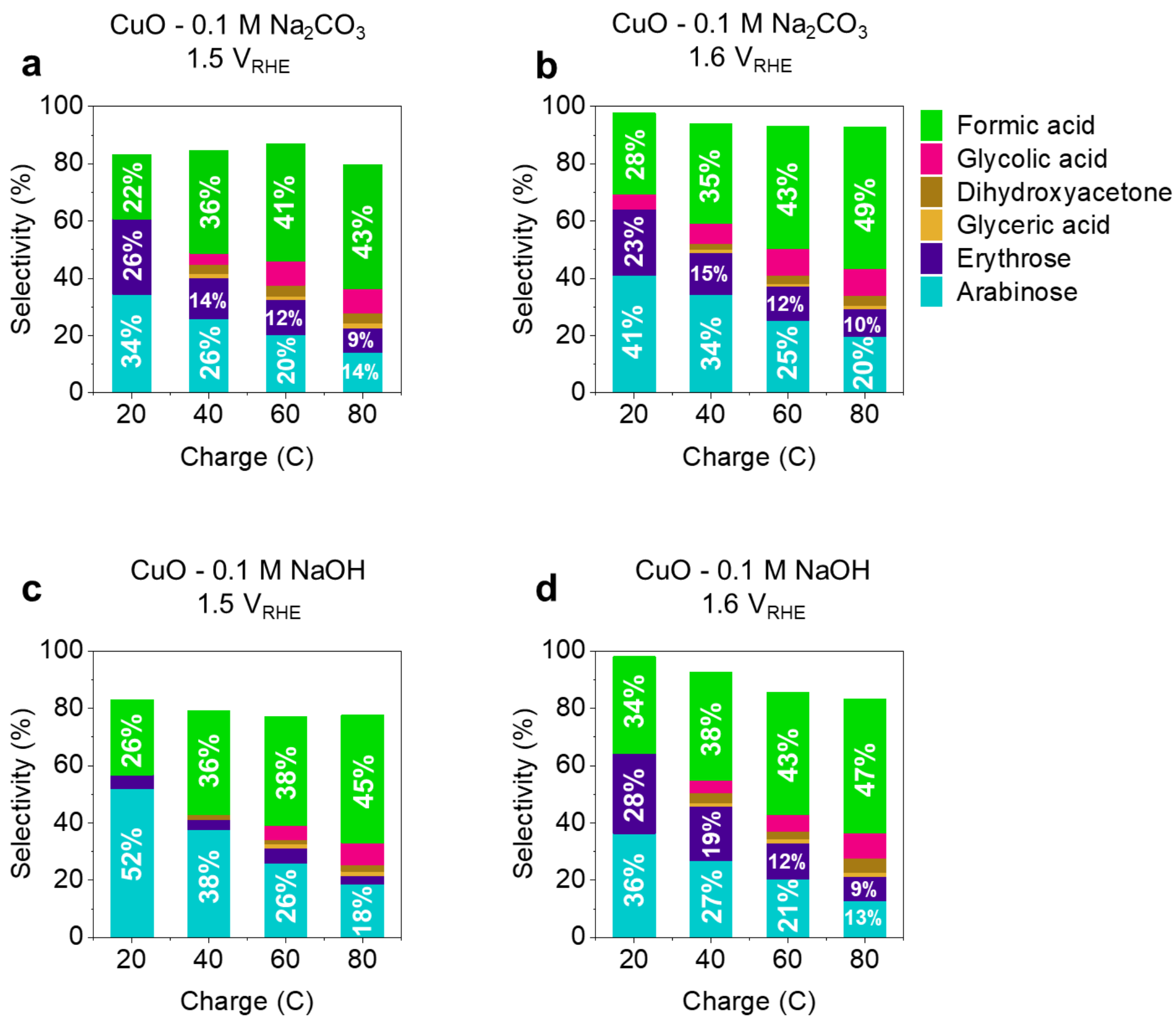

Figure S10. Product selectivity after GLUOR on CuO. Electrolysis was conducted using constant potential at 1.5 V_{RHE} (a,c) and 1.6 V_{RHE} (b,d) in 0.1 M Na_2CO_3 (a,b) and 0.1 M NaOH (c,d).

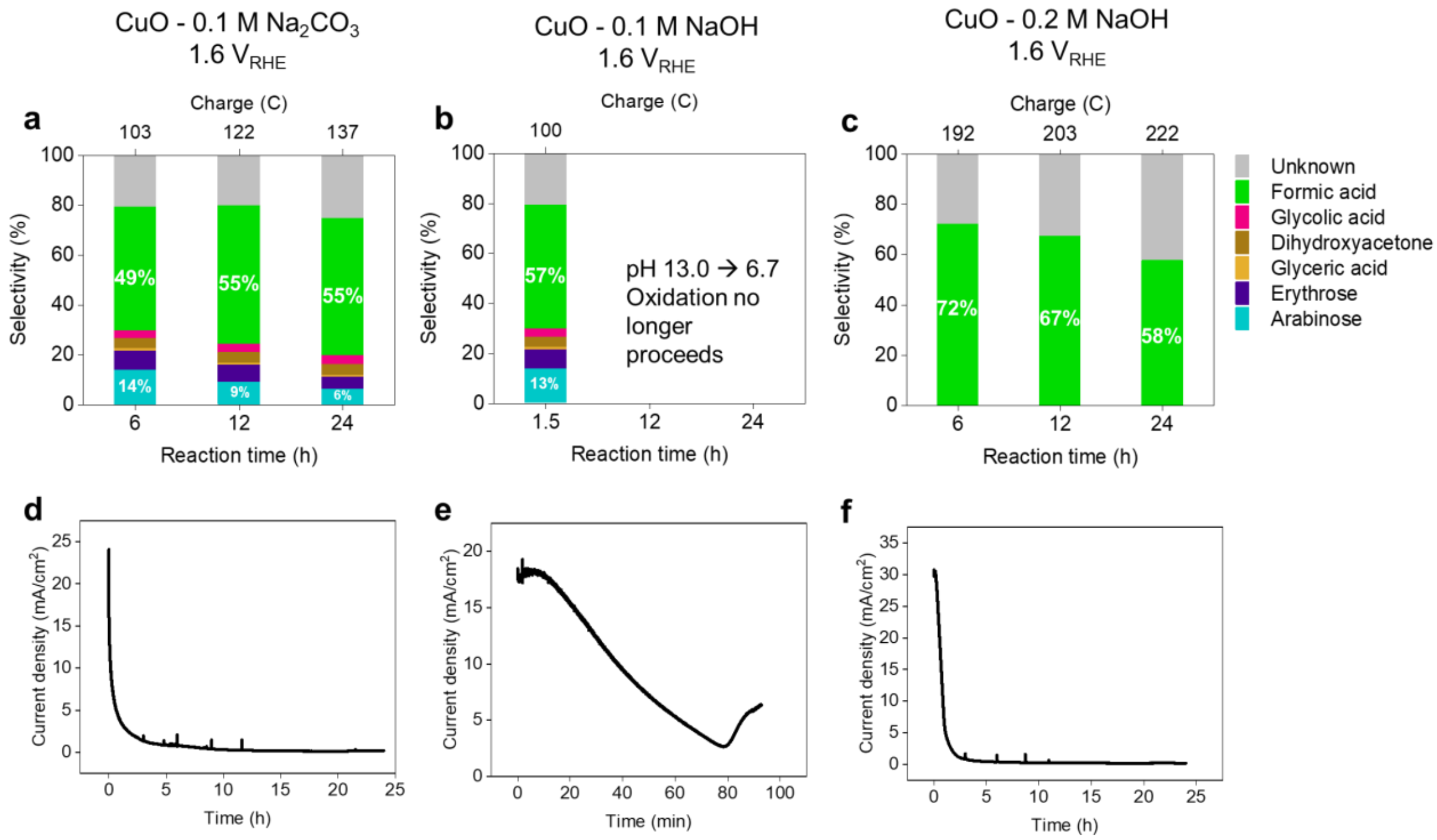

Figure S11. Time-dependent product selectivity of GLUOR on CuO at 1.6 V_{RHE}. Product selectivity in (a) 0.1 M Na_2CO_3, (b) 0.1 M NaOH and (c) 0.2 M NaOH, and (d-f) the corresponding *j-t* curves. In 0.1 M NaOH, the pH decreased from 13.0 to 6.7 after electrolysis, and the oxidation reaction no longer proceeded under these conditions. Therefore, 0.2 M NaOH was further examined to investigate the deep oxidation of GLU.

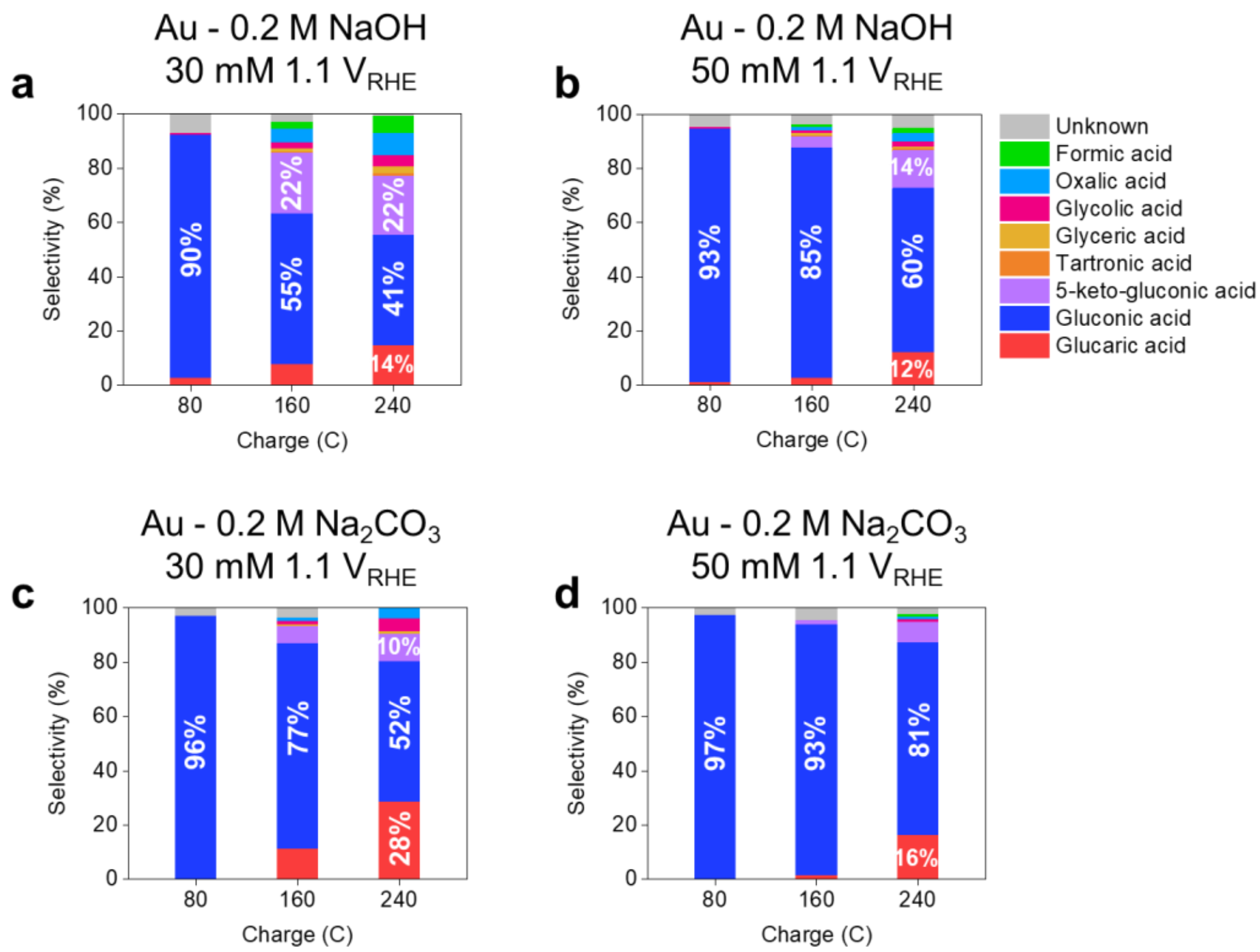

Figure S12. Product selectivity of GLUOR on Au/C-foam at higher GLU concentrations. Electrolysis was conducted at 1.1 V_{RHE} with (a) 30 mM and (b) 50 mM GLU in 0.2 M NaOH, and (c) 30 mM and (d) 50 mM GLU in 0.2 M Na_2CO_3.

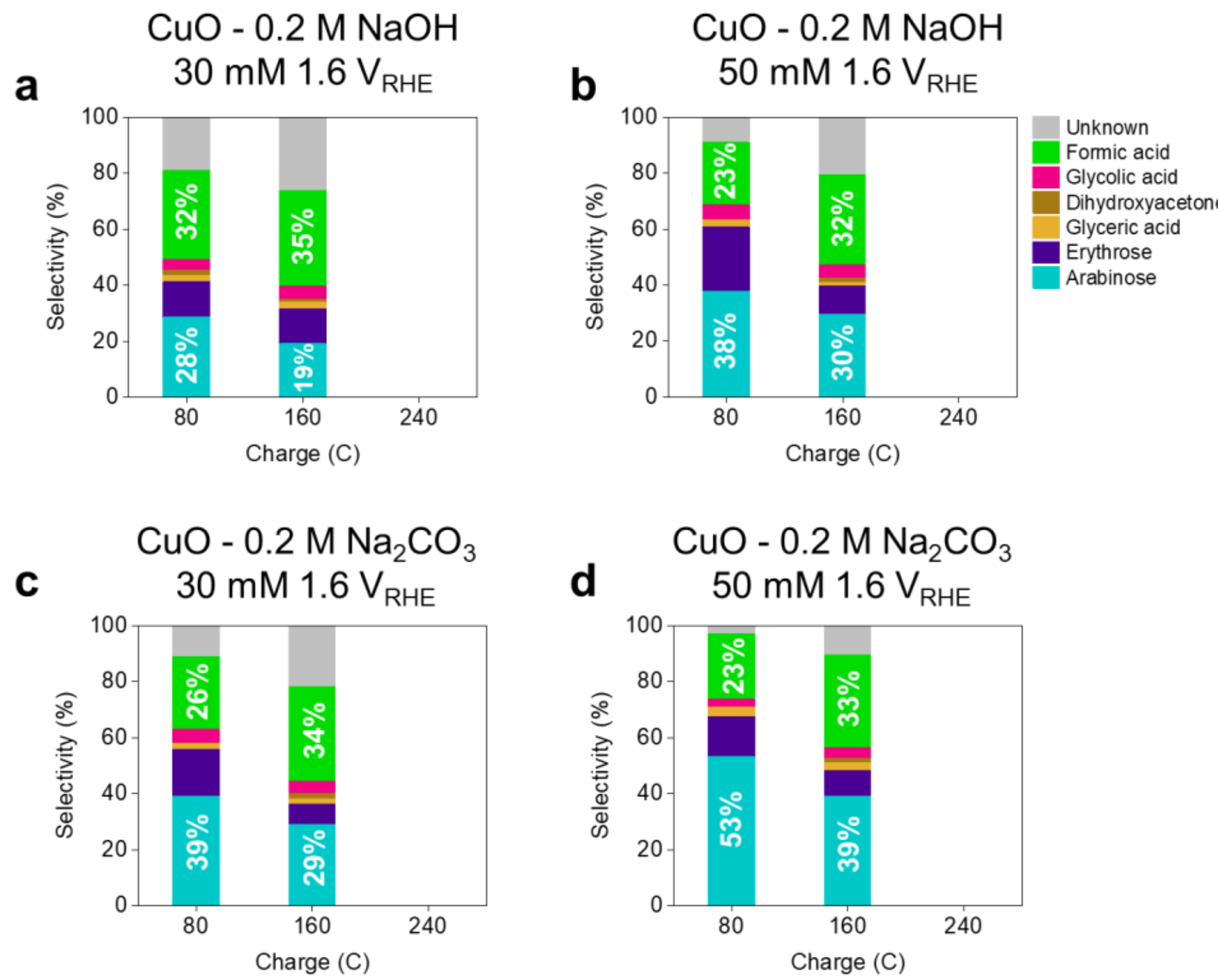

Figure S13. Product selectivity of GLUOR on CuO at higher GLU concentrations. Electrolysis was conducted at 1.6 V_{RHE} with (a) 30 mM and (b) 50 mM GLU in 0.2 M NaOH, and (c) 30 mM and (d) 50 mM GLU in 0.2 M Na_2CO_3. Electrolysis could not be continued to 240 C because the current decreased as the pH dropped during electrolysis (0.2 M NaOH: pH 13.2 to 6.4-7.7 and 0.2 M Na_2CO_3: pH 12 to 8.5).

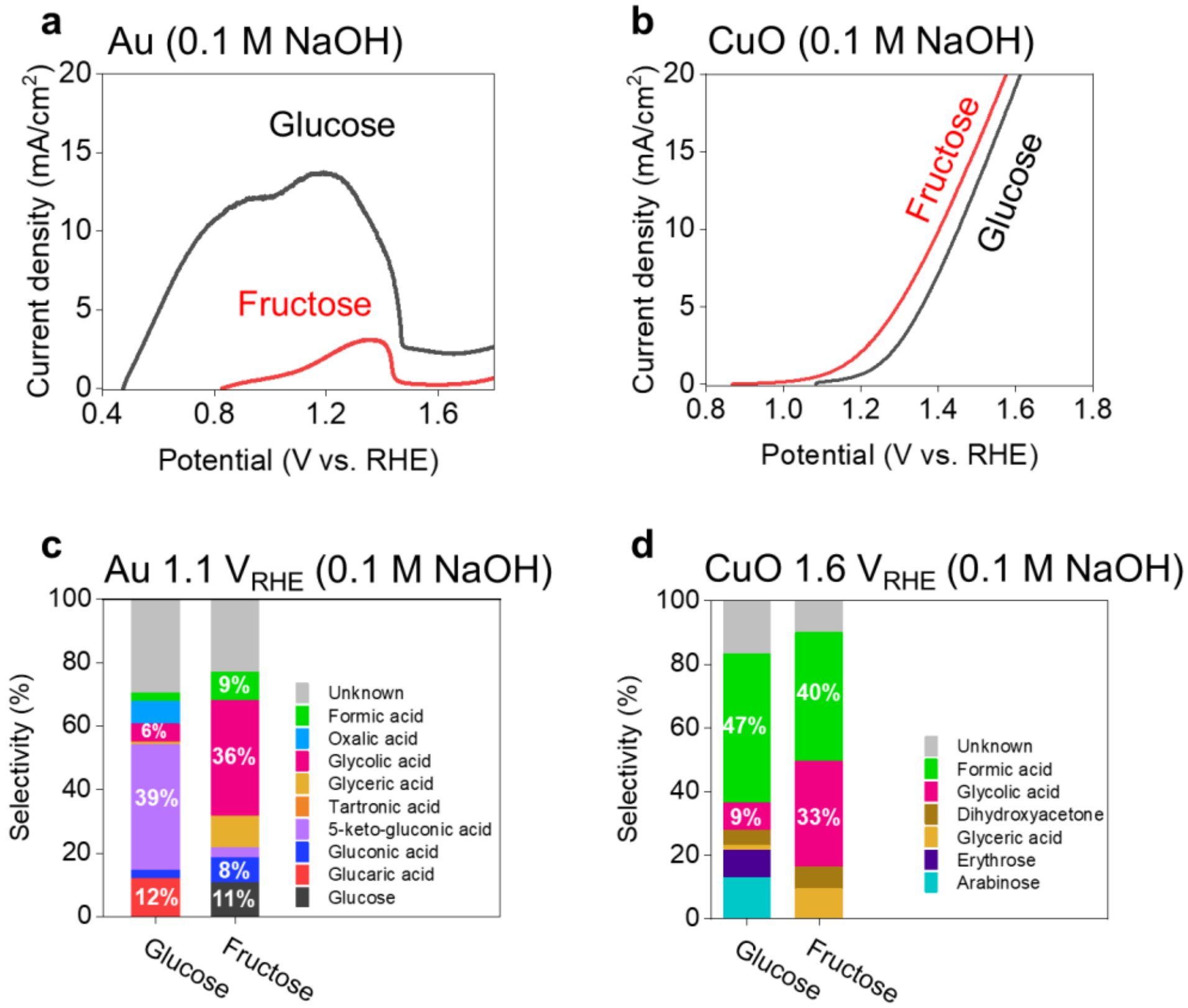

Figure S14. Electrochemical fructose oxidation on Au/C-foam and CuO. LSV curves recorded at 10 mV s^{-1} on (a) Au/C-foam and (b) CuO with 10 mM GLU and fructose in 0.1 M NaOH solution. Product distributions at selected potentials after 80 C passed on (c) Au/C-foam and (d) CuO.

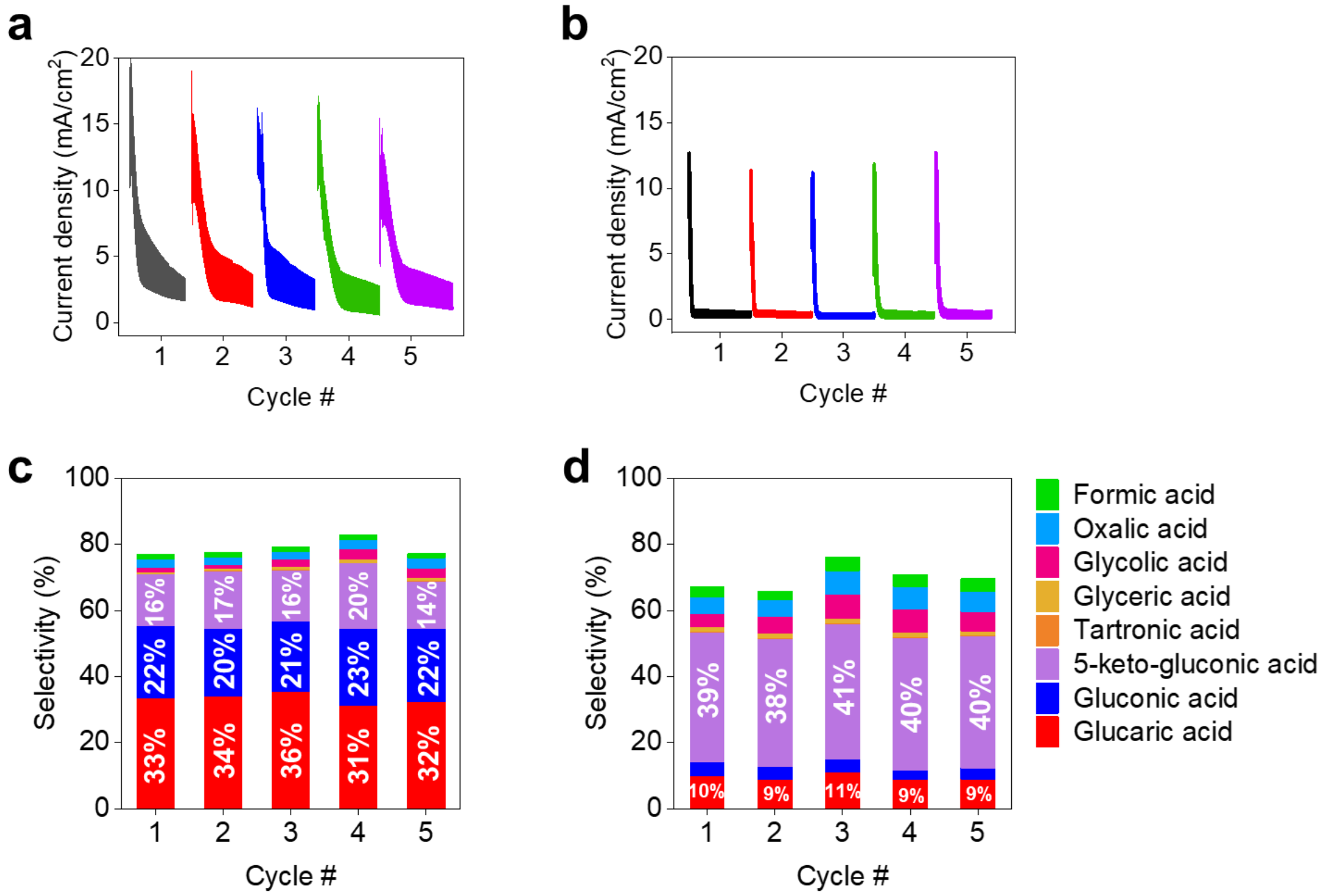

Figure S15. Stability of Au/C-foam during repeated electrolysis. Electrolysis was repeated for five cycles at 1.1 V_{RHE}, replacing the electrolyte with a fresh 10 mM GLU solution after each cycle. (a,b) *j-t* curves and (c,d) product selectivity of each cycle in (a,c) 0.1 M Na_2CO_3 and (b,d) 0.1 M NaOH.

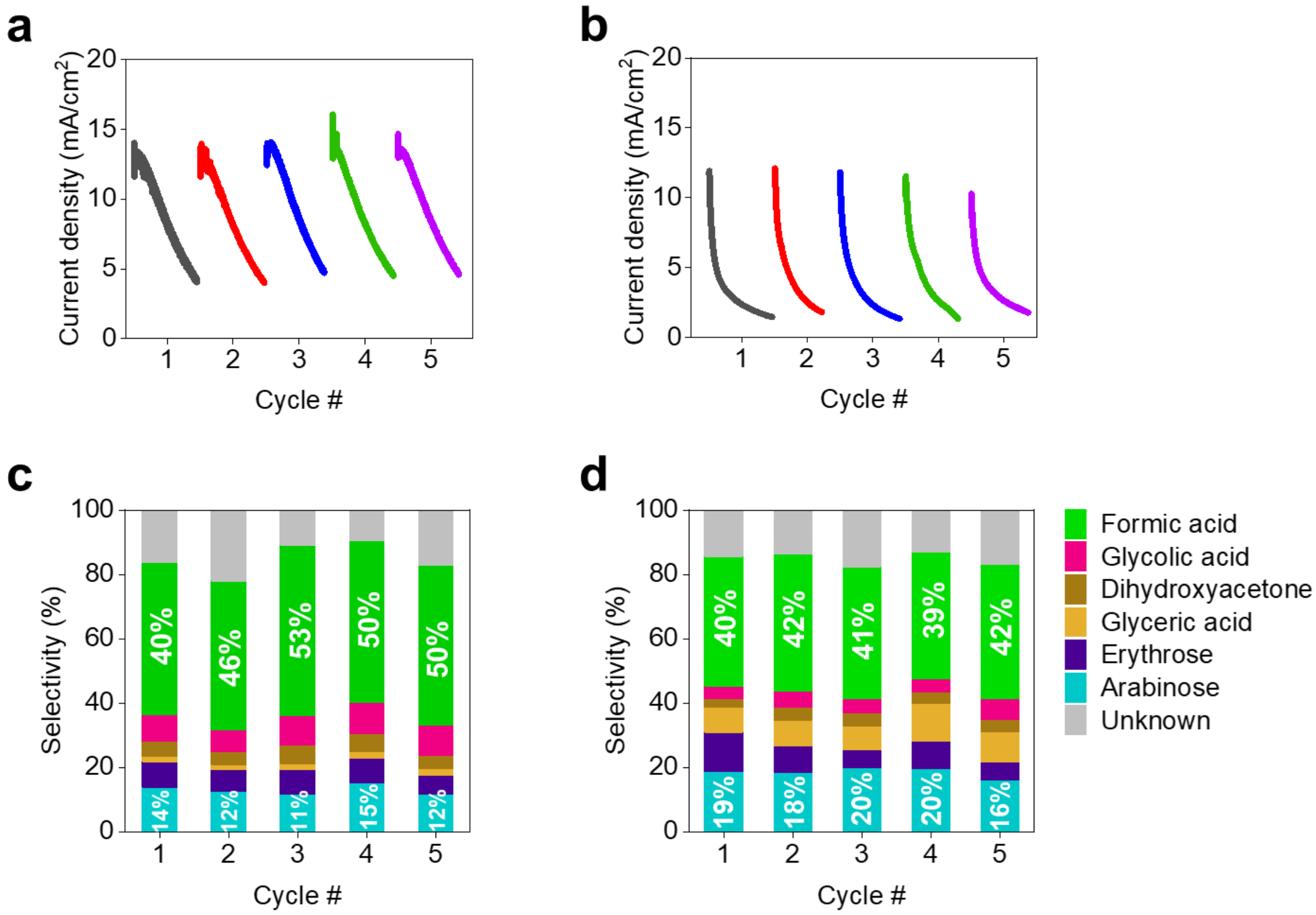

Figure S16. Stability of CuO during repeated electrolysis. Electrolysis was repeated for five cycles at 1.6 V_{RHE}, replacing the electrolyte with a fresh 10 mM GLU solution after each cycle. (a,b) *j-t* curves and (c,d) product selectivity of each cycle in (a,c) 0.1 M Na_2CO_3 and (b,d) 0.1 M NaOH.

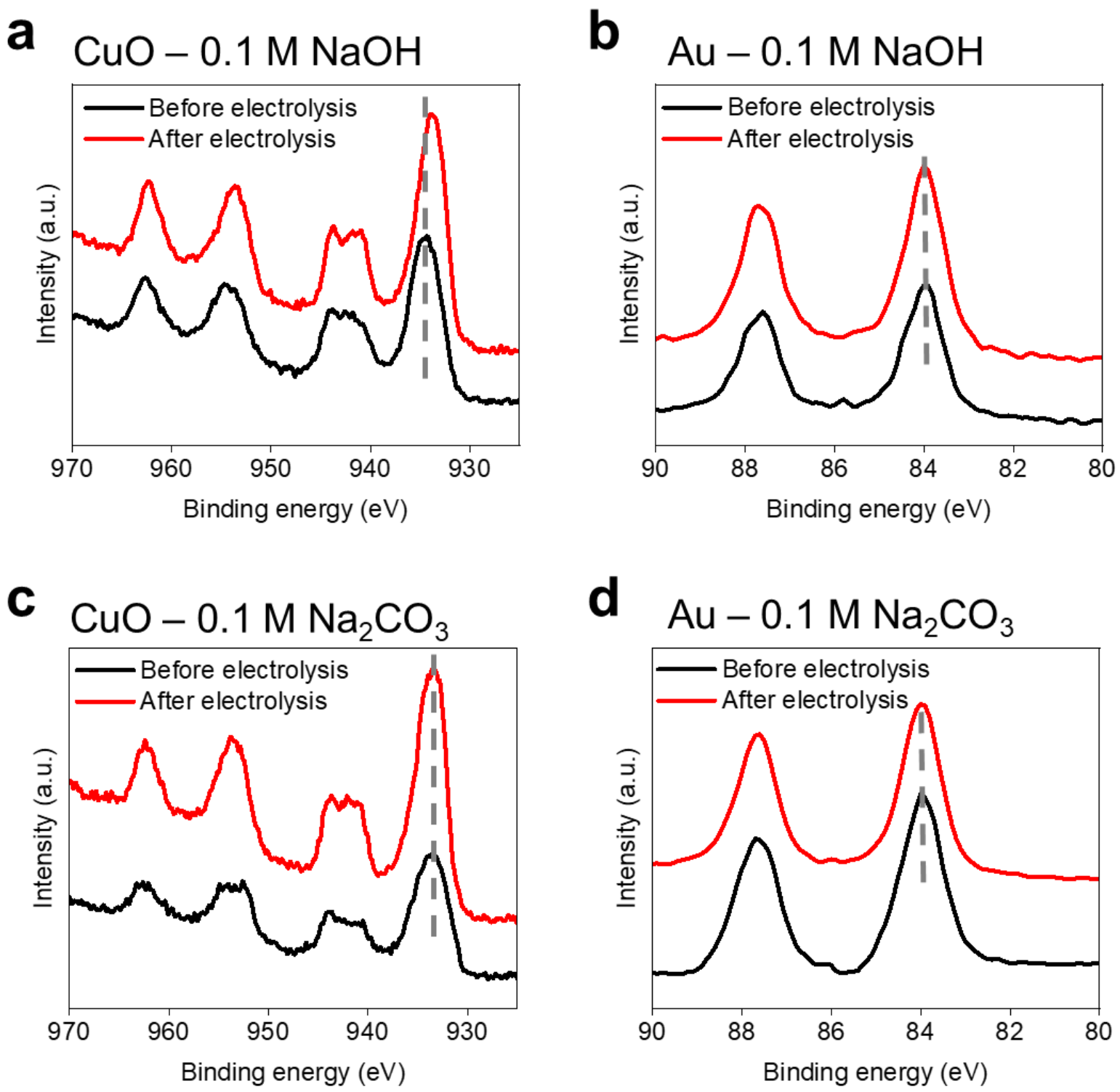

e

Catalyst	Electrolyte	Before reaction (eV)	After reaction (eV)
Au ($4f_{7/2}$)	0.1 M Na_2CO_3	84.0	84.0
	0.1 M NaOH	84.0	84.0
CuO ($2p_{3/2}$)	0.1 M Na_2CO_3	934.3	934.0
	0.1 M NaOH	933.5	933.5

Figure S17. XPS spectra of the catalysts before and after five-cycle electrolysis. Cu 2p spectra of CuO in (a) 0.1 M NaOH and (c) 0.1 M Na_2CO_3, and Au 4f spectra of Au/C-foam in (b) 0.1 M NaOH and (d) 0.1 M Na_2CO_3. (e) Binding energies of Au $4f_{7/2}$ and Cu $2p_{3/2}$ before and after the reaction. Metallic Au and Cu^{2+} are retained after electrolysis.

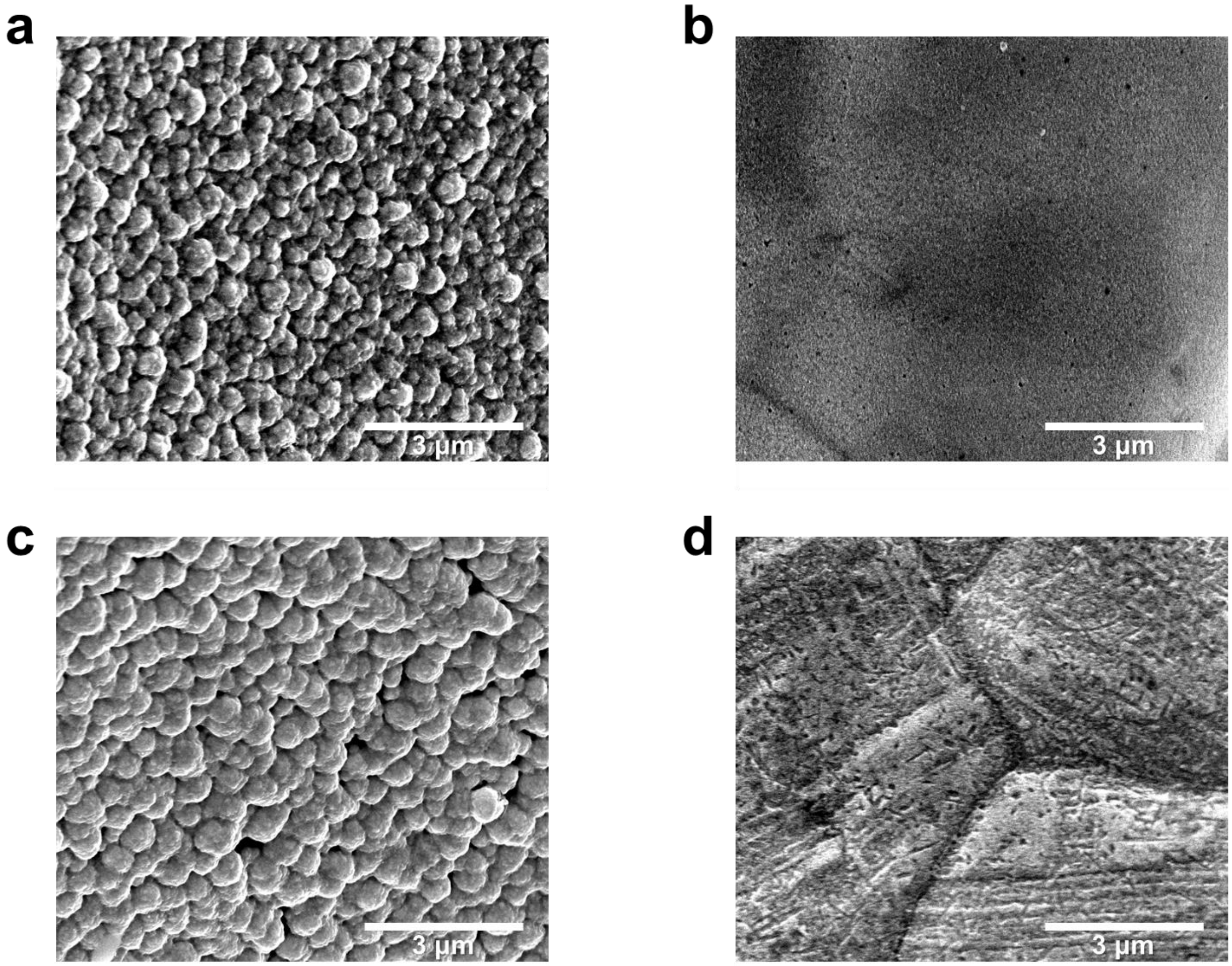

Figure S18. SEM images of the catalysts after five-cycle electrolysis. SEM images of (a,c) Au/C-foam and (b,d) CuO after electrolysis in (a,b) 0.1 M Na_2CO_3 and (c,d) 0.1 M NaOH.

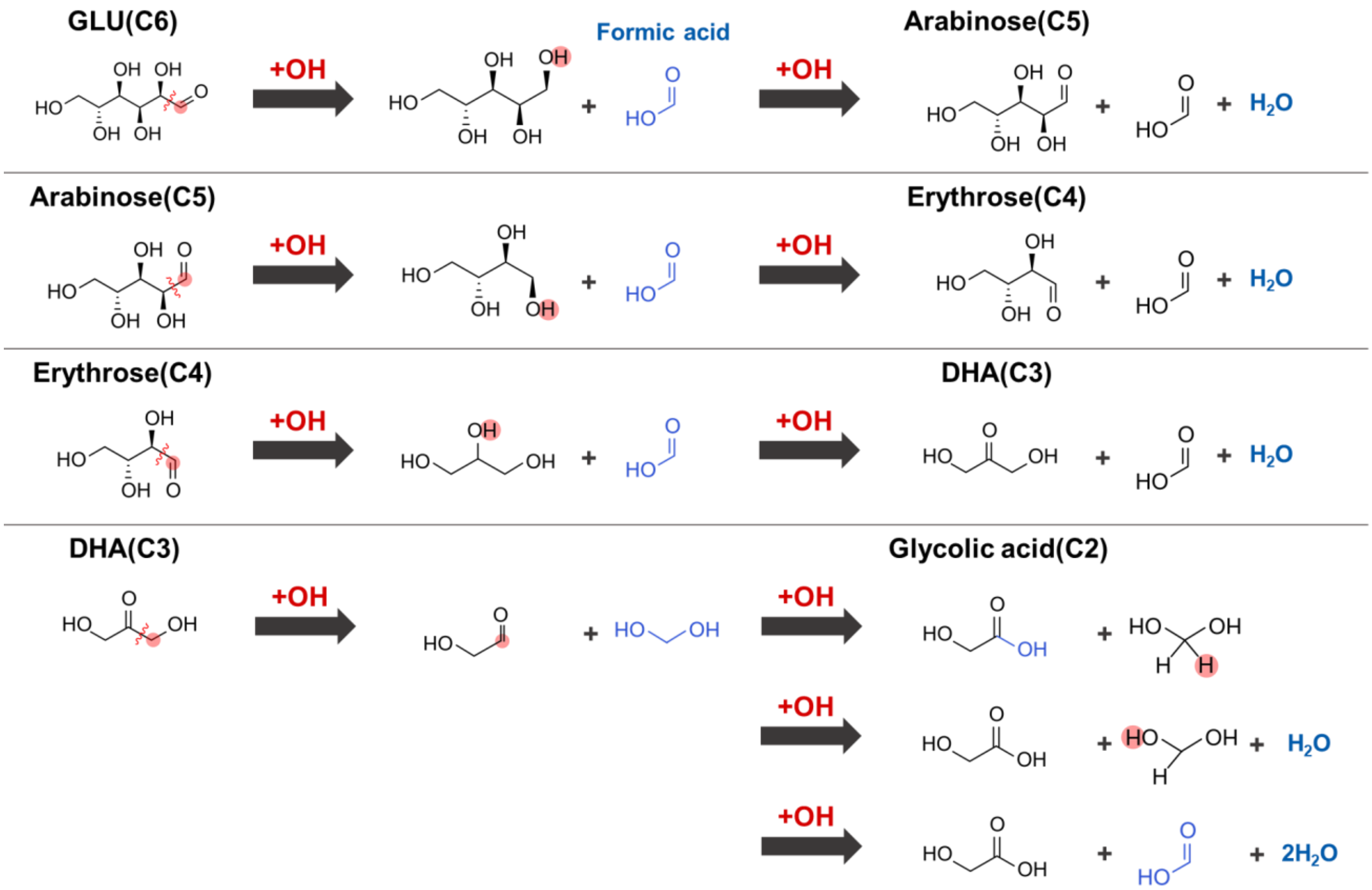

Figure S19. Reaction mechanism for C-C bond cleavage. DFT energy calculations for C-C bond cleavage were performed based on this mechanism.

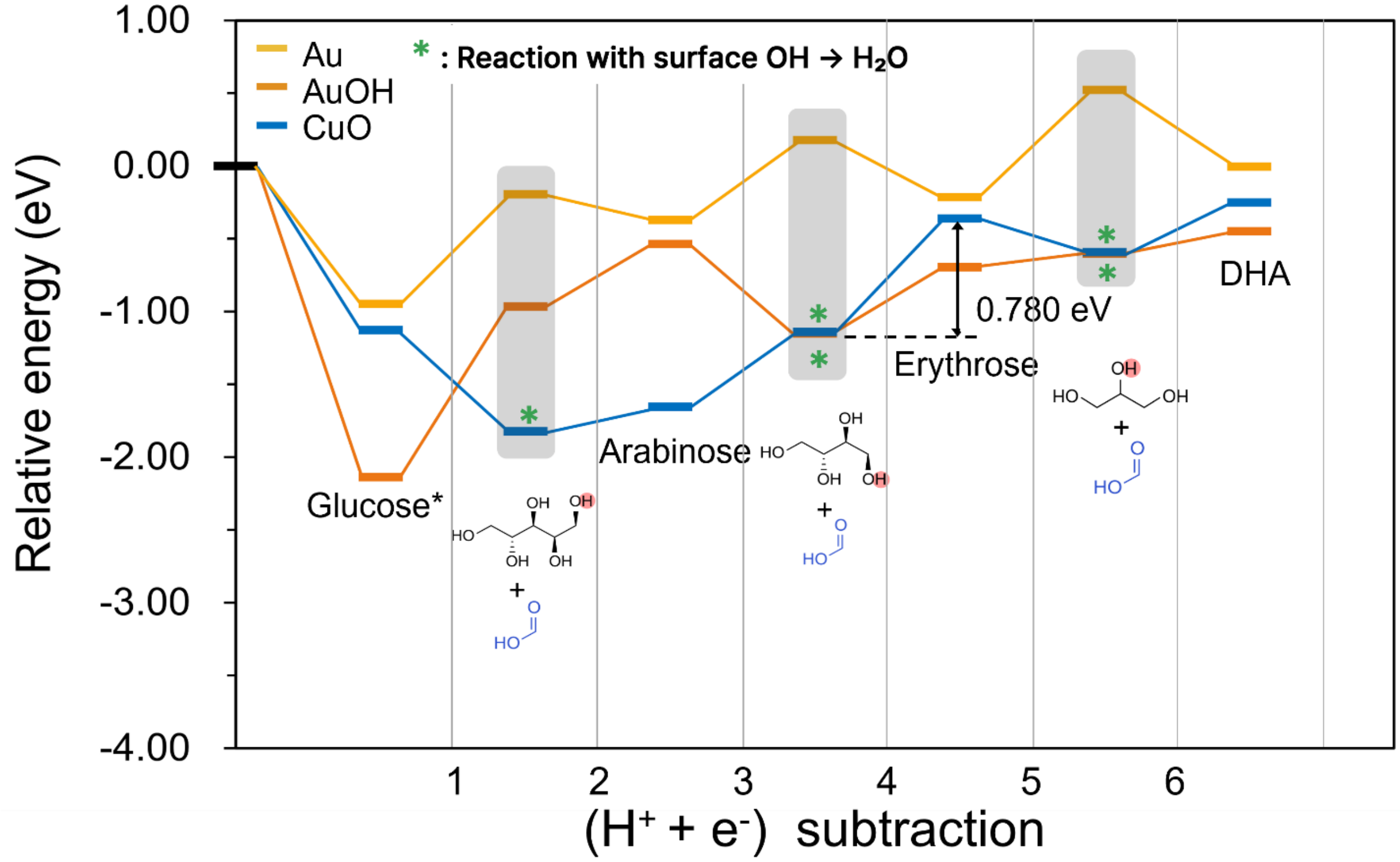

Figure S20. Overall energy profile for C-C bond cleavage.

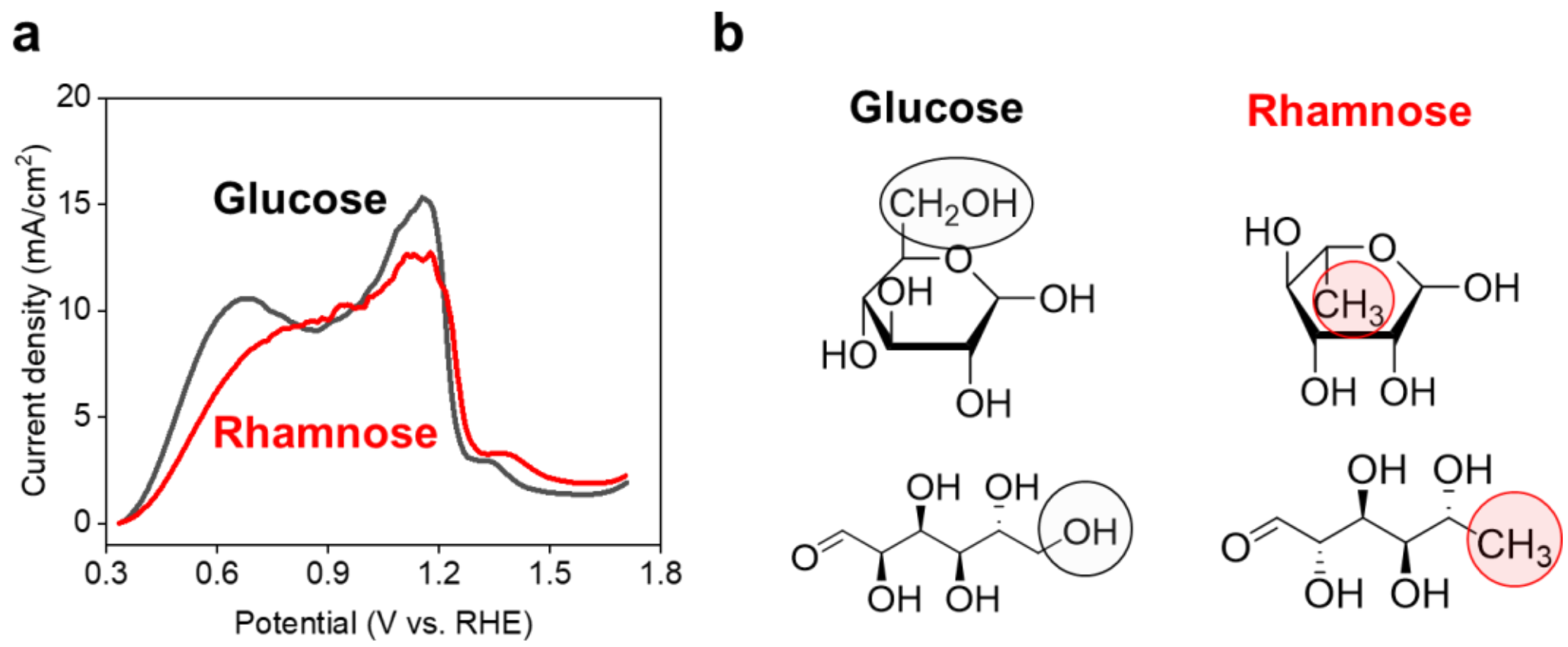

Figure S21. Effect of the C6 terminal group on GLUOR on Au. (a) LSV curves of 10 mM GLU and rhamnose recorded on Au in 0.1 M NaOH. (b) Structures of GLU and rhamnose in the pyranose and open-chain forms.

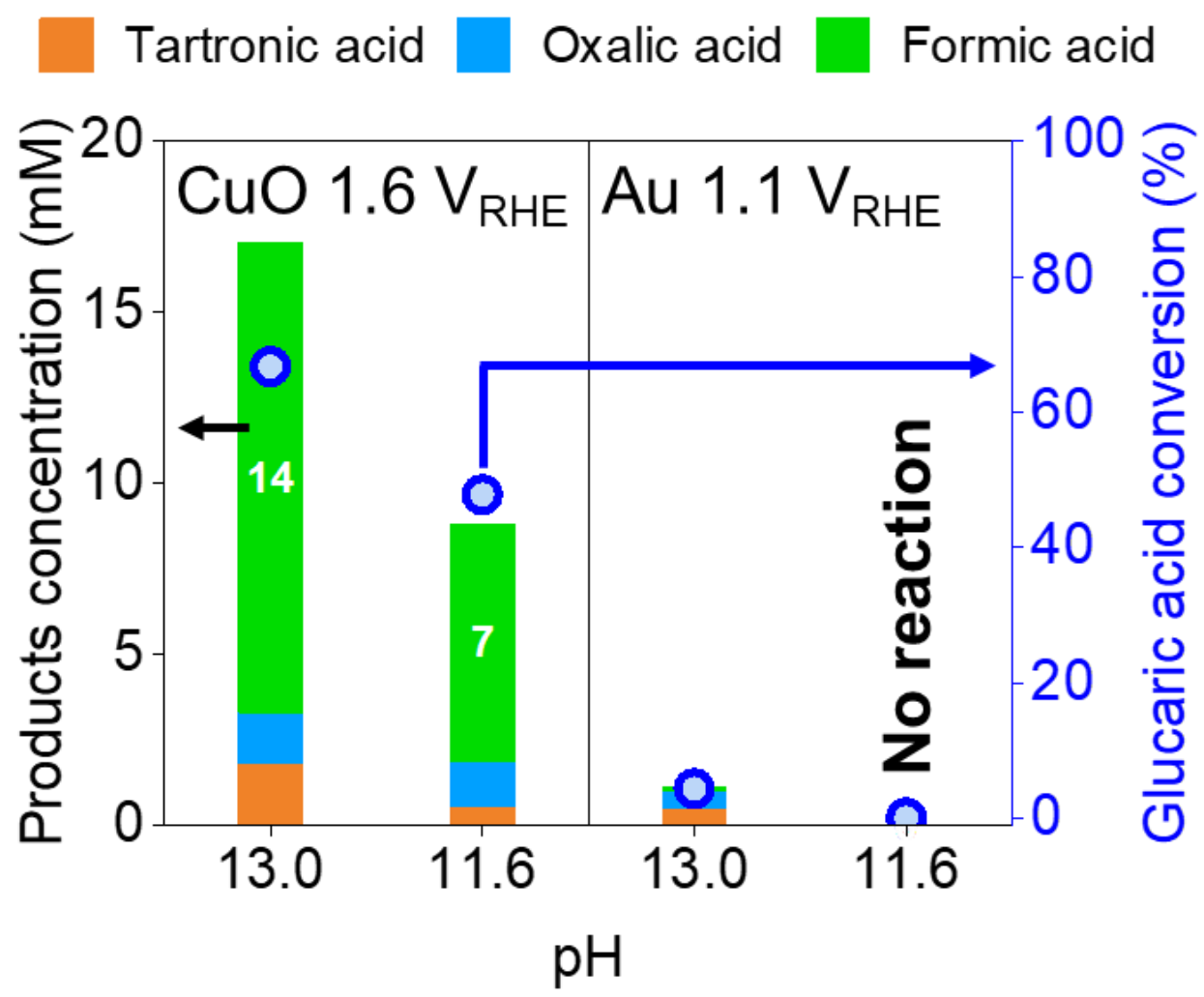

Figure S22. Product distribution after electrochemical glucaric acid (GRA) oxidation on CuO and Au. Reaction time was ~1 h for each pH and potential.

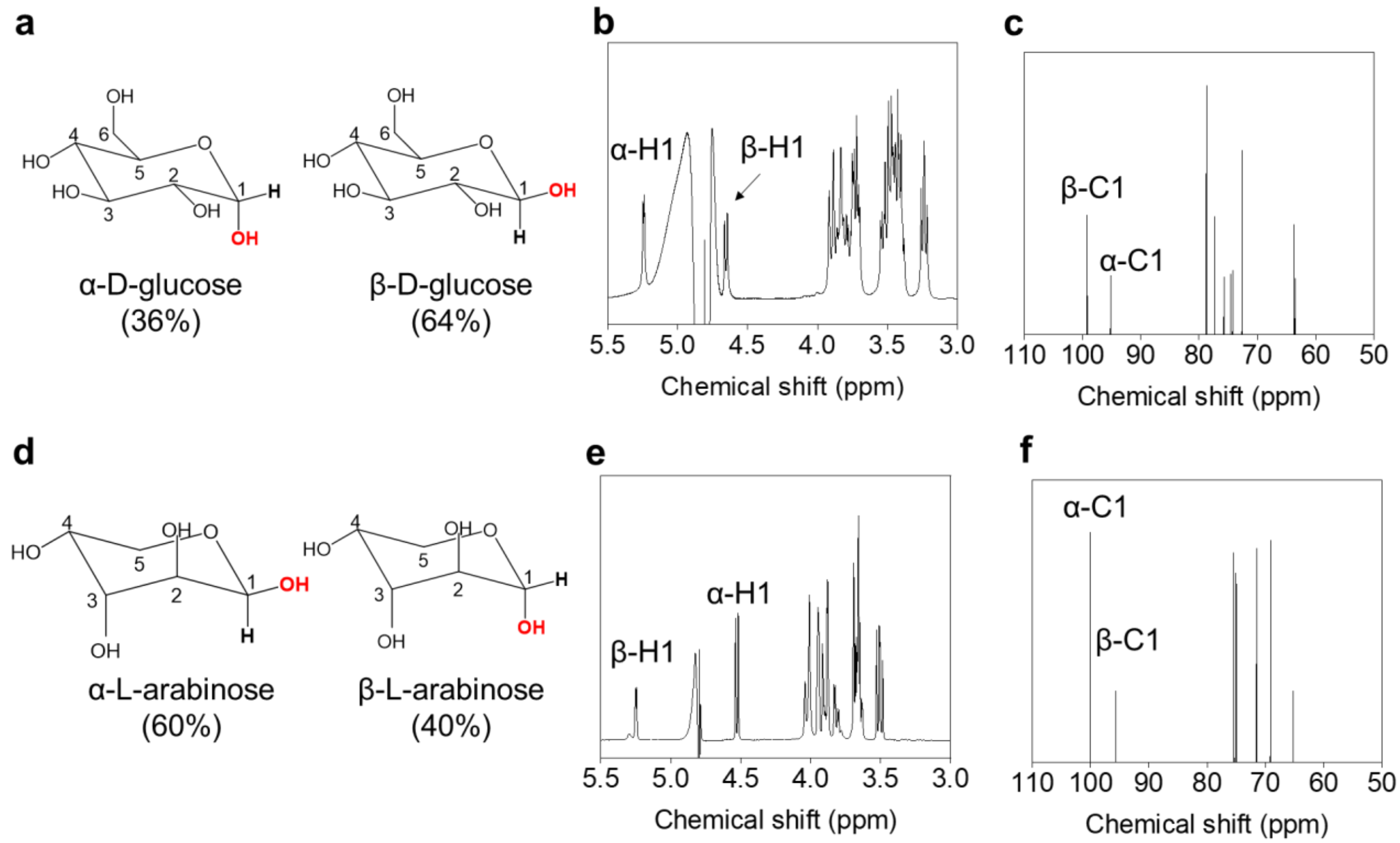

Figure S23. Major structure of GLU and arabinose in 0.1 M Na_2CO_3 and their NMR spectra. (a,d) Major structures of GLU and arabinose in solution. (b,e) ^{1}H NMR spectra of GLU and arabinose. (c,f) ^{13}C NMR spectra of GLU and arabinose. GLU and arabinose exist in the pyranose form in aqueous solution. The number in parentheses indicates the percentage of structures that exist in nature. The open-chain forms of GLU and arabinose account for less than 1%. Alpha and beta denote the anomers that differ in the configuration of the anomeric C1-OH group [1, 2].

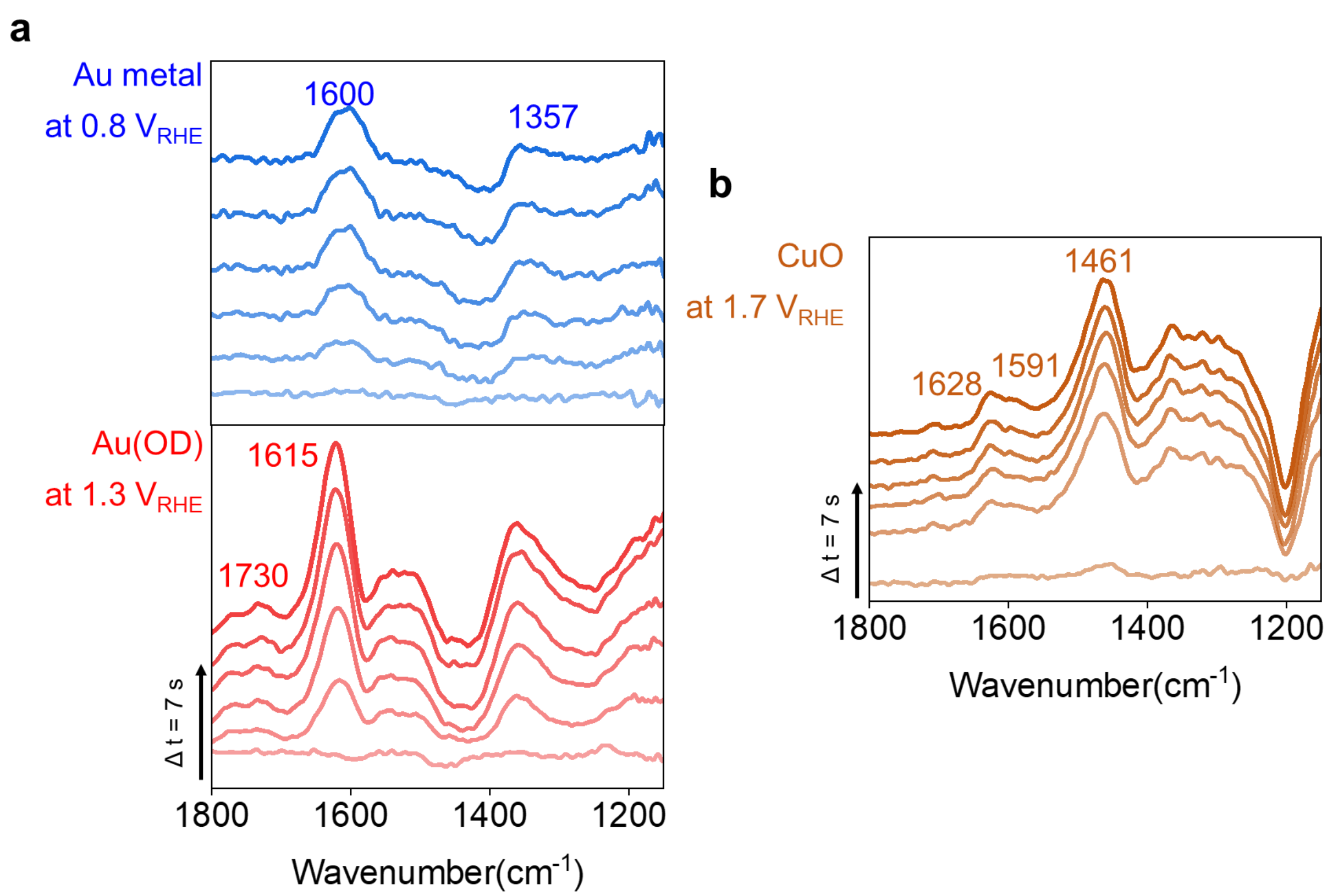

Figure S24. ATR-FTIR spectra of adsorbed species during GLUOR in 0.1 M Na_2CO_3 solution. (a) Au and (b) CuO. Bicarbonate (1628 cm^{-1}) is formed during the reaction.

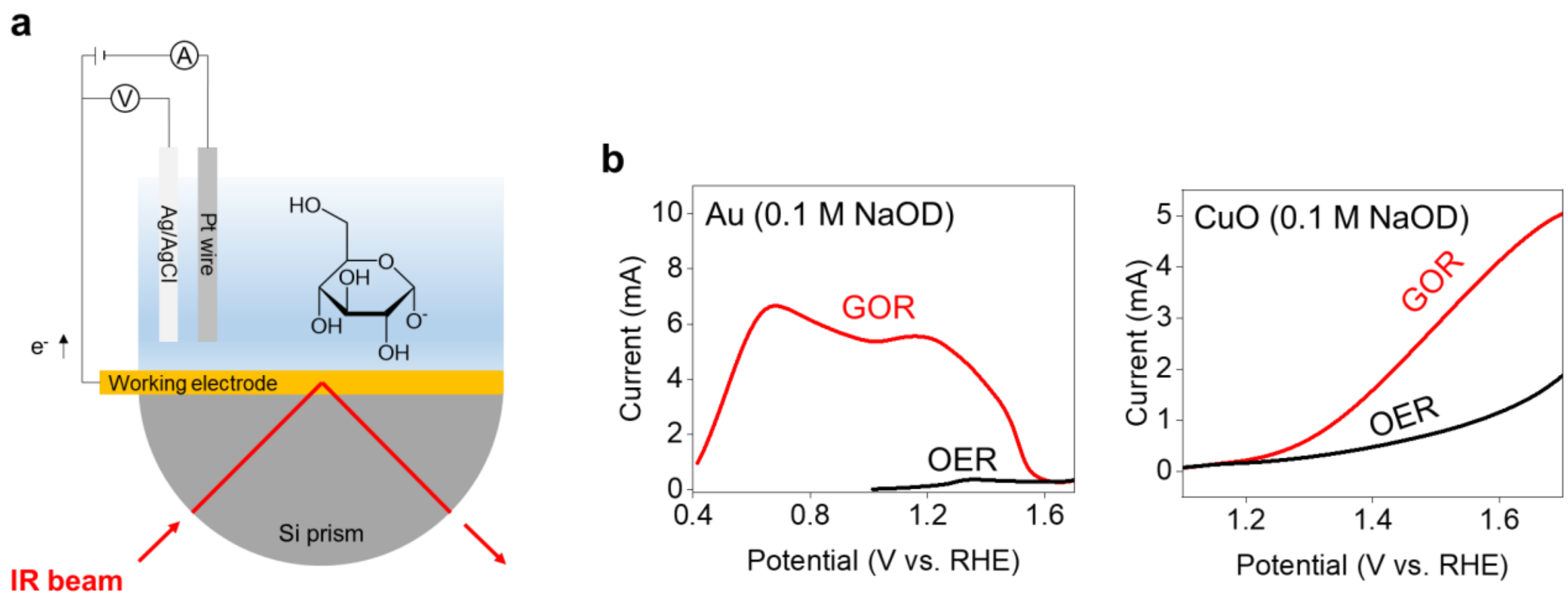

Figure S25. Schematic illustration of in situ ATR-FTIR experiments and LSV curves. (a) Experimental setup for in situ ATR-FTIR measurements. (b) LSV curves of Au and CuO electrodes recorded at 10 mV s^{-1} in 0.1 M NaOD with 100 mM GLU. The curves in the absence of GLU are shown as black lines.

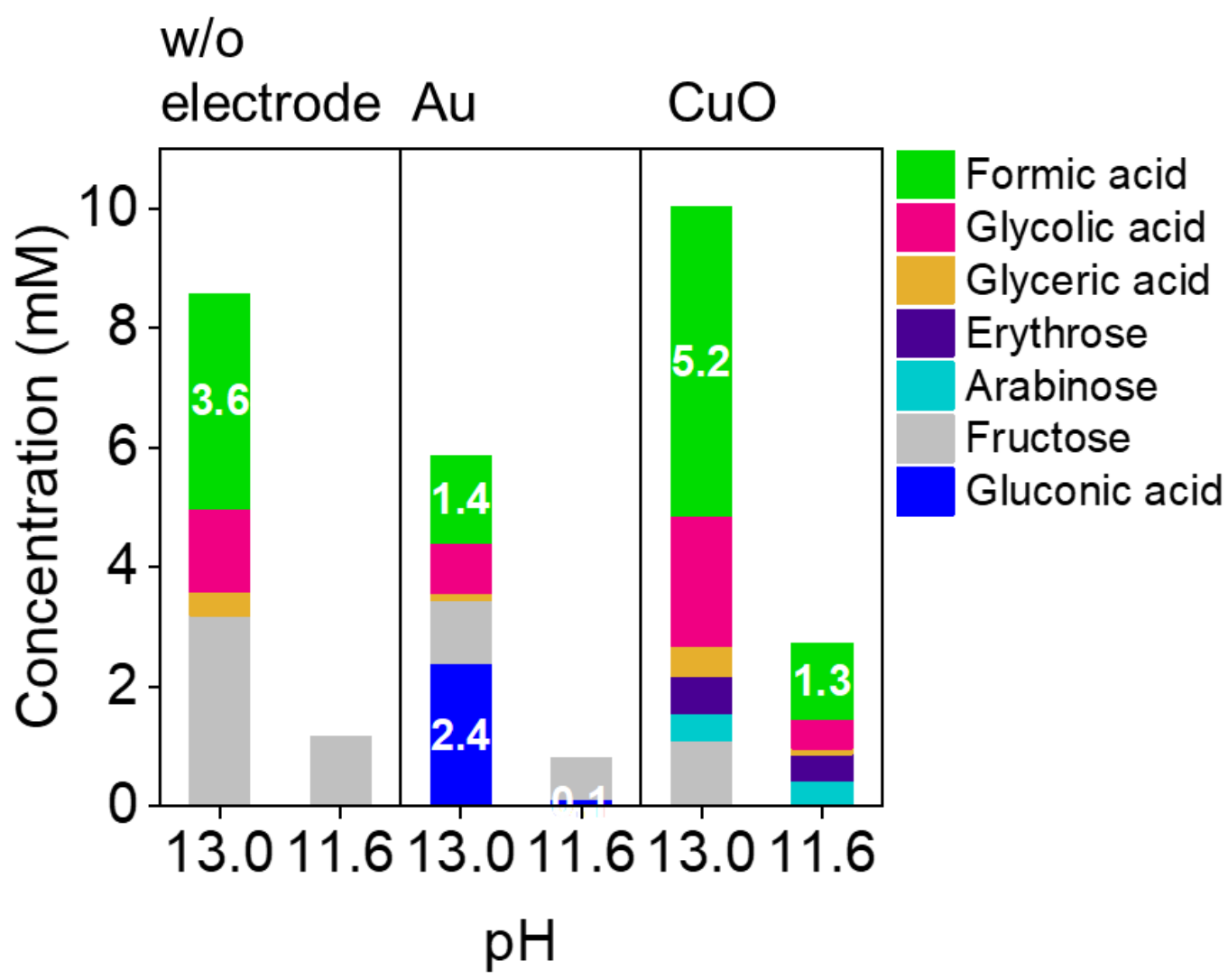

Figure S26. Products under open-circuit conditions. GLU was converted to small molecules and C6 products in alkaline solutions. All reactions were conducted for 24 h. GLU can be spontaneously oxidized to formic acid on Cu surfaces. If the ATR-FTIR peak at 1583 cm^{-1} observed on Au surfaces is associated with the gluconate species, Cu also exhibits an absorption band in the range of 1580-1590 cm^{-1}.

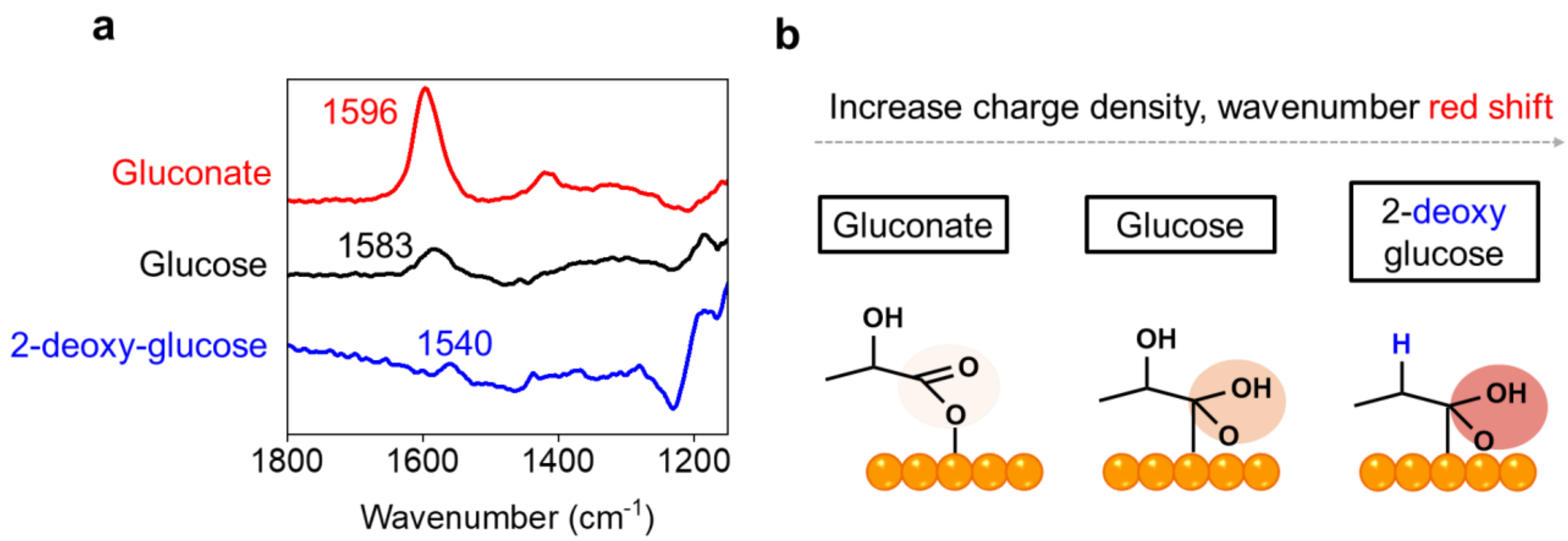

Figure S27. ATR-FTIR spectra of GLU derivatives. (a) ATR-FTIR spectra of GNA, GLU, and 2-deoxy-glucose. (b) Schematic illustration of possible adsorbates on metallic Au surfaces. For clarity, only the C1 and C2 positions are shown.

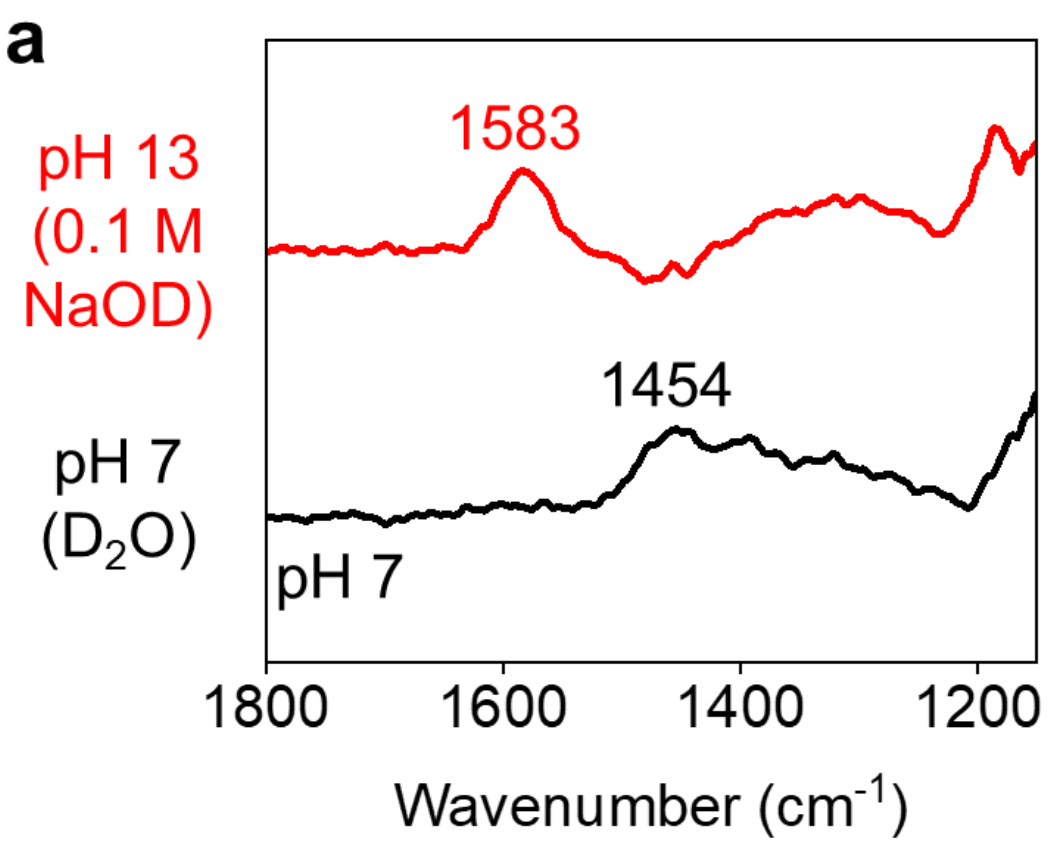

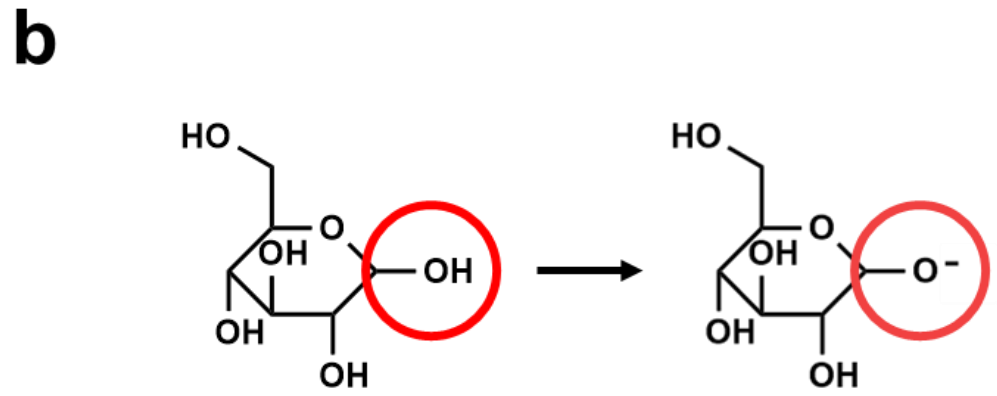

	OH	O^-
pH 13 (0.1 M NaOD)	11.2 %	88.8 %
pH 7(D_2O)	99.9 %	0.0008 %

Figure S28. pH-dependent ATR-FTIR spectra and deprotonation state of GLU. (a) ATR-FTIR spectra of GLU in 0.1 M NaOD and in D_2O. (b) Possible structure of GLU at pH 13 and pH 7. The fraction of protonated and deprotonated species was estimated using the Henderson-Hasselbalch equation. The anomeric OH group can be deprotonated under strongly alkaline conditions.

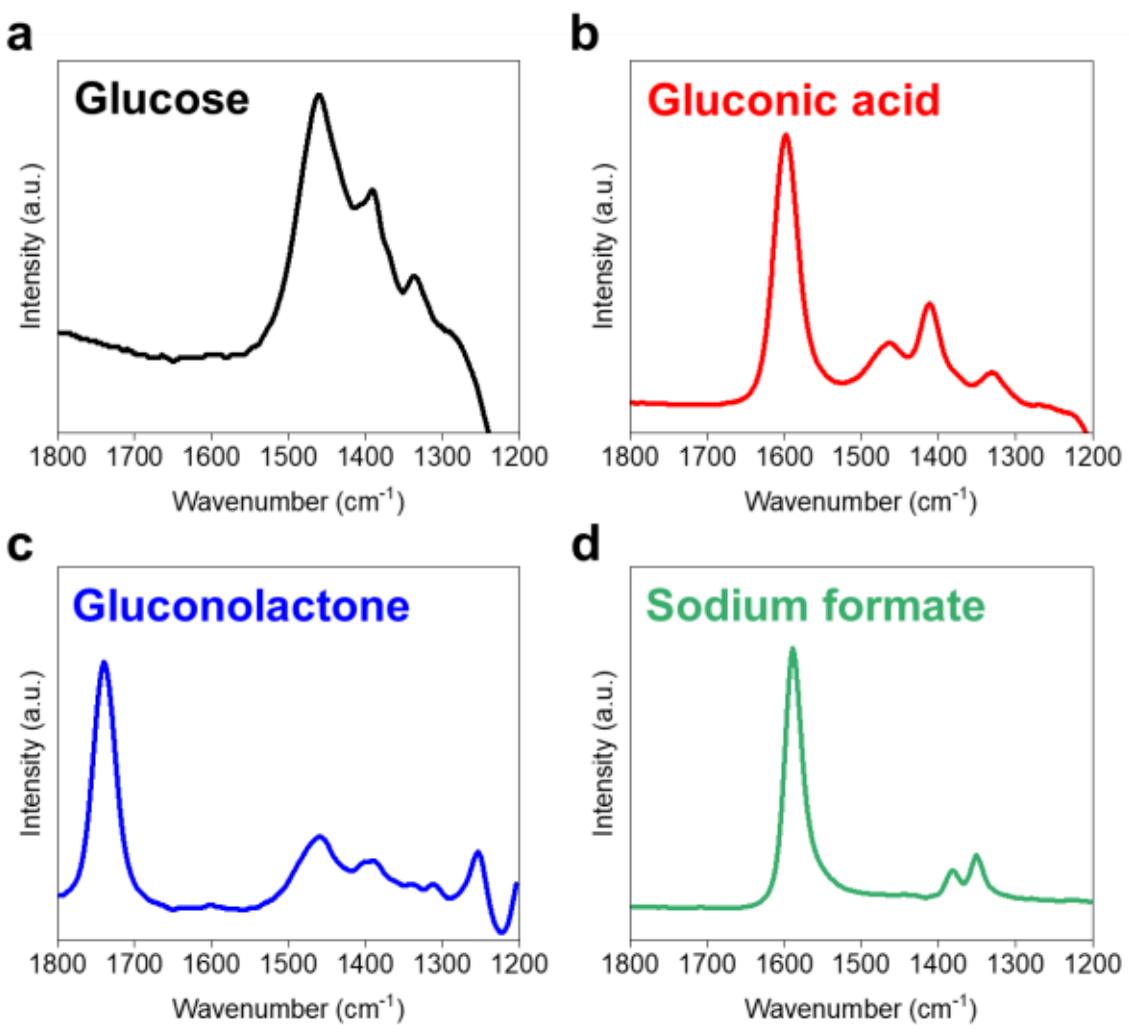

e

Wavenumber (cm^{-1})	Species	Assignments
1597	Gluconate	Asymmetric COO^- stretching of carboxylate group
1589	Formate	
1740	Glucono-lactone	Vibration of carbonyl group
1466 and 1390	Glucose	OCH, CH_2 bending

Figure S29. Reference ATR-FTIR spectra. ATR-FTIR spectra of (a) glucose, (b) gluconic acid, (c) gluconolactone and (d) sodium formate measured on the SiO_2 prism in D_2O. (e) Characteristic IR bands and their assignments.

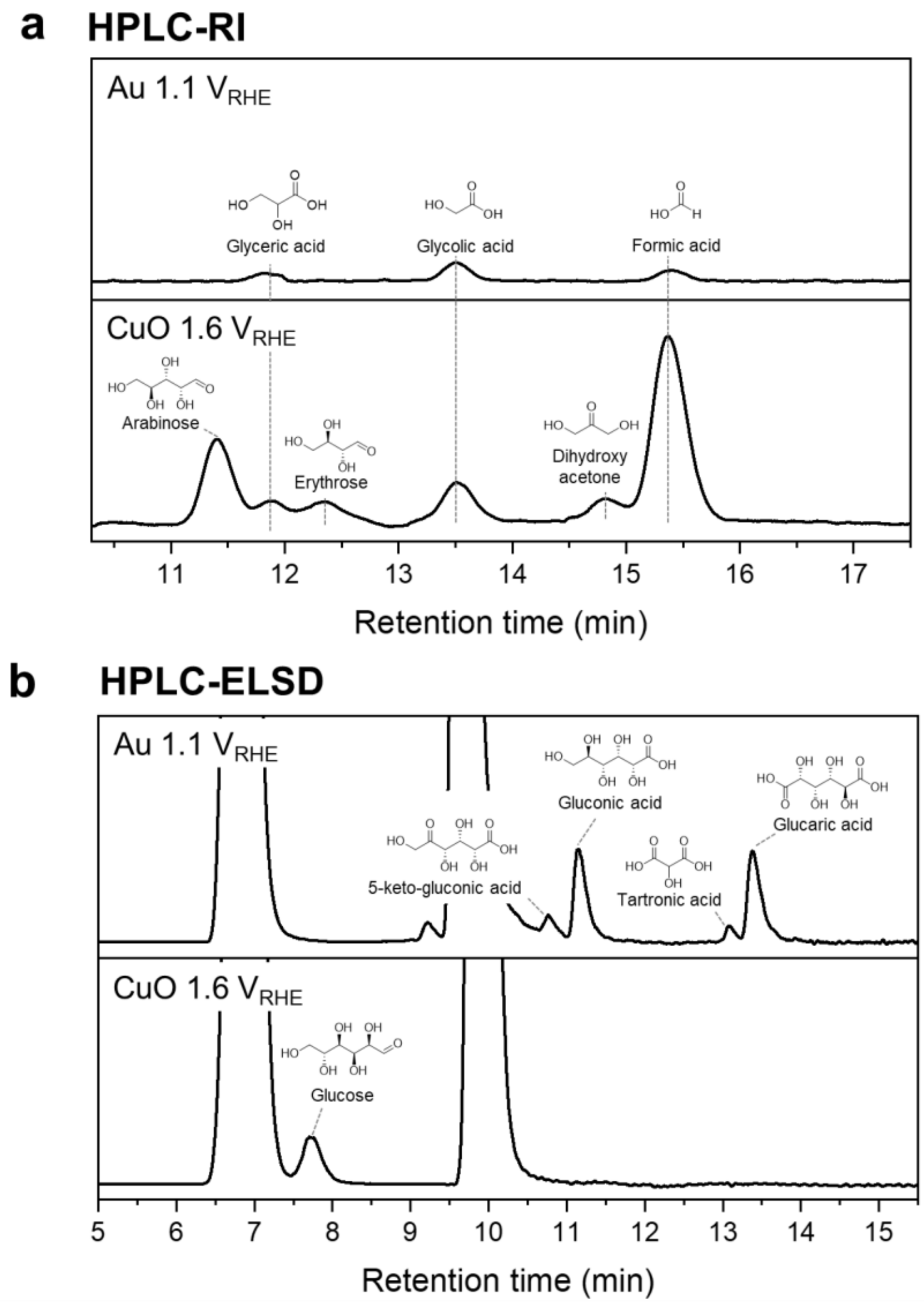

Figure S30. HPLC chromatograms after GLUOR in 0.1 M Na_2CO_3 solution. (a) The ≤C5 products were analyzed with a refractive index detector using a Bio-Rad Aminex HPX-87H column. Oxalic acid was omitted from the figure to improve clarity. Its retention time is 7.5 min. (b) GLU, C6 products, and tartronic acid were analyzed with an evaporative light scattering detector using a Poroshell 120 HILIC-Z column.

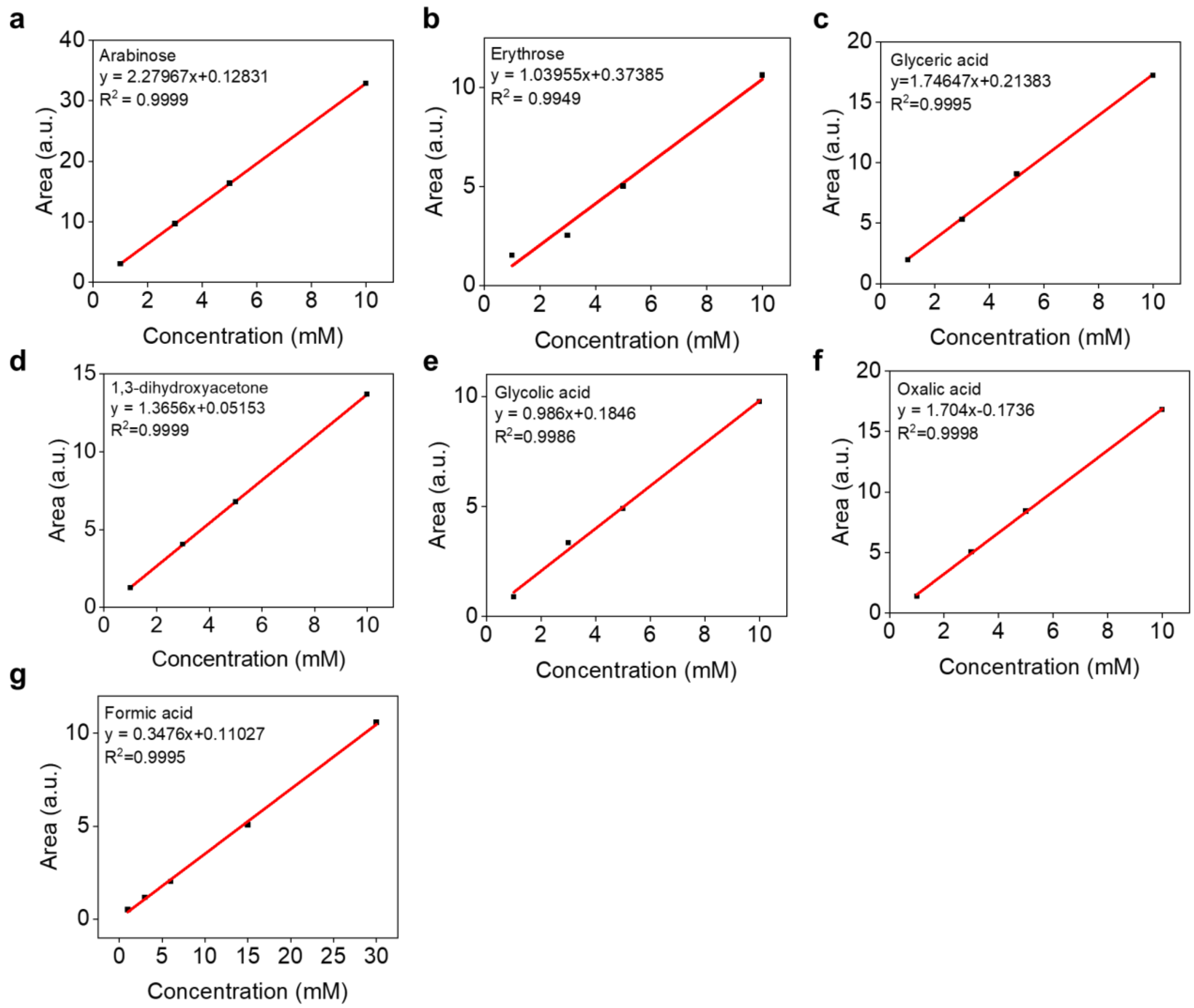

Figure S31. HPLC-RI calibration. HPLC calibration curves using a refractive index detector for arabinose (a), erythrose (b), glyceric acid (c), 1,3-dihydroxyacetone (d), glycolic acid (e), oxalic acid (f), and formic acid (g).

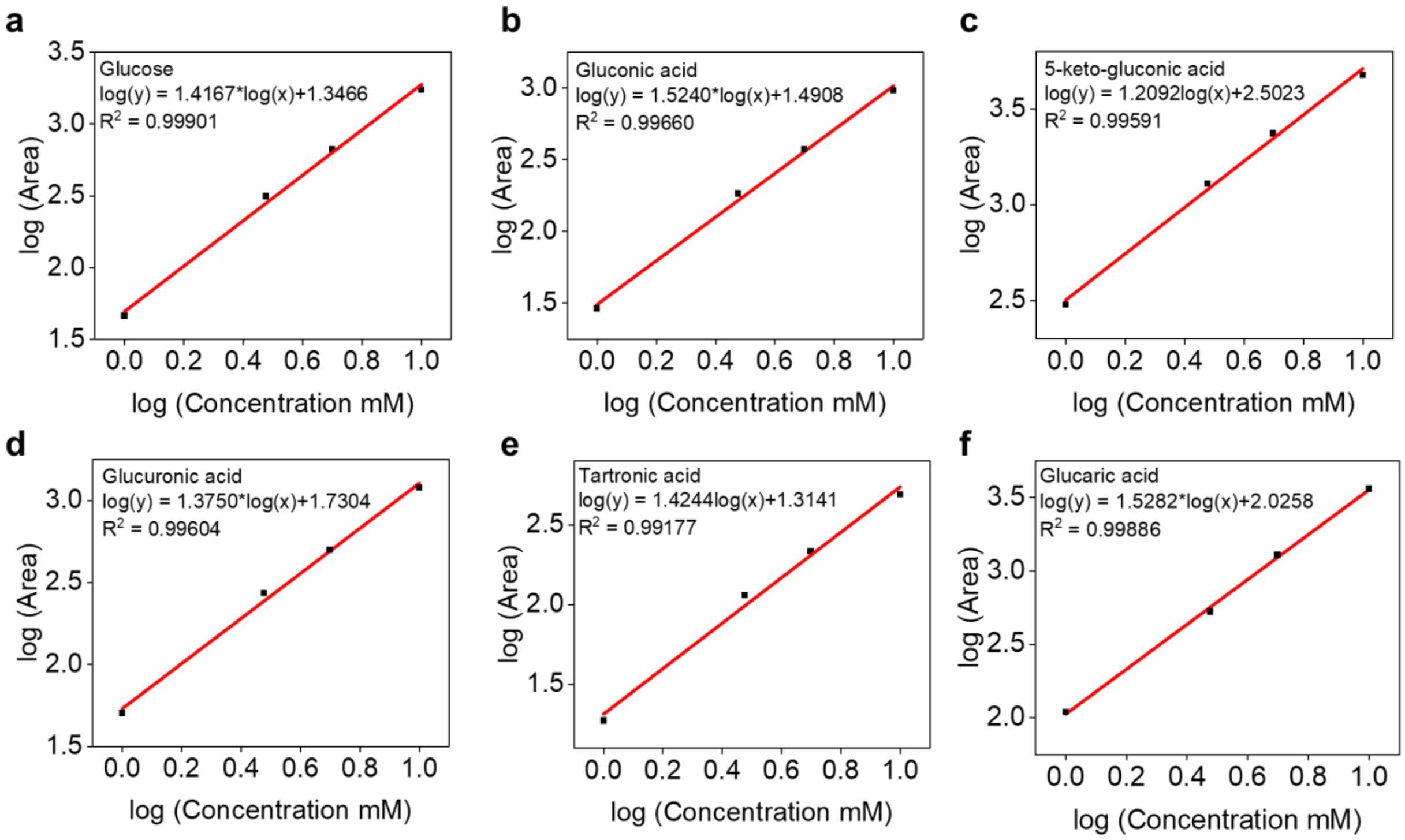

Figure S32. HPLC-ELSD calibration. HPLC calibration curves using an evaporative light scattering detector (ELSD) for glucose (GLU) (a), gluconic acid (GNA) (b), 5-keto-gluconic acid (c), glucuronic acid (d), tartronic acid (e), and glucaric acid (f).

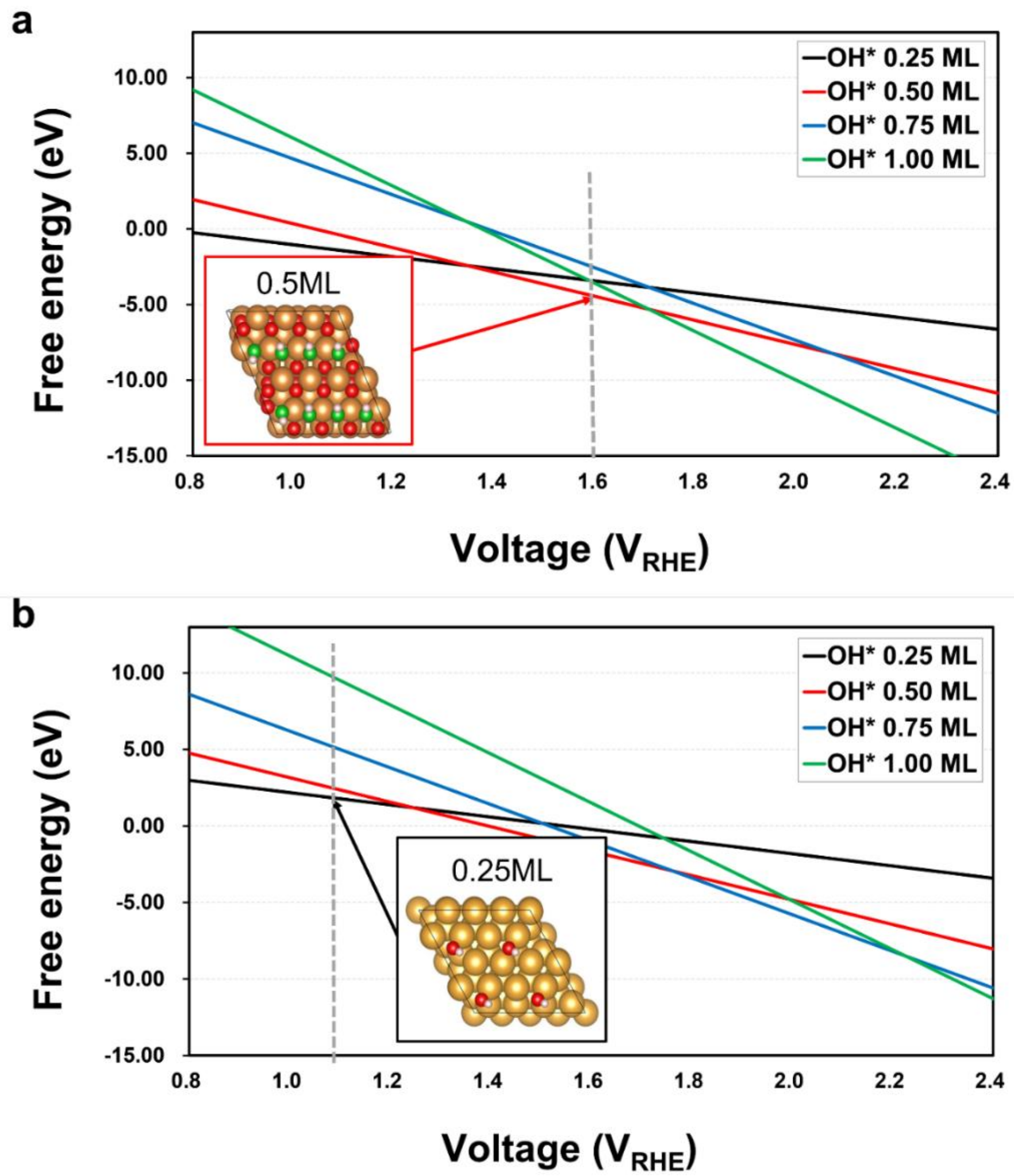

Figure S33. Surface Pourbaix diagrams for each surface. (a) Free energies of different OH-group surface coverages as a function of potential on CuO(111). (b) Free energies of different OH-group surface coverages as a function of potential on Au(111). Under the experimental conditions (CuO = 1.6 V_{RHE}, Au = 1.1 V_{RHE}), 0.5 monolayer (ML) on CuO and 0.25 ML on Au are thermodynamically stable.

Table S1. Comparison of reported electrochemical glucose oxidation.

Metal	No.	Catalyst	Electrolyte	Potential	Glucaric acid	Formic acid	Reference
Ni	1	$NiFeO_x$	1 M KOH	1.3 V_{RHE}	Yield 83%	-	Nat. Commun., 2020, 11, 265.
	2	Ni foam	1 M KOH	1.3 V_{RHE}	Yield 17.2%	-	
	3	NiFe LDH	1 M KOH	1.45 V_{RHE}	Yield 72.9%	-	ACS Appl. Mater. Interfaces, 2024, 16, 20, 26107-26120
	4	NiO+TEMPO	0.1 M K_2CO_3	1.44 V_{RHE}	Selectivity 83%	-	J. Energy Chem., 2023, 80, 58-67
	5	$Ni(OH)_2$-MoO_3	1 M KOH	1.5 V_{RHE}	-	Yield 90.5%	ChemSusChem, 2025, 18, e202500297
	6	NiSn/CNT	1 M KOH	1.45 V_{RHE}	-	FE 93%	ChemSusChem, 2025, 18, e202401256
	7	Ni foam	0.1 M KOH	1.6 V_{RHE}	-	Selectivity 182%	Electrochim. Acta, 2024, 487, 144159
	8	Ni aerogels	1 M KOH	1.6 V_{RHE}	-	Yield 83.5%	Chem. Sci., 2025, 16, 21094-21103
Cu	9	Cu	0.1 M NaOH	0.84 V_{RHE}	Yield 38.4%	Yield 13.9%	ChemElectroChem, 2020, 7, 86-95
	10	$Cu(OH)_2$	1 M KOH	0.92 V	FE 98.7% (gluconic acid)	-	Adv. Mater., 2021, 33, 2104791
	11	Cu	1 M NaOH	1.51 V_{RHE}	-	Yield 47.8%	RSC Adv., 2021, 11, 31208-31218
	12	CuO	1 M KOH	1.4 V_{RHE}	-	FE 95%	Sci. Bull., 2023, 68, 2982-2992
	13	Cu aerogel	1 M KOH	1.6 V_{RHE}	-	Yield 9.5%	Chem. Sci., 2025, 16, 21094-21103

Metal	No.	Catalyst	Electrolyte	Potential	Glucaric acid	Formic acid	Reference
Cu	14	CuO	0.1 M Na_2CO_3	1.6 V_{RHE}	-	Selectivity 49%	This work
Au	15	Au	0.1 M NaOH	1.34 V_{RHE}	Yield 13.5%	Yield 8.8%	ChemElectroChem, 2020, 7, 86-95
	16	Au	0.1 M KOH	1.28 V_{RHE}	Selectivity 83.4% (gluconic acid)	-	Electrochim. Acta, 2024, 487, 144159
	17	Au	0.1 M Na_2CO_3	0.6 → 1.1 V_{RHE}	Yield 87%	-	Electrochim. Acta, 2021, 374, 137852
	18	Au	0.1 M Na_2CO_3	1.1 V_{RHE}	Selectivity 34%	Selectivity 1.5%	This work

Table S2. Optimized structures for the step-by-step simulation of glucose C-C bond cleavage on Au(111), Au(OH)(111), and CuO(111) surfaces. The optimized geometries on (a) Au, (b) Au(OH), and (c) CuO surfaces are shown, including the intermediates formed at each step and their interactions with surface OH species.

a

@Au	Glucose(C6)	C6→C5	Arabinose(C5)	C5→C4
Structure	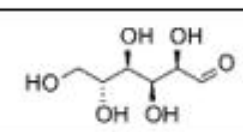	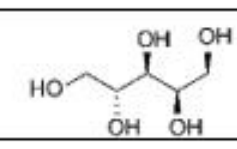	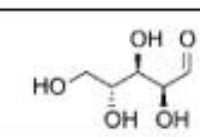	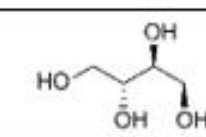
Adsorption model	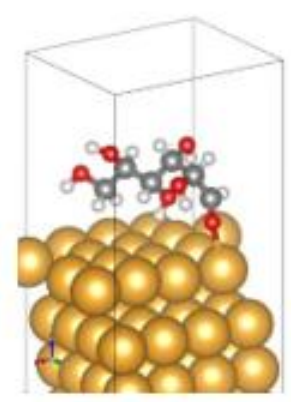	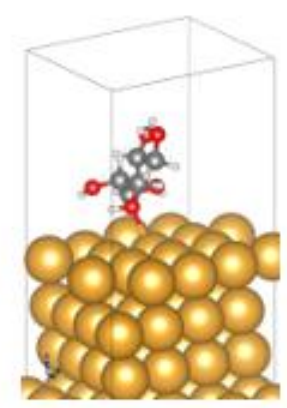	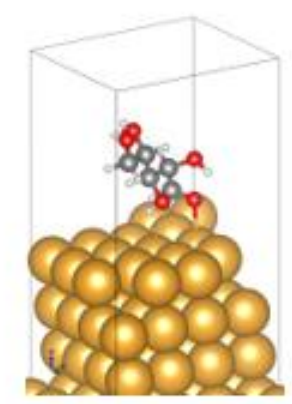	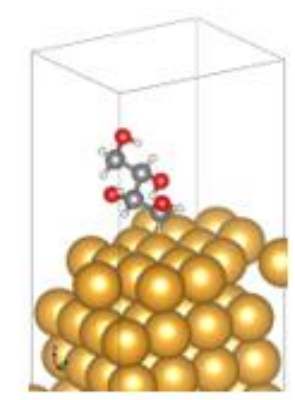
eV	-0.946	-0.067	-0.370	0.179
	Erythrose(C4)	C4→C3	DHA(C3)	
Structure	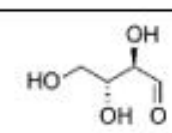	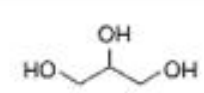	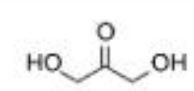	
Adsorption model	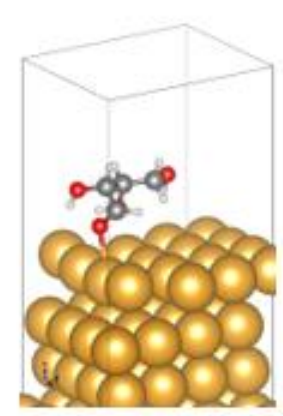	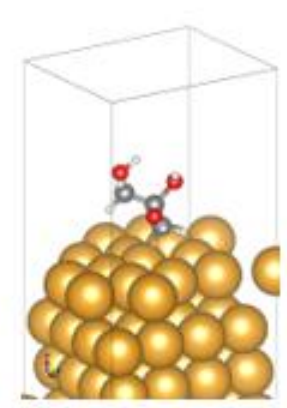	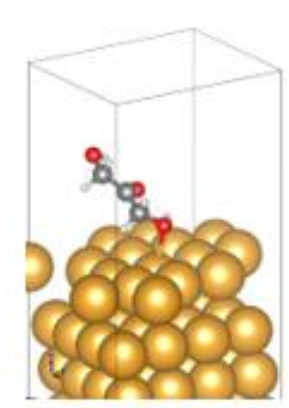	
eV	-0.213	0.524	-0.005	

b

@AuOH	Glucose(C6)	C6→C5	Arabinose(C5)	C5→C4
Structure	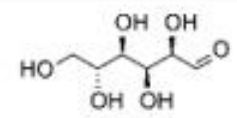	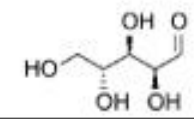	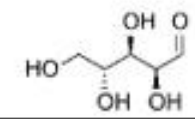	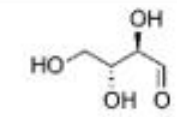 + H_2O
Adsorption model	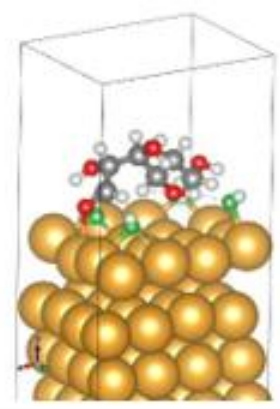	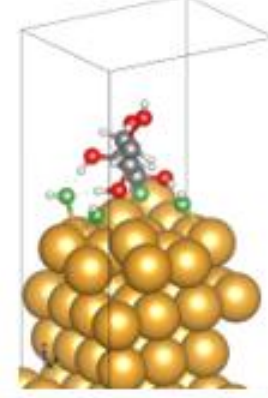	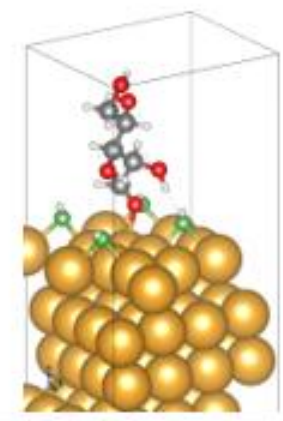	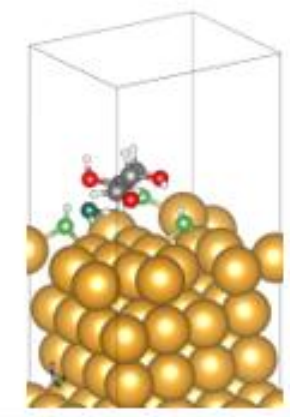
eV	-2.137	-0.965	-0.535	-1.150
	Erythrose(C4)	C4→C3	DHA(C3)	
Structure	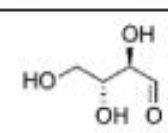	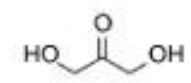 + H_2O	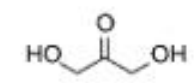	
Adsorption model	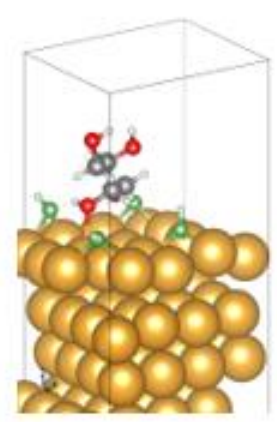	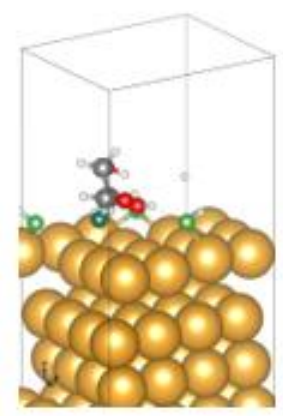	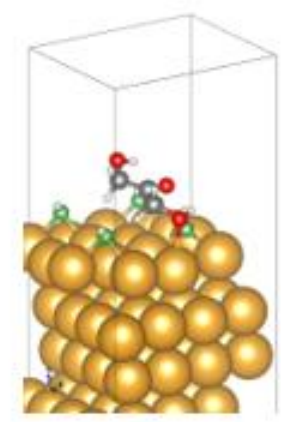	
eV	-0.694	-0.601	-0.448	

c

@CuO	Glucose(C6)	C6→C5	Arabinose(C5)	C5→C4
Structure	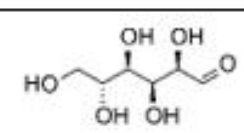	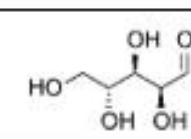 + H_2O	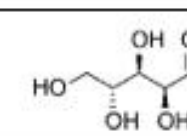	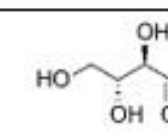 + H_2O
Adsorption model	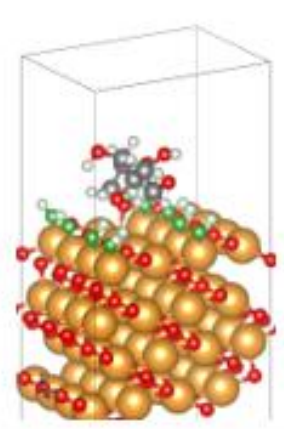	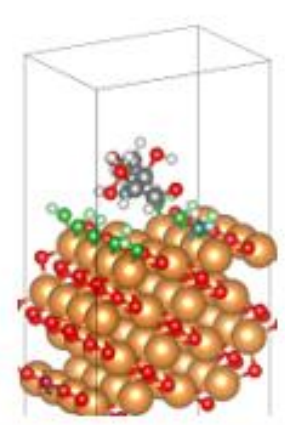	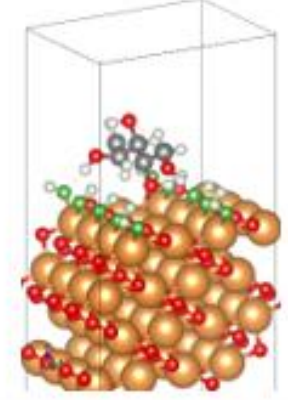	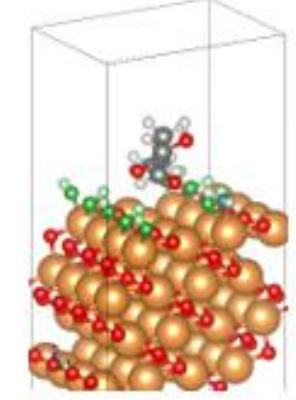
eV	-1.127	-1.823	-1.655	-1.140
	Erythrose(C4)	C4→C3	DHA(C3)	
Structure	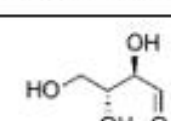	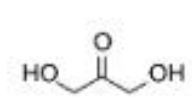 + H_2O	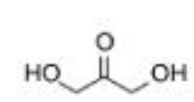	
Adsorption model	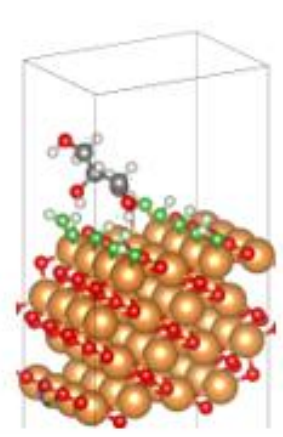	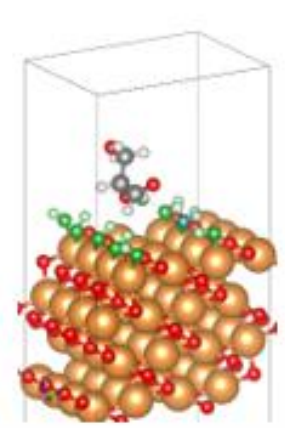	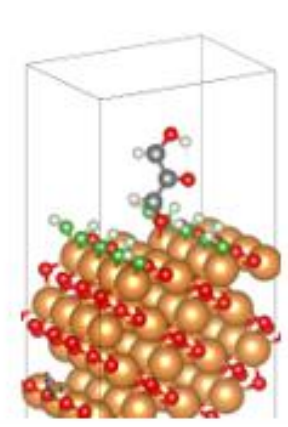	
eV	-0.360	-0.591	-0.250	

Table S3. Optimized structures for two oxidation sequences of glucose: oxidation of the alcohol group followed by oxidation of the aldehyde group, and oxidation of the aldehyde group followed by oxidation of the alcohol group. (a) Au(111), alcohol-first oxidation; (b) Au(111), aldehyde-first oxidation; (c) Au(OH)(111), alcohol-first oxidation; (d) Au(OH)(111), aldehyde-first oxidation; (e) CuO(111), alcohol-first oxidation; (f) CuO(111), aldehyde-first oxidation.

a

@Au	Glucose	a1	a2	a3
Structure	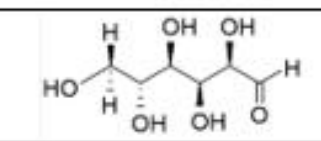	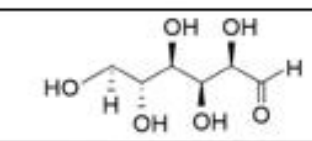	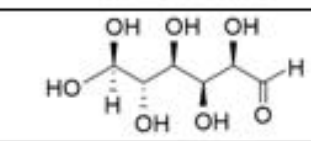	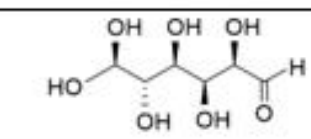
Adsorption model	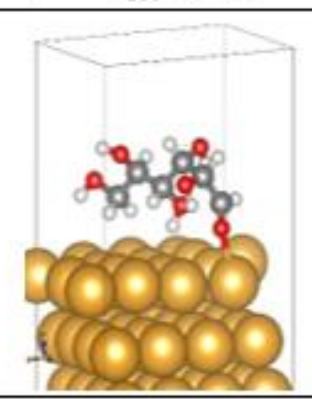	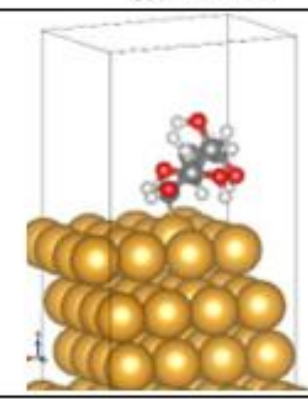	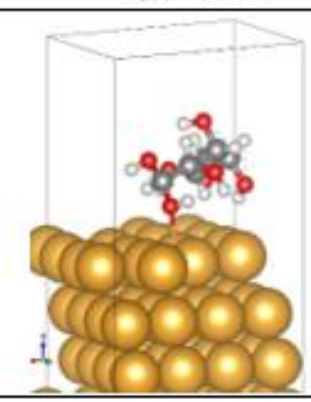	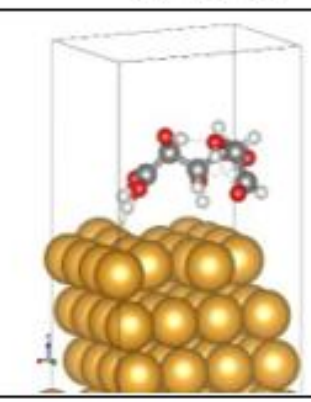
eV	-0.946	-0.028	-0.392	0.294
	Glucuronic acid	a4	Glucaric acid	
Structure	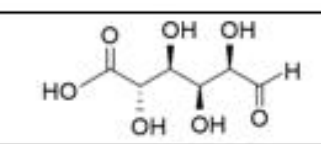	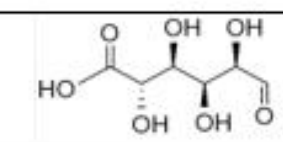	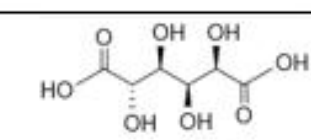	
Adsorption model	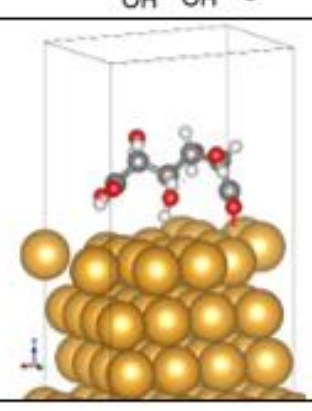	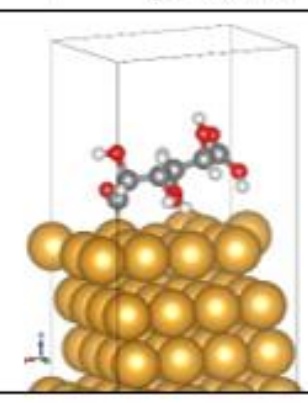	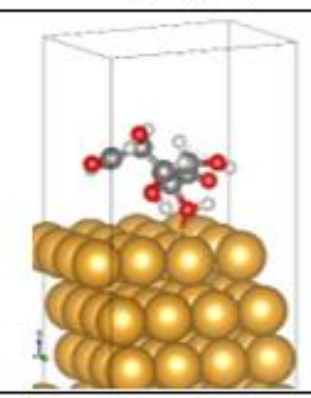	
eV	-0.223	0.631	-0.396	

b

@Au	Glucose	b1	Gluconic acid	b2
Structure	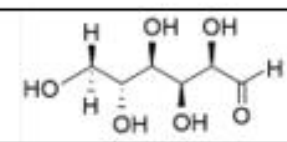	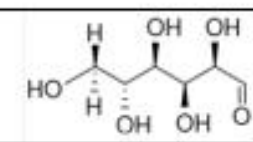	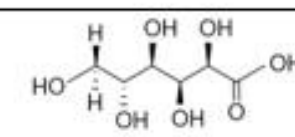	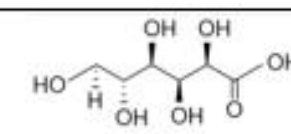
Adsorption model	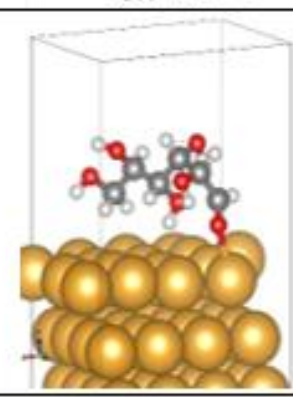	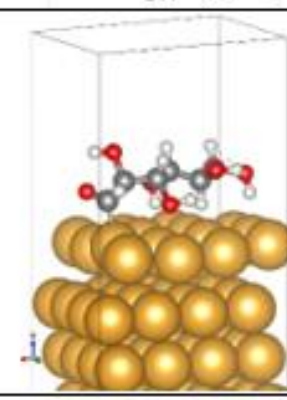	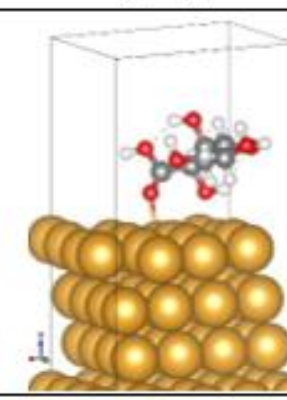	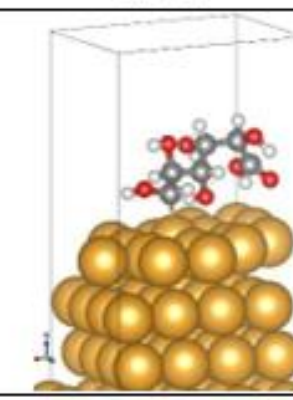
eV	-0.946	-0.378	-0.717	0.041
	b3	b4	Glucaric acid	
Structure	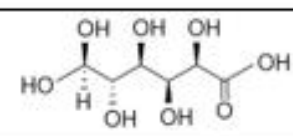	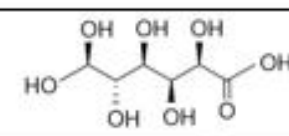	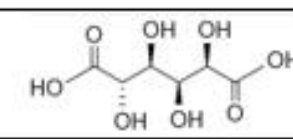	
Adsorption model	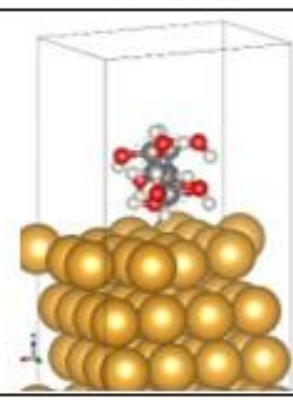	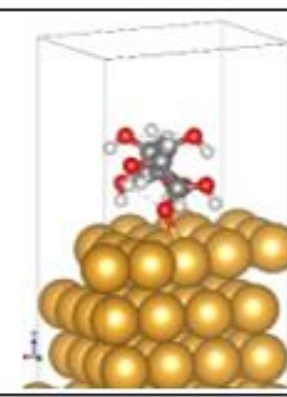	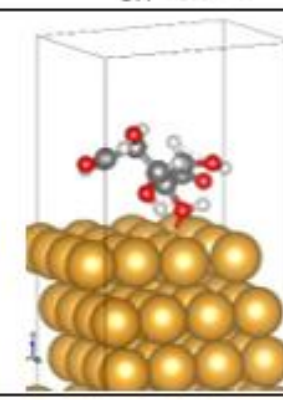	
eV	-0.627	0.324	-0.396	

c

@AuOH	Glucose	a1	a2	a3
Structure	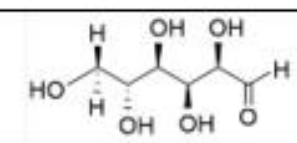	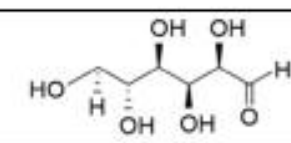	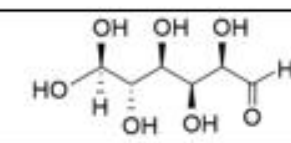	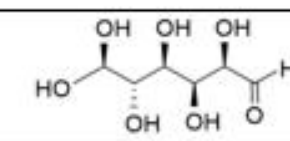
Adsorption model	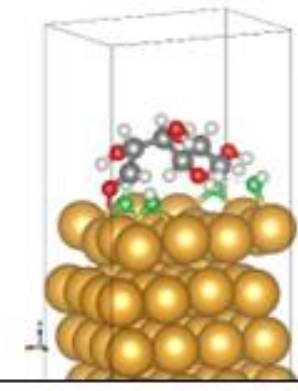	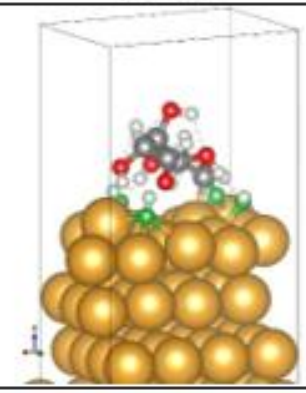	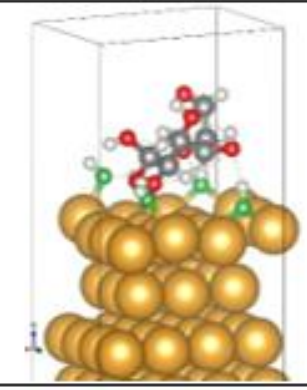	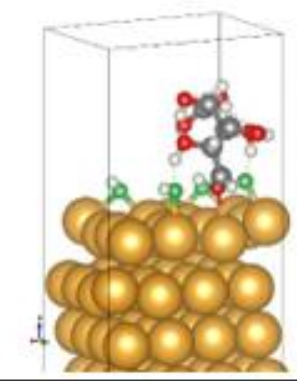
eV	-2.137	-0.823	-1.261	0.570
	Glucuronic acid	a4	Glucaric acid	
Structure	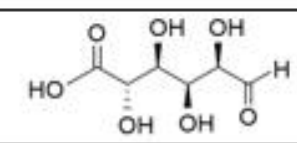	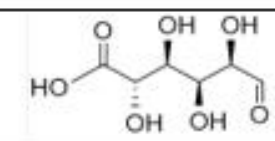	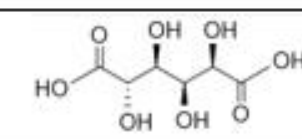	
Adsorption model	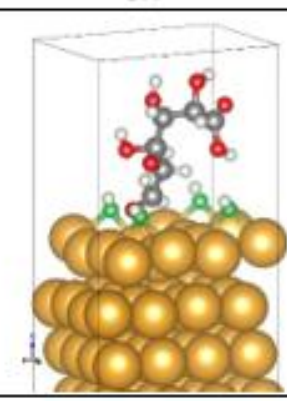	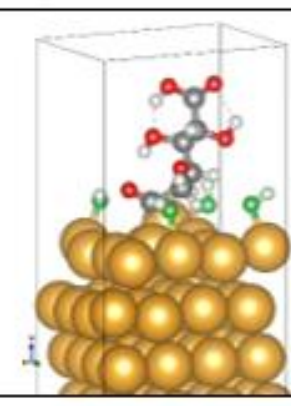	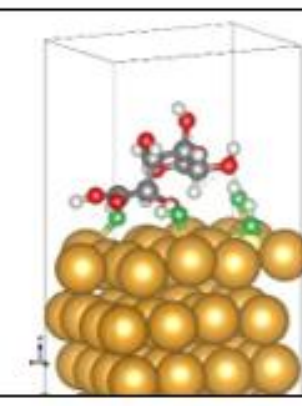	
eV	-0.385	0.110	-1.406	

d

@AuOH	Glucose	b1	Gluconic acid	b2
Structure	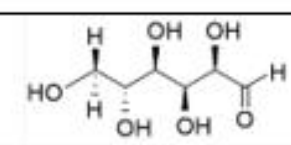	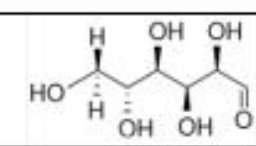	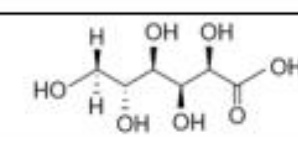	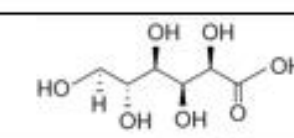
Adsorption model	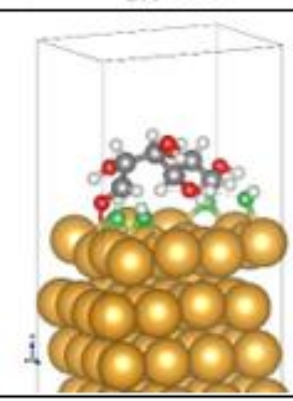	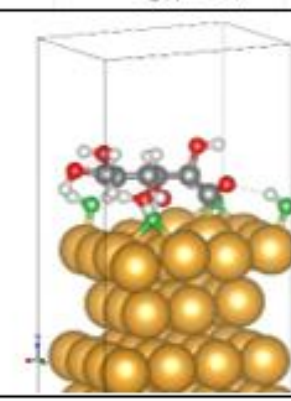	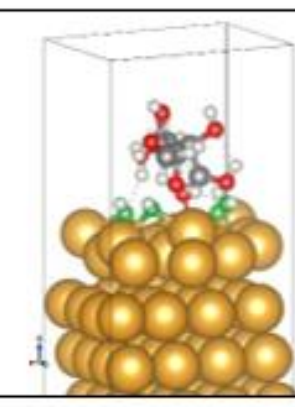	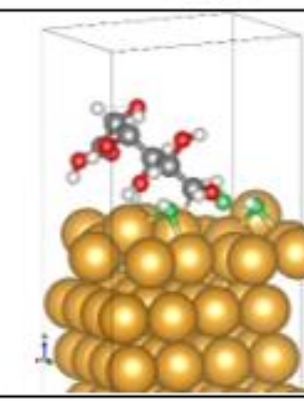
eV	-2.137	-1.239	-1.923	-0.969
	b3	b4	Glucaric acid	
Structure	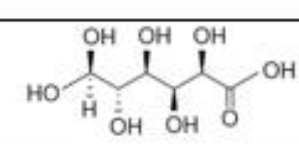	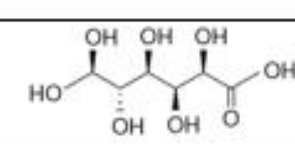	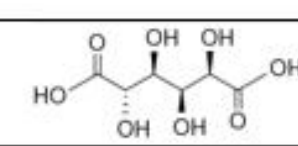	
Adsorption model	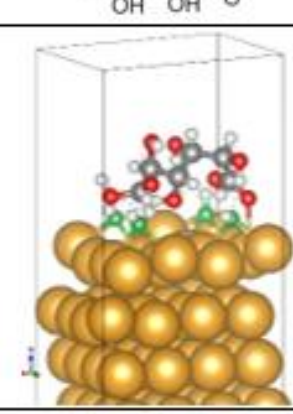	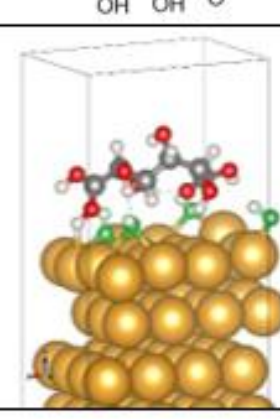	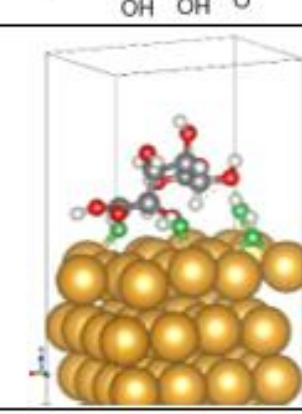	
eV	-1.453	-0.258	-1.406	

e

@CuO	Glucose	a1	a2	a3
Structure	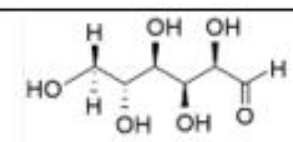	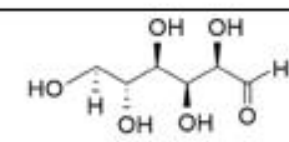	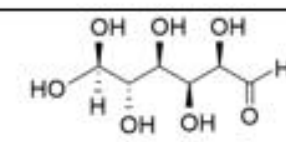	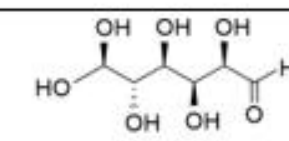
Adsorption model	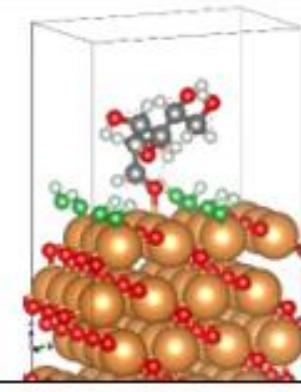	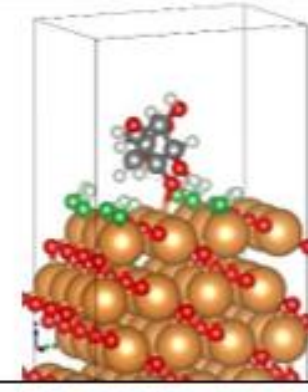	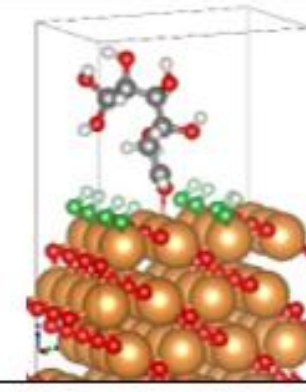	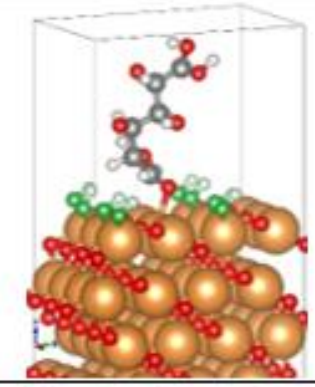
eV	-1.127	0.737	-0.044	1.925
	Glucuronic acid	a4	Glucaric acid	
Structure	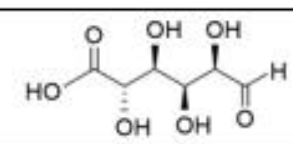	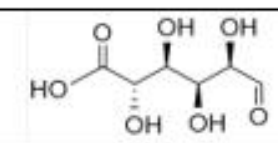	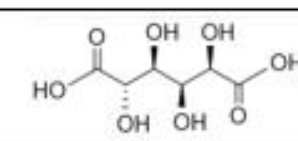	
Adsorption model	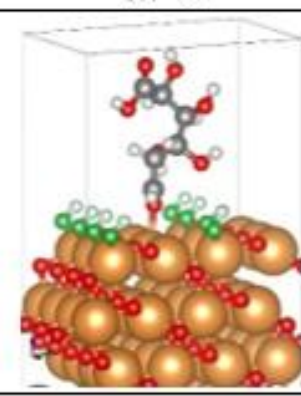	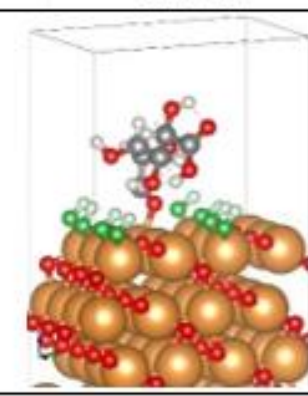	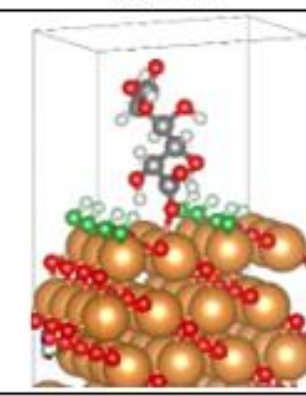	
eV	-0.020	1.696	-0.242	

f

@CuO	Glucose	b1	Gluconic acid	b2
Structure	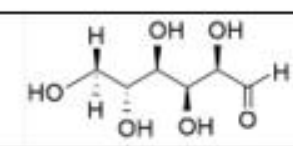	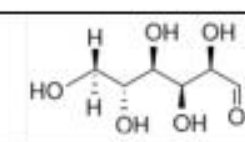	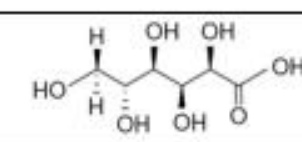	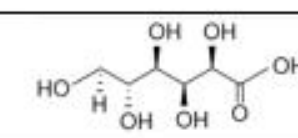
Adsorption model	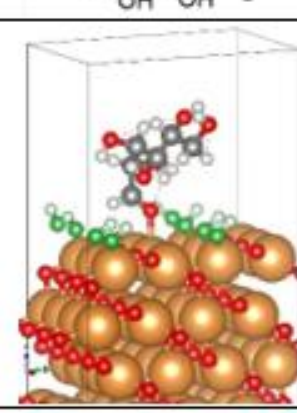	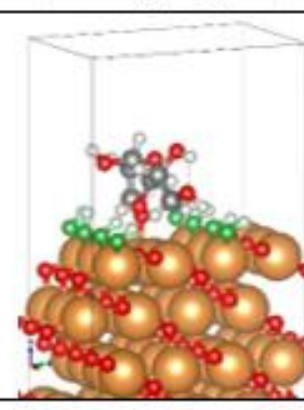	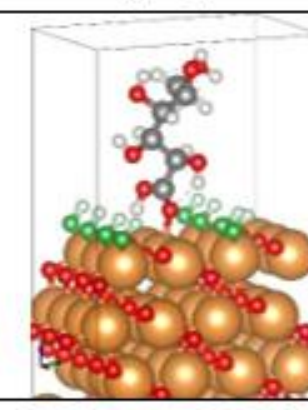	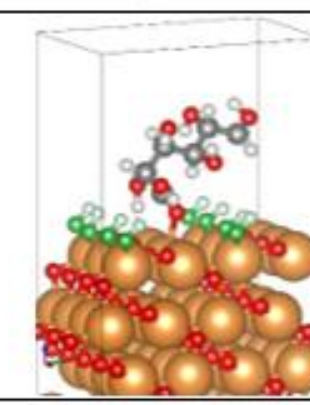
eV	-1.127	2.102	-1.290	0.747
	b3	b4	Glucaric acid	
Structure	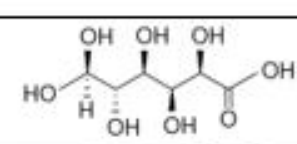	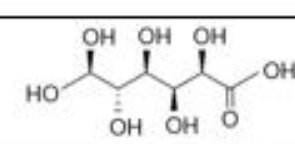	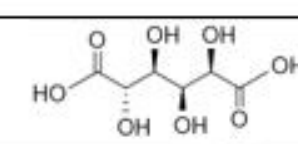	
Adsorption model	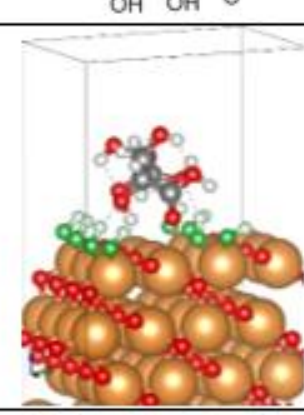	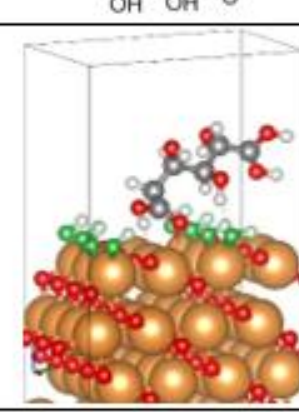	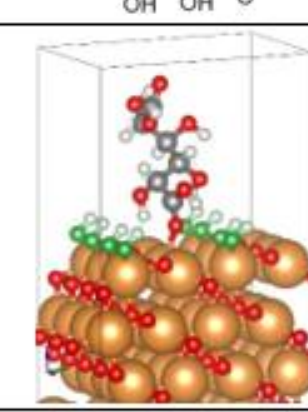	
eV	-0.975	0.603	-0.242	

Table S4. DFT-calculated adsorption energies of glucose in chain and ring configurations on different catalyst surfaces.

**Structure**	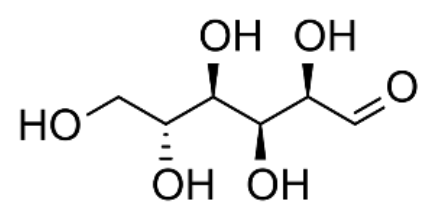	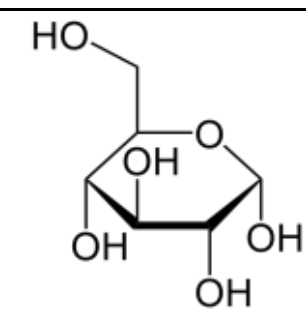	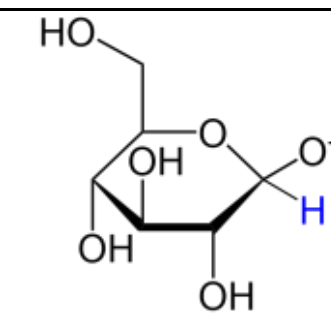
**Au**	-0.946 eV	-0.991 eV	+0.132 eV
**Au(OH)**	-2.042 eV	-1.907 eV	-0.247 eV
**CuO**	-1.127 eV	-1.070 eV	+1.031 eV

Table S5. Optimized structures for the step-by-step simulation of ring opening and oxidation of deprotonated pyranose glucose on Au(111), Au(OH)(111), and CuO(111) surfaces. The optimized geometries on (a) Au, (b) Au(OH), and (c) CuO surfaces are shown.

a

@Au	pH13_Glucose		Carbonyl	
Structure	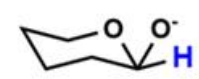	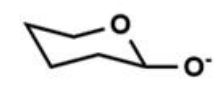	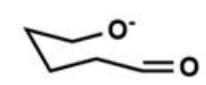	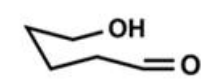
Adsorption model	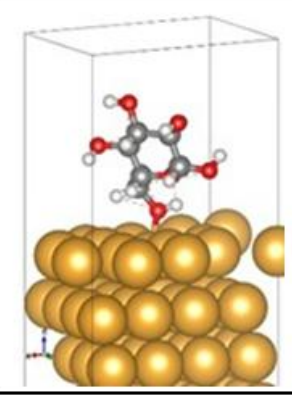	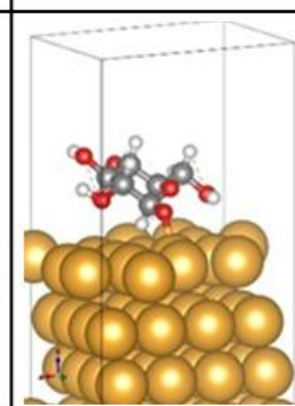	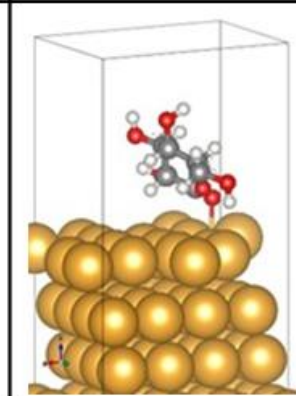	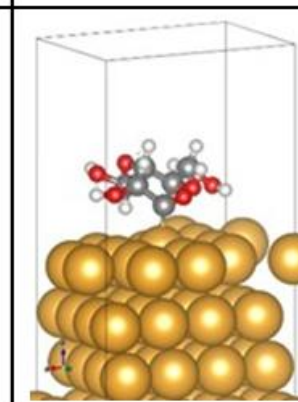
eV	-0.463	0.110	-0.417	-0.166

	Diol(monoanion)	Gluconate
Structure	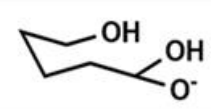	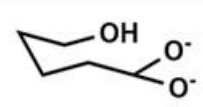
Adsorption model	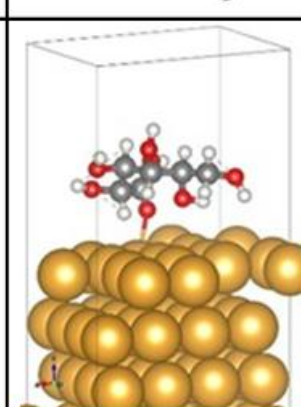	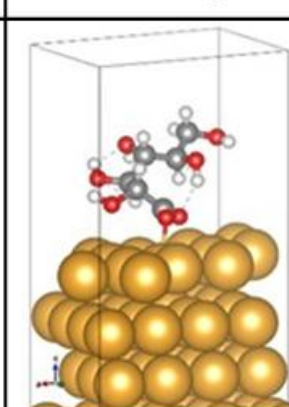
eV	-0.902	-0.172

b

@Au(OH)	pH13_Glucose		Carbonyl	
Structure	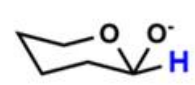	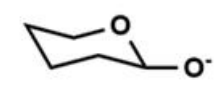	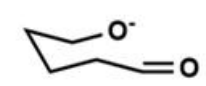	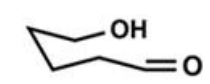
Adsorption model	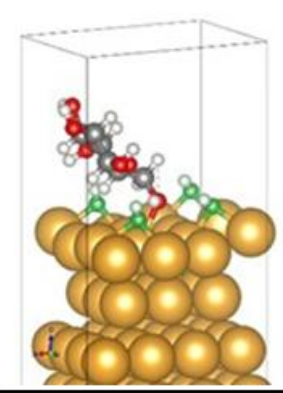	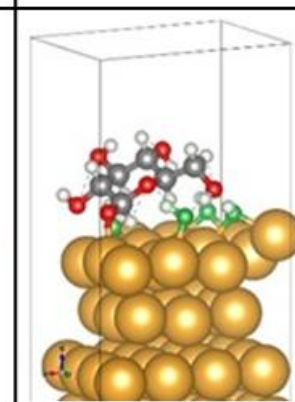	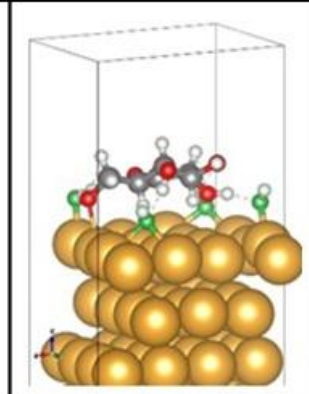	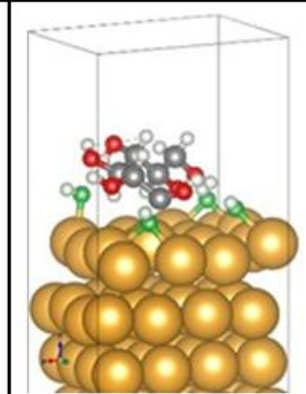
eV	-1.216	-0.608	-0.759	-0.814

	Diol(monoanion)	Gluconate
Structure	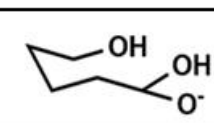	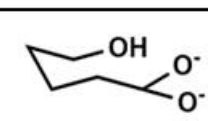
Adsorption model	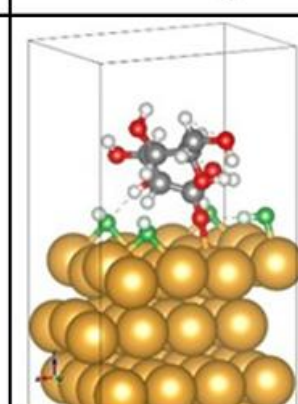	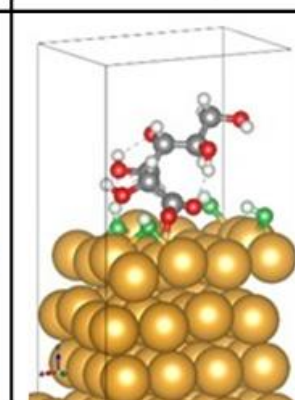
eV	-0.729	-0.457

c

@Cu	pH13_Glucose		Carbonyl	
Structure	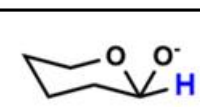	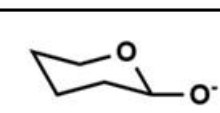	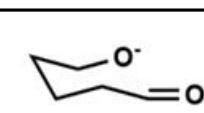	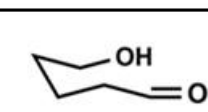
Adsorption model	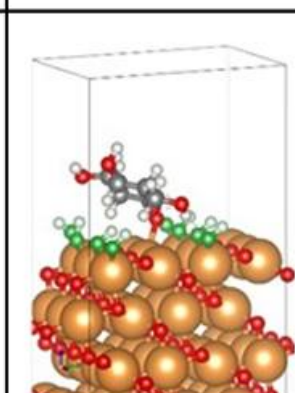	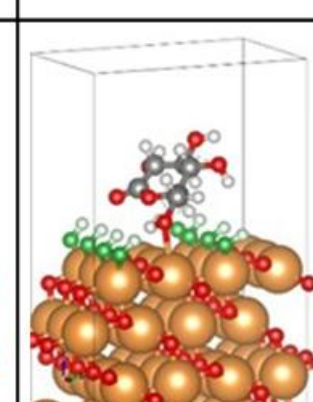	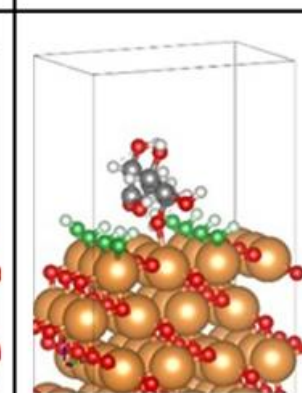	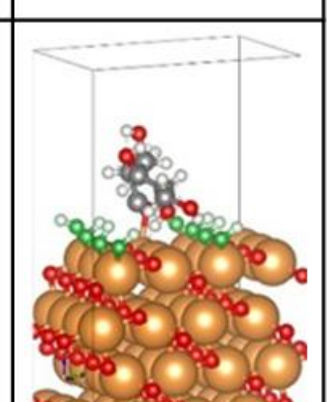
eV	1.031	-1.107	0.406	0.446

	Diol(monoanion)	Gluconate
Structure	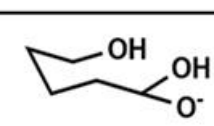	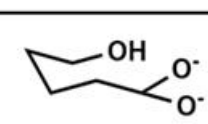
Adsorption model	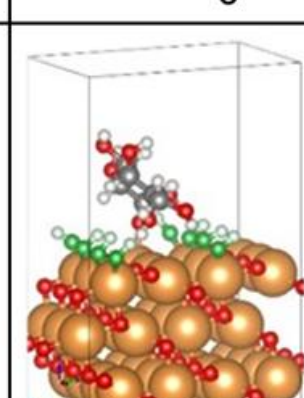	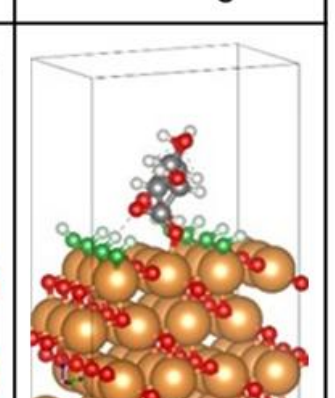
eV	-1.036	0.578

Table S6. Quantification of Cu and Au leaching after 24 h glucose oxidation by ICP-OES.

Catalysts	Cu		Au	
Electrolyte	0.1 M Na_2CO_3	*0.1 M NaOH	0.1 M Na_2CO_3	0.1 M NaOH
Metal leaching amount (ppm)	41.315	62.042	N.D.	N.D.

* The pH decreased from 13 to 6.7, at which point further oxidation was no longer sustained. Therefore, unlike other conditions (24 h), the reaction under this condition was analyzed after 1.5 h.

References

[1] C. Fontana, G. Widmalm, Primary Structure of Glycans by NMR Spectroscopy, Chem. Rev., 123 (2023) 1040-1102.
[2] R.U. Lemieux, J.D. Stevens, The proton magnetic resonance spectra and tautomeric equilibria of aldoses in deuterium oxide, Can. J. Chem., 44 (1966) 249-262.
